# Early Diversification of Membrane Intrinsic Proteins (MIPs) in Eukaryotes

**DOI:** 10.1093/gbe/evae164

**Published:** 2024-07-26

**Authors:** Iker Irisarri, Héctor Lorente-Martínez, Jürgen F H Strassert, Ainhoa Agorreta, Rafael Zardoya, Diego San Mauro, Jan de Vries

**Affiliations:** Department of Applied Bioinformatics, University of Goettingen, Institute for Microbiology and Genetics, 37077 Göttingen, Germany; Campus Institute Data Science (CIDAS), 37077 Göttingen, Germany; Section Phylogenomics, Centre for Molecular Biodiversity Research, Leibniz Institute for the Analysis of Biodiversity Change (LIB), Museum of Nature, 20146 Hamburg, Germany; Department of Biodiversity Ecology and Evolution, Faculty of Biological Sciences, Complutense University of Madrid, 28040 Madrid, Spain; Evolutionary and Integrative Ecology, Leibniz Institute of Freshwater Ecology and Inland Fisheries, 12587 Berlin, Germany; Department of Biodiversity Ecology and Evolution, Faculty of Biological Sciences, Complutense University of Madrid, 28040 Madrid, Spain; Department of Biodiversity and Evolutionary Biology, Museo Nacional de Ciencias Naturales (MNCN-CSIC), 28006 Madrid, Spain; Department of Biodiversity Ecology and Evolution, Faculty of Biological Sciences, Complutense University of Madrid, 28040 Madrid, Spain; Department of Applied Bioinformatics, University of Goettingen, Institute for Microbiology and Genetics, 37077 Göttingen, Germany; Campus Institute Data Science (CIDAS), 37077 Göttingen, Germany; Goettingen Center for Molecular Biosciences (GZMB), Department of Applied Bioinformatics, University of Goettingen, 37077 Göttingen, Germany

**Keywords:** aquaporin, deep eukaryote evolution, aquaglyceroporin, last eukaryotic common ancestor, water transport

## Abstract

Membrane intrinsic proteins (MIPs), including aquaporins (AQPs) and aquaglyceroporins (GLPs), form an ancient family of transporters for water and small solutes across biological membranes. The evolutionary history and functions of MIPs have been extensively studied in vertebrates and land plants, but their widespread presence across the eukaryotic tree of life suggests both a more complex evolutionary history and a broader set of functions than previously thought. That said, the early evolution of MIPs remains obscure. The presence of one GLP and four AQP clades across both bacteria and archaea suggests that the first eukaryotes could have possessed up to five MIPs. Here, we report on a previously unknown richness in MIP diversity across all major eukaryotic lineages, including unicellular eukaryotes, which make up the bulk of eukaryotic diversity. Three MIP clades have likely deep evolutionary origins, dating back to the last eukaryotic common ancestor (LECA), and support the presence of a complex MIP repertoire in early eukaryotes. Overall, our findings highlight the growing complexity of the reconstructed LECA genome: the dynamic evolutionary history of MIPs was set in motion when eukaryotes were in their infancy followed by radiative bursts across all main eukaryotic lineages.

**Significance**

Aquaporins and aquaglyceroporins are important proteins for the transport of water and small solutes and occur in virtually every organism. While the diversity and evolution of this protein family have been dominated by studies in vertebrates and land plants, some studies looked more broadly across the tree of life, fueled by the development of high throughput sequencing and genome initiatives. By interrogating genomic data from unicellular eukaryotes, we identify a previously underappreciated diversity of this gene family. Three clades of aquaporins can be traced back to the common ancestor of eukaryotes, which likely had a complex repertoire of aquaporins and aquaglyceroporins. This speaks of their relevance during the origin and early evolution of eukaryotes.

## Introduction

Eukaryogenesis was a successful evolutionary event that enabled an astonishing diversification of more complex life (Cavalier-Smith 2014). One of the conundrums in early eukaryote evolution is that their last common ancestor is inferred to have possessed a wide range of features. Almost any feature that one would deem a hallmark of eukaryotes was likely present in the last eukaryotic common ancestor (LECA). These include, for example, mitochondria, complex cell cycle with meiosis, intricate intracellular organization with an endomembrane system and organelles, actin- and tubulin-based cytoskeleton enabling intracellular trafficking and cell motility, nucleus with linear chromosomes and different chromatin states, and regulation of gene expression (Eme et al. 2017). Building on genome data across the eukaryotic tree of life, it is now possible to piece together the conserved genetic framework for these important traits. A recent comparative genomics study inferred a complex genome for LECA with ∼13,000 genes (Vosseberg et al. 2021), suggesting a diversified repertoire of gene families. Membrane intrinsic proteins (MIPs) are likely a point in case despite their origin and early evolution remaining obscure.

MIPs are channel proteins with key physiological roles as transporters of water and small solutes across biological membranes, where they form pores as tetramers, each with six transmembrane helices (Verkman et al. 1996; Fu et al. 2000). Their existence was predicted after experimentally measured water transport rates across membranes exceeded estimates based on simple diffusion (Connolly et al. 1998). Because they are present in all species (with few exceptions (Abascal et al. 2014; Tesan et al. 2021)), MIPs are important drug targets for human disease (Soveral and Casini 2017) and central in the development of drought-tolerant crops (Maurel et al. 2015). MIPs have an ancient origin and are highly diversified (Heymann and Engel 1999; Abascal et al. 2014). Most bacteria and archaea generally have one aquaporin (AQP) and one aquaglyceroporin (GLP) that function as water and glycerol transporters, respectively (Ishibashi and Sasaki 1998; Abascal et al. 2014), but up to five major MIP clades have been described in bacteria and archaea (AQPZ, AQPM, AQPN, AQPX, GLPF) (Abascal et al. 2014; Finn et al. 2014; Finn and Cerdà 2015). By contrast, eukaryotes display a much richer set of MIPs that are very diverse in terms of structure and function. The expansion of eukaryotic MIPs is often linked to tissue-specific expression in multicellular organisms (sub-functionalization) and less frequently to functional divergence (Zardoya et al. 2002; Ishibashi 2006; Ishibashi et al. 2011). Most MIP diversity and functions have been studied in vertebrates (AQP0–16) (Finn et al. 2014; Finn and Cerdà 2015; Yilmaz et al. 2020; Lorente-Martínez et al. 2023) and flowering plants (GIP, HIP, NIP, PIP, SIP, TIP, and XIP) (Maurel et al. 2008; Li et al. 2022), with ∼24 described MIP subfamilies in total. Yet, this is a mere fraction of eukaryotic MIP diversity. With the advent of newly sequenced genomes, MIP diversity has been increasingly explored in other eukaryotic groups such as invertebrate animals (Finn et al. 2015; Martínez-Redondo et al. 2023), fungi (Pettersson et al. 2005), green algae (Anderberg et al. 2011), diatoms (Khabudaev et al. 2014), the oomycete *Phytophthora* (Azad et al. 2021), and kinetoplastid parasites (Tesan et al. 2021). These studies often report on new MIP subfamilies outside of the known MIP diversity and are suggestive of a large yet unknown MIP diversity in understudied eukaryotic lineages.

Here, we report on the hidden diversity of MIPs across the eukaryotic tree of life. Our study takes advantage of recently available genomic data for all major eukaryotic supergroups—most of which consist of unicellular protists—to better understand the diversity and evolution of this important protein family. By constructing a phylogenetic framework of thousands of MIPs, we provide a comprehensive view of their diversity. Our data pinpoint deep orthologous relationships among MIP clades that remained uncovered in taxonomically restricted studies; we highlight that the deep diversification of MIPs can be dated back to the infancy of eukaryote evolution.

## Results and Discussion

### A Previously Unrecognized Diversity of Eukaryotic MIPs

**Fig. 1.**
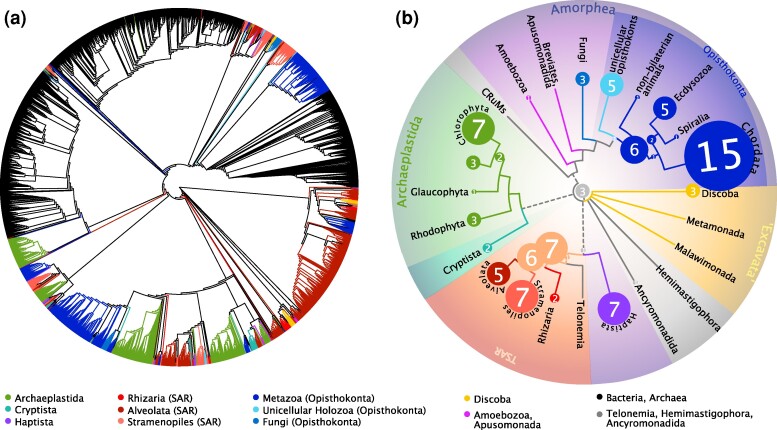
Overview of MIP diversity. a) Maximum likelihood phylogeny of 7,541 proteins (full dataset, midpoint rooting). Branch colors reflect eukaryotic supergroups according to (b); bacteria and archaea are in black (a detailed version of this tree is shown in supplementary fig. S1, Supplementary Material online). b) Eukaryotic MIP paralog clades defined in this study (circles) and their inferred evolutionary origins mapped onto the eukaryotic tree of life (Burki et al. 2020) (dotted lines represent uncertain phylogenetic relationships). Eukaryotic MIP paralog clades include previously described subfamilies and new clades defined here; the three MIP deep clades (MDC1–3) hypothesized to originate in eukaryotes are shown at the tree root. The origin of an MIP paralog group is defined by the most recent common ancestor (MRCA) of all included proteins (i.e. assuming gene loss); for clarity, some taxonomically restricted MIP clades were summarized at higher-level lineages (reconstructed MRCAs for each MIP clade are available in supplementary table S1, Supplementary Material online).

**Fig. 2.**
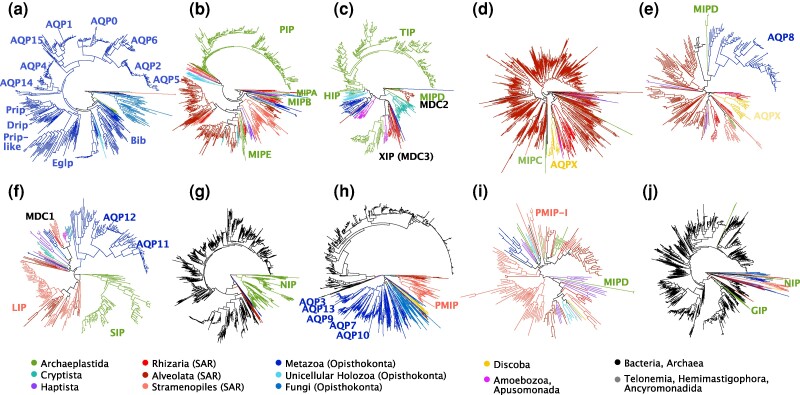
Schematic representation of the maximum likelihood phylogenies (midpoint rooting) of the ten data subsets (a–j) showing the diversity of MIPs. Described MIP groups and here-defined MIP deep clades (MDC1, MDC2, MDC3) are highlighted; branch colors reflect taxonomic affiliation according to the legend. Note that the number of terminals in the tree cannot be interpreted quantitatively as MIP diversity, given the still biased taxonomic sampling in source datasets. Detailed versions of these trees with additional information and evolutionarily or functionally relevant residues highlighted are shown in supplementary figs. S2 to S11, Supplementary Material online.

We assembled a large dataset of 7,541 MIP homologs (Fig. 1a, supplementary fig. S1, Supplementary Material online) aiming to represent the protein family diversity and emphasizing previously overlooked eukaryotic lineages (a total of 4,235 eukaryotic proteins from 484 species). Our dataset builds from a large set of eukaryotic proteins (Richter et al. 2022) as well as previous broad datasets for eukaryotes, bacteria, and archaea (Anderberg et al. 2011; Abascal et al. 2014), and the MIPdb database (Delamarche and Le Bechec; http://genoweb1.irisa.fr/). The dataset was subjected to tree-based decontamination steps in order to remove likely contaminants and symbionts that could bias the inferred MIP diversity patterns. The dataset was split into ten phylogenetically defined subsets (supplementary fig. S1, Supplementary Material online) in an attempt to increase internal phylogenetic resolution in otherwise short protein alignments. The ten subsets were analyzed by maximum likelihood (ML) (Fig. 2) and used to define MIP paralog clades on the basis of phylogeny and the conservation of amino acid residues of functional or evolutionary relevance. We used previously defined MIP groups—for the most part in animals and plants—as anchors to understand both their evolutionary origin as well as the broader eukaryotic MIP diversity, which has not been thoroughly assessed. We performed a preliminary phylogenetic delimitation of eukaryotic MIP diversity by defining MIP paralog clades as the taxonomically most comprehensive clades with SH-aLRT branch support ≥0.85; supplementary figs. S2 to S13, Supplementary Material online). In functionally characterized MIPs, the selectivity of transport depends on two sets of conserved amino acids that define the size and affinity of the pore: two opposed Asn–Pro–Ala (NPA) motifs that form hydrogen bonds with the water molecule and electrostatically repulse protons (Murata et al. 2000), and four residues forming the narrowest pore section that determine substrate specificity (known as the ar/R selectivity filter) (Fu et al. 2000). In addition, five amino acids (Froger's residues or P1–P5) define MIP subfamilies and substrate transport selectivity (Froger et al. 1998).

Our datasets (Fig. 2) recovered all described MIP clades of canonical vertebrate aquaporins (AQP0, 1, 2, 4, 5, 6, 14, and 15), aguaglyceroporins (AQP3, 7, 9, 10, and 13), intracellular aquaporins (AQP11 and AQP12), as well as land plant aquaporins (PIPs, TIPs, XIPs, HIPs, SIPs, and NIPs), and GIPs (Abascal et al. 2014; Finn et al. 2014). AQP16s, proposed as yet another MIP clade closely related to AQP8, was not represented in our final dataset due to its very restricted taxonomic distribution—so far known only from seven tetrapod species (Finn et al. 2014). In agreement with a recent study (Li et al. 2022), we identified the origins of plant TIPs and PIPs to date back to the most recent common ancestor (MRCA) of streptophytes and the MRCA of streptophytes and chlorophytes (Chloroplastida), respectively (supplementary figs. S3 and S4, Supplementary Material online). Our phylogenetic analyses further show that the origin of SIPs can be traced back at least to Chloroplastida and that early duplications occurred in the land plant ancestor (Fig. 2f, supplementary fig. S7, Supplementary Material online). Both NIPs and their early duplications into clades NIP1–4 likely date back to the MRCA of land plants and Zygnematophyceae—land plants’ closest algal relatives (Leebens-Mack et al. 2019) (Fig. 2g, supplementary fig. S8, Supplementary Material online). A putative red algal NIP has also been reported (Li et al. 2022). This implies an earlier origin (and diversification) of NIPs by horizontal gene transfer (HGT) from bacteria (Zardoya et al. 2002; Finn and Cerdà 2015; Pommerrenig et al. 2020). The phylogenetic distribution of MIP paralog clades in Archaeplastida (supplementary fig. S15, Supplementary Material online; supplementary table S2, Supplementary Material online) confirms previous observations that plant AQPs diversified within embryophytes (Abascal et al. 2014).

With regard to animal MIPs, we identified several clades of invertebrate and holozoan MIPs that are orthologous to known vertebrate MIPs: one clade of invertebrate co-orthologs to vertebrate AQP11 and AQP12 (Fig. 2f, supplementary fig. S7, Supplementary Material online) in agreement with (Finn et al. 2015) and two invertebrate orthology groups to vertebrate aquaglyceroporins (Fig. 2h, supplementary fig. S9, Supplementary Material online). These two invertebrate aquaglyceroporin clades were previously identified in deuterostomes (Yilmaz et al. 2020), but we here show that they both likely date back to the origin of animals, given the presence of representatives of all major animal lineages (deuterostomes, ecdysozoans, lophotrochozoans, cnidarias, poriferans, or ctenophorans). These two paralog clades were likely lost in vertebrates, perhaps associated with the gene duplications that gave rise to deuterostome-specific aquaglyceroporins (AQP3, 7, 9, 10, and 13). As previously shown (Finn et al. 2015; Stavang et al. 2015), arthropod aquaporins (Bib, Prip, Prip-like, Drip, and Eglp or entomoglyceroporins that have secondarily evolved glycerol selectivity in insects) are closely related to canonical vertebrate aquaporins (supplementary figs. S2 and S12, Supplementary Material online). Our tree supports deep orthologous relationships of entomoglyceroporins with other pancrustacean and lophotrochozoan MIPs. Bibs and Prips cluster with other spiralian, cnidarian, or poriferan MIPs, which might suggest deeper origins than previously appreciated for these clades (Finn et al. 2015; Stavang et al. 2015; Catalán-García et al. 2021). To help identify key residues in each of the 127 defined MIP paralog clades beyond NPA motifs, ar/R filters, and Froger's residues, we visualized amino acid conservation (supplementary data S1, Supplementary Material online) and identified residues conserved in ≥90% of the sequences in the clade, with numbers referencing the coordinates of the *Escherichia coli* AQPZ sequence (supplementary table S4, Supplementary Material online). We further identified representative sequences in each of the 127 MIP paralog clades as those retaining every conserved amino acid (supplementary table S5, Supplementary Material online).

Beyond animal and plant MIPs, we identify all other described MIP subfamilies including the five green algal MIPs lineages MIPA–E that remain restricted to Chlorophyta (Anderberg et al. 2011), fungal aquaporins and aquaglyceroporins (Verma et al. 2014), large intrinsic proteins (LIPs) recently found in diatoms (Khabudaev et al. 2014), *Phytophthora* MIPs (Azad et al. 2021), and kinetoplastid AQPXs (Tesan et al. 2021). LIPs were identified as part of a clade including other ochrophytes beyond diatoms, dinoflagellates, and ciliates and thus dating back at least to the MRCA of Stramenopila + Alveolata within the Telonemia, Stramenopila, Alveolata, Rizaria (TSAR) supergroup (Fig. 2f, supplementary fig. S7, Supplementary Material online), much earlier than initially thought (Khabudaev et al. 2014). The majority of *Phytophthora* MIPs (clades PMIP-A-H) clustered together, and likely originated from ancient duplications within oomycetes (probably the MRCA of Peronosporales and Phytiales), and the entire clade probably dates back to the Stramenopila + Alveolata ancestor within TSAR as suggested by closely related oomycetes and dinoflagellates with conserved amino acid residues (Fig. 2h, supplementary fig. S9, Supplementary Material online). The *Phytophthora* PMIP-I clade originally defined by a single sequence (XP_008909057) (Azad et al. 2021) is further corroborated by two additional species but remains restricted to *Phytophthora* (Fig. 2i, supplementary fig. S10, Supplementary Material online). Kinetoplastid AQPXs are recovered as two distantly-related clades that suggest their origin by an ancient duplication predating Discoba (Fig. 2d and e, supplementary figs. S5 to S6, Supplementary Material online), but the overall low statistical support of deep branches and the fast evolutionary rates of AQPXs (that make them more prone to long-branch attraction artifacts) suggests that this hypothesis should be taken with caution. The possibility of independent origins of two AQPX clades could not be identified by analyzing exclusively discoban proteins (Tesan et al. 2021). In contrast to the two typical NPAs of MIPs, a clade of ochrophyte and dinophycean proteins (SAR IX; Fig. 2d, supplementary fig. S5, Supplementary Material online) has three (sometimes four) conserved NPA residues. To further characterize this, we used HMMER (hmmsearch) and found that in 1,101 out of 7,541 sequences two or more (up to nine) MIP domains could be identified. Upon closer inspection, several instances are only partial MIP domains. However, a large portion of sequences identified by hmmsearch as having more than one MIP domain (624/1,101 or 60%) correspond to the SAR IX clade named above. While it is difficult to assess the reliability of these SAR IX proteins without direct proteomic data, we note that several of them derive from annotated genomes of diverse species in EukProt (Richter et al. 2022). Our analysis of phylodiverse MIPs thus has important implications. There are limitations in the prediction of MIP domains that bear out particularly in certain eukaryotic MIPs. The more interestingly implication, however, is that there likely are unrecognized multi-domain MIPs in certain understudied eukaryotic groups (e.g. TSAR) and even in previously investigated MIP protein diversity. In any case, these proteins deserve further scrutiny in future studies.

MIPs are much more diverse than previously thought: they are found across all major eukaryotic lineages and several new MIP clades lay outside of the previously described subfamilies (Fig. 1b, supplementary table S1, Supplementary Material online). Most of the new clades, defined on the basis of phylogenetic affinity and key residue conservation, correspond to unicellular eukaryotes, which have remained largely understudied with respect to MIPs. Quantitatively, the majority of the new MIP clades correspond to the TSAR supergroup (Strassert et al. 2019), with one MIP clade dating back to its MRCA, seven clades dating back to the SAR ancestor, six further clades to the Stramenopila + Alveolate ancestor, one to each of the MRCAs of Alveolata and Rhizaria, in addition to many taxonomically-restricted clades of dinoflagellates (Alveolata; five clades), ciliates (Alveolata; two clades), ochrophytes (Stramenopila, three clades), diatoms (Stramenopila, two clades), Bigyra (Stramenopila, one clade), and foraminiferans (Rhizaria, one clade). Seven clades of Haptista were recovered containing representatives of either Prymnesiophyceae or Pavlovales. For Cryptista, we found two MIP clades dating back to at least the MRCA of its largest clade Cryptophyceae (no significant MIP homologs were found in Centrohelida transcriptomes). In Archaeplastida, one clade of Glaucophyta and three clades of Rhodophyta were recovered; two out of the three clades in Rhodophyta likely date back to the MRCA of the group. A second trypanosomatid (Discoba) MIP clade was recovered in addition to the previously described AQPXs (Tesan et al. 2021). In Amoebozoa, a new MIP clade was identified for slime molds (Eumycetozoa). We also found a clade of Ancyromonadida MIPs (Fig. 2b, supplementary fig. S3, Supplementary Material online). One clade of choanoflagellate and filastereran MIPs, four clades of choanoflagellates, and one clade of ichthyosporeans were found for the closest unicellular relatives of animals (Opisthokonta, Holozoa). Interestingly, one sponge MIP clade clusters with a bacterial homolog and might represent an HGT of an aquaglyceroporin in sponges (Kenny et al. 2020), here shown to date back at least to the MRCA of Heteroscleromorpha (Fig. 2i, supplementary fig. S10, Supplementary Material online).

The recovered high diversity of eukaryotic MIPs with large paralog groups outside of the known subfamilies and the non-conservation of functional residues might be suggestive of new roles for some of these MIPs. Besides water, MIPs are known to transport small molecules including non-polar compounds such as glycerol, urea, and lactic acid; reactive oxygen species and hydrogen peroxide; gasses such as ammonia, carbon dioxide, and nitric oxide; and metalloids like boron, silicon, arsenic, and antimony (Gupta and Sankararamakrishnan 2009; Mukhopadhyay et al. 2014). This broad range of compounds highlights the functional versatility of MIPs and their roles in many key cellular processes. In the case of unicellular eukaryotes, the presence of multiple MIPs likely provides a better control of solute transport compared to the passive exchange through the membrane (larger eukaryotic cells have a lower surface-to-volume ratio and thus slower equilibration times) (Tanghe et al. 2006). Furthermore, the presence of specific MIPs for vacuoles (TIPs) and intracellular membranes (SIPs, AQP11, and AQP12) further supports the hypothesis of functional diversification. MIPs are also central for stress responses to abiotic and biotic factors including low temperature and freezing (membrane permeability is reduced) (Tanghe et al. 2006), symbioses with fungi, the formation of resistance forms such as spores (Tanghe et al. 2006), or the maturation of siliceous cell walls in diatoms (Grachev et al. 2008). Further functional and structural studies of the high eukaryotic MIPs diversity are likely to broaden the known set of functions performed by them. For example, trypanosomatid AQPXs are likely poor transporters of water and glycerol and possess wider selectivity filters to permeate larger (so far unknown) solutes (Tesan et al. 2021). From a biotechnological viewpoint, MIPs have been proposed as drug targets against fungal (Verma et al. 2014) and trypanosomatid (Tesan et al. 2021) parasites.

MIPs are not only present across all major eukaryotic supergroups—they diversified within each of them as well. The number of available genomes and transcriptomes per eukaryotic supergroup predicts well the number of MIP homologs in the final dataset (Pearson's *R* = 0.93, *P* = 0.0021; supplementary fig. S14, Supplementary Material online). While a definitive relationship between new MIPs and genomic data availability cannot be inferred here because transcriptomic data do not represent the full gene repertoire of a species, it is clear that additional genomic data on lesser-studied, deep eukaryotic groups is needed to complete the understanding of MIP diversity. These data push MIPs of unicellular eukaryotes into the limelight.

### A Complex Repertoire of MIPs in LECA

**Fig. 3.**
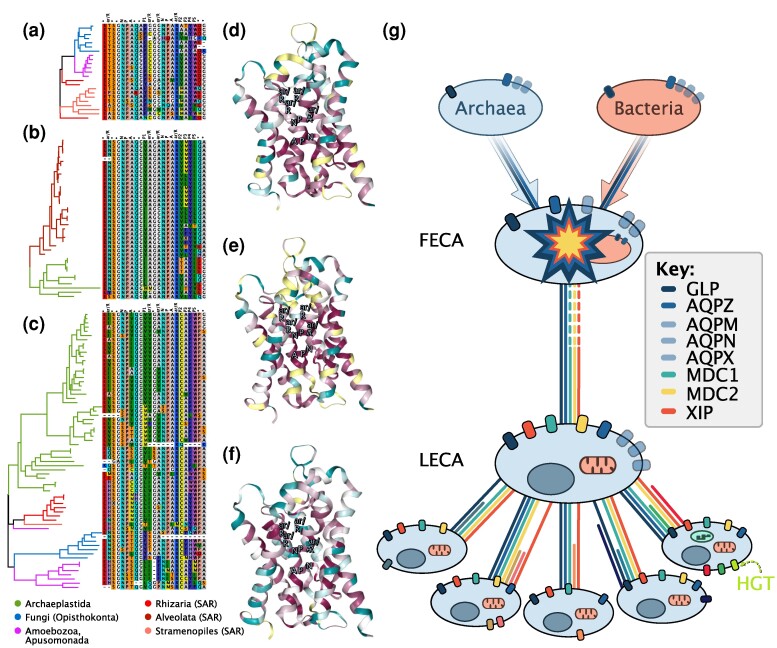
Early evolution of eukaryotic MIPs. Residue conservation for the three MIP clades of likely ancient origin is highlighted in the main text. Alignments highlight key amino acids of functional and evolutionary relevance (tree branches colored by eukaryotic lineage) while structures reflect overall amino acid conservation as inferred with ConSurf for (a, d) MDC1, (b, e) MDC2, (c, f) XIPs or MDC3. (g) Hypothetical scenario for the early evolution of MIPs, showing the transition from FECA to the LECA and early duplications within main eukaryotic supergroups; one HGT event is also shown (figured inspired by Eme et al. (2017)).

Despite overall low statistical support for many deep relationships among defined MIP paralog groups (expected for short proteins that diverged a long time ago), we identified further MIP paralog clades that might date back to ancient gene duplications during the early evolution of eukaryotes. We identified a strongly supported clade coined MDC1 (“MIP deep clade 1”) that groups several distantly related unicellular eukaryotes including slime molds (Amoebozoa, Eumycetozoa), blastocladiomycete and chythridiomycete fungi (Fungi), golden algae (Stramenopila, Ochrophyta), and rhizarians such as *Paulinella* (Rhizaria) (Fig. 2f, supplementary fig. S7, Supplementary Material online; supplementary table S3, Supplementary Material online). Phylogenetically, MDC1 clusters close to unorthodox aquaporins (AQP11 and AQP12), SIPs and LIPs. Froger's residues suggest that MDC1 might represent aquaporins (e.g. small uncharged P2–P3 and aromatic P4–5) but display aromatic amino acids in P1 typical of GLPs; their ar/R filters are also unlike any functionally characterized MIP: small hydrophilic residues in helix 2 and hydrophobic (G) and hydrophilic (R) residues in helix 5 (Fig. 3a and d). Unlike AQP11 and AQP12, which have a functionally-relevant C after the second NPA box (NPAxxxxxxxxC; C197 in *E. coli* AqpZ), MDC1, SIPs, and LIPs lack this cysteine (supplementary table S4, Supplementary Material online), which supports their evolutionary distinctiveness, in agreement with a recent study (Ishibashi et al. 2023). A second clade of deep evolutionary origin (termed MDC2) is formed by chlorophyte algal MIPD (Archaeplastida) and a dinoflagellate (Alveolata) clade (Fig. 2c and b, supplementary fig. S4, Supplementary Material online; supplementary table S3, Supplementary Material online), which received strong statistical support and displays conserved key residues (supplementary table S4, Supplementary Material online). Two additional small algal clades annotated as MIPD (Anderberg et al. 2011) are recovered elsewhere in our trees (Figs. 2e and i, supplementary figs. S6 and S10, Supplementary Material online), but we assume this is likely spurious due to their very long internal branches that make them more prone to phylogenetic artifacts. MDC2 could represent the first deep orthology proposed for any of the mysterious clades named MIPA-E that are exclusively found in green algae (Archaeplastida, Chlorophyta). Residue conservation suggests that MDC2 might be aquaporins (e.g. non-aromatic P1, small uncharged P2–P3, and aromatic P4), but also have non-aromatic P5 as in GLPs. The ar/R filters are well conserved but unusual, with neutral hydrophilic (N) and small hydrophobic (A) residues in helix 2 and hydrophobic (A/G)-hydrophilic (R) residues in helix 5 (Fig. 3b and e). The third deep clade (MDC3) is that of XIPs, which in our analyses encompasses plant XIPs with slime molds and other amoebas (Amoebozoa), chlorarachniophyte algae (Rhizaria), and one diatom (Stramenopila) (Fig. 2c and Fig. 3c and f, supplementary fig. S4, Supplementary Material online; supplementary table S3, Supplementary Material online). A deep evolutionary origin of XIPs has already been proposed based on phylogenetic clustering of plant XIPs with *Dictyostelium* (Amoebozoa) and fungi (Danielson and Johanson 2008; Gupta and Sankararamakrishnan 2009; Abascal et al. 2014). Previously identified fungal XIPs are recovered elsewhere in our analyses (Fig. 2j, supplementary fig. S11, Supplementary Material online) likely due to a long branch attraction artifact, but the conservation of key residues (Fig. 3c and f) and a set of synapomorphic amino acids (Abascal et al. 2014) are strong indicators of their deep orthology. NPA motifs, ar/R filters, and Froger's residues show high variability (Fig. 3c and f), as previously reported for angiosperms, bryophytes, algae, fungi, and *Dyctyostelium* (Amoebozoa) (Gupta and Sankararamakrishnan 2009). All three deep MIP clades are likely salient to very ancient gene duplication events. They contain representatives of both Obazoa (e.g. Opisthokonta, Amoebozoa) and Diaphoretickes (e.g. SAR, Archaeplastida). According to the current understanding of the deep eukaryotic phylogeny (Burki et al. 2020) (Fig. 1b), such duplications date back straight to LECA under the assumption of a “unikont/bikont” root (Derelle et al. 2015), or would date back to the second deepest node in the tree under the assumption of a “neozoan/excavate” root (He et al. 2014). According to the latest molecular clock estimations, these events occurred >2 billion years ago (Strassert et al. 2021).

Two other MIP clades could represent additional instances of deep orthology within Diaphoretickes: one clade of TSAR and Haptista (supplementary fig. S6, Supplementary Material online) and another clade with representatives of TSAR + Cryptista (supplementary fig. S3, Supplementary Material online). However, such genes might be affected by endosymbiotic gene transfer (EGT) events, as the above lineages were involved in the ancient endosymbioses that gave rise to secondary red-algal plastids (Strassert et al. 2021). Abascal et al. (2014) suggested the possibility of deep orthology for plant XIPs + HIPs + TIPs + animal AQP8, and for plant PIPs + animal aquaporins (AQPs 0 to 2, 4 to 6) but also warned about the chance for phylogenetic artifacts and low statistical support for internal tree branches. Ishabashi et al. (2017) considered intracellular SIPs, AQP11, and AQP12 a subfamily on their own (“superaquaporins”) but the non-conservation of key amino acids and the possibility of a long-branch attraction artifact put into question this deep orthology hypothesis (Abascal et al. 2014).

The presence of MIP clades whose origin can be traced back to the deepest nodes in the eukaryotic tree of life is indicative of a dynamic evolutionary pattern of gene duplications, losses, and divergences in the MIP family already very early in eukaryotic evolution. In particular, some of the deep MIP clades might be traceable to LECA, suggesting a complex ancestral repertoire of MIP homologs for the first eukaryotes. Despite the difficulties in reconstructing the evolution of MIPs in bacteria and archaea, mainly due to high rates of HGT, and the lack of phylogenetic component in MIP contents—that appears to largely depend on lifestyle (Abascal et al. 2014)—the identification of four aquaporins (AQPZ, AQPM, AQPN, and AQPX) and one aquaglyceroporin (GLPF) in both bacteria and archaea (supplementary fig. S12, Supplementary Material online) (Finn et al. 2014; Finn and Cerdà 2015) might indicate the presence of up to five MIPs in the first eukaryotic common ancestor (FECA). However, these five MIP lineages show a restricted taxonomic representation among Archaea in our dataset (AQPM and GLPF in Euryarchaea; AQPN in Nitrososphaerota; AQPZ in Euryarchaeota, Nitrososphaerota, and Thaumarchaeaota; no Asgard representatives; supplementary table S6, Supplementary Material online). Bacterial AQPs (AQPZ, AQPM, AQPN, AQPX) might even share a more recent common ancestor distinct from aquaglyceroporins (GLPFs), as proposed based on the longer sequences between the two homologous NPA boxes of the latter group (Ishibashi and Sasaki 1998). This diversity of prokaryotic MIPs contrasts with the fact that most prokaryotes have two, one, or no MIPs (Tanghe et al. 2006; Abascal et al. 2014; Zardoya et al. 2015). Reconciling this with their putative origin in FECA would imply an ancient origin within bacteria followed by rampant gene loss in many lineages of extant bacteria and archaea, but their origin might also be explained by more recent HGT events. In fact, the presence of HGT cannot be excluded before FECA or along the FECA to LECA transition, further complicating the reconstruction of deep orthology relationships and MIP repertoires of these ancestors. Attempts to reconstruct the genomic repertoire of the first eukaryotes have also identified several MIP homologs in LECA (Ku et al. 2015; Pittis and Gabaldón 2016). Our results further indicate a rather dynamic evolution of MIPs in the earliest eukaryotes and point out to the presence of at least five MIP paralogs (MDC1, MDC2, XIPs, or MDC3, plus the ancestral AqpZ, GlpF, and perhaps AqpM, AqpN, and AqpX) very early in eukaryote evolution and probably directly in LECA (Fig. 3g). We stress that this is a preliminary hypothesis for the early evolution of eukaryotic MIPs, given the difficulty of identifying ancient orthology relationships with certainty using inherently short protein alignments. In fact, our proposed scenario is a parsimonious one for the data at hand and additional data could reveal different patterns with further events of deep orthology, gene duplications, losses, and HGTs.

## Conclusions

MIPs have undergone pronounced expansions within most eukaryotic supergroups, largely by gene duplication but also through non-vertical inheritance such as EGT and HGT from bacteria (Fig. 3g). Our hypothesized scenario roughly agrees with the numbers of MIP homologs recovered by a large-scale protein clustering (Ku et al. 2015) that found three ancestral (eukaryote–prokaryote clusters) and 23 eukaryote-specific MIP clusters. The early burst of MIP homologs in eukaryotes is also in line with the high rates of gene duplication inferred for early eukaryotes, which apparently doubled the number of genes in the transition from FECA to LECA (Vosseberg et al. 2021). An early diversity in MIPs suggests an early diversification in function. This diversification likely facilitated complexity in physiological properties in an ancient single-celled eukaryote, allowing a versatile transport of small solutes. Versatility in solute transport through MIPs thus is a cornerstone of eukaryotic functions that can be traced back to LECA—if not to an earlier infancy of eukaryote evolution.

## Materials and Methods

### MIP Dataset Assembly

A large dataset of MIP homologs was assembled from the following sources: (i) the most taxonomically broad analysis of MIPs (Abascal et al. 2014), (ii) bacterial and archaeal homologs in MIPdb (http://mipdb.genouest.org/), (iii) chlorophyte MIPs (Anderberg et al. 2011), and (iv) newly identified eukaryotic MIP homologs from EukProt v.2 (Richter et al. 2022). Thus far, EukProt is the largest and taxonomically broadest collection of eukaryotic genomes and transcriptomes (742 species), with emphasis on unicellular eukaryotes. MIP homologs in EukProt were identified by (i) BLASTP v2.11.0 searches (Altschul et al. 1990) using 105 MIP proteins from *Homo sapiens*, *Arabidopsis* spp., *Trypanosoma* spp. and *Capsaspora owczarzaki* as queries that represent the known MIP diversity (e-value threshold: 1e-20), and (ii) HMMER v3.3 (Finn et al. 2011) searches using Pfam's MIP Hidden Markov model (HMM) profile (PF00230). In both cases, hits were required to align at least 150 amino acids. MIP homologs from all four sources were merged into a single dataset of 8,308 proteins, aligned with MAFFT v7.304 (Katoh and Standley 2013) (“–auto”; FFT-NS-2 iterative alignment) and alignment columns with >90% missing data were removed with trimAL v1.3 (Capella-Gutiérrez et al. 2009). A ML phylogeny was inferred with IQ-TREE v1.6.12 (Nguyen et al. 2015) under BIC-selected best-fit substitution model LG + F + Γ4 and branch support assessed with 1,000 replicates each of ultrafast bootstrapping (UFBoot) (Hoang et al. 2018) and SH-like approximate likelihood ratio test (SH-aLRT) (Guindon et al. 2010).

### Dataset Decontamination

The initial tree was used to identify and remove duplicates and likely contaminants based on unexpected phylogenetic clustering. For this, the tree was visually inspected with FigTree v1.4.3 (http://tree.bio.ed.ac.uk/software/figtree/) assigning distinct colors to the different eukaryotic supergroups as obtained from the NCBI taxonomy (Schoch et al. 2020). Proteins that clustered outside of their respective eukaryotic lineage (“supergroup”) were excluded, except when three or more different species were present. Small clades containing a mix of very different eukaryotic groups where BLASTP-searched against NCBI‘s non-redundant (NR) database and removed if their best hits were from a different eukaryotic supergroup. After a first round of decontamination, the resulting full-length clean dataset was re-aligned and a new ML tree was constructed as detailed above. The tree was used to select 18 subsets of more closely related sequences, for which independent ML trees were built upon re-alignment and trimming (as detailed above) to facilitate identification of contaminants among less divergent proteins. The 18 cleaned full-length subsets were combined, re-aligned, trimmed, and a new inclusive ML tree was inferred as detailed above. A third round of decontamination specifically targeted known endosymbionts or food sources, as detected in a previous study that used EukProt data (Strassert et al. 2021). For example, dinoflagellates (Alveolata) inside ochrophytes (Stramenopila) or *Sorites* (Rhizaria) could be endosymbionts; *Paramecium* clustering in algal clades are likely symbionts; *Tiarina* transcriptomes are highly contaminated, often with ochrophytes and diatoms; *Pseudokeronopsis* sp. Brazil is also often contaminated by ochrophytes; *Durinskia baltica* is often contaminated with Chromulinaceae; *Colponema* transcriptomes are often contaminated by the excavate *Procryptobia* used to feed cultures. Finally, all proteins were screened again for the presence of the MIP domain using hmmsearch from the HMMER package v.3.3.2 (e-value threshold: 1e-3) and non-significant hits were further checked by BLASTP searches against NR, resulting in the exclusion of ten proteins. The final cleaned dataset contained a total of 7,541 proteins.

### Phylogenetic Inference and Identification of MIP Clades

The set of 7,541 full-length MIPs was subjected to alignment (MAFFT—auto; FFT-NS-2 iterative alignment), trimming (trimAL -gt 0.1), and IQ-TREE ML phylogenetic inference with BIC-selected best-fit LG+ F + Γ4 model of amino acid replacement. Branch support was assessed by transfer bootstrap expectation (Lemoine et al. 2018; as calculated by RAxML-NG v1.1.0 Lutteropp et al. 2020 using 1,000 standard bootstrapped trees calculated in IQ-TREE). To gain phylogenetic resolution in the study of MIP clades, we partitioned the large full-length dataset of 7,541 sequences into ten subsets of closely related sequences using the ML tree as a guide; each subset was then subjected to alignment, trimming, and independent ML inference with IQ-TREE as detailed above (support values in this case were UFBoot and SH-aLRT with 1,000 replicates each). The obtained ML trees were topologically indistinguishable from analogous analyses performed with RAxML-NG v.0.9.0 (AU test's *P* > 0.05; supplementary table S7, Supplementary Material online), with the exception of one dataset but topological differences pertained bacterial MIPs and not any of the six defined eukaryotic MIP paralog clades (Fig. 2j, supplementary fig. S11, Supplementary Material online). We further tested the reliability of our MAFFT alignments by implementing the “Heads-or-Tails” (HoT) approach (Landan and Graur 2007), which is based on the expectation that alignments should be independent of the orientation of the input sequences. Briefly, we used the HoT software (http://nsmn1.uh.edu/dgraur/scripts/HoT/) to generate forward (“head”) and reversed (“tail”) alignments for each of the ten data subsets (Fig. 2), which were subjected to ML tree inference (IQ-TREE under best-fit evolutionary models). For each of the ten data subsets, we performed topology tests to compare the head and tail tree topologies, both using the head and tail source alignments. The results showed no consistently significant differences between head and tail alignments (supplementary table S8, Supplementary Material online).

The ten smaller ML trees were used to define MIP clades (supplementary figs. S2 to S11, Supplementary Material online; trees available in Zenodo). All eukaryotic MIP clades described in the literature were searched in the ten subsets. Because a single AQP13 sequence remained in our dataset after decontamination, we added additional co-orthologs (Finn et al. 2014) to the subset of animal aquaglyceroporins and generated an updated tree (supplementary fig. S9, Supplementary Material online). The remaining diversity of eukaryotic MIP was then systematically defined by taxonomic clades mostly restricted to a single eukaryotic supergroup, total or partially (highly-supported clades with SH-aLRT >0.85). For each defined clade, we inferred the MRCA based on the taxonomic representation in our dataset (supplementary table S1, Supplementary Material online), which was then used as base for Fig. 1b.

To better identify deep orthology among prokaryote MIPs in our dataset, we downloaded bacterial and archaeal homologs defined by Finn et al. (2014) (their supplementary table S2) and Tesan et al. (2021) and inferred an independent ML tree together with our prokaryotic MIP homologs using IQ-TREE as detailed above (supplementary fig. S12, Supplementary Material online). Similarly, we downloaded available protein sequences used by Stavang et al. (2015) (their supplementary table S2), which were analyzed along our invertebrate MIPs as above (supplementary fig. S13, Supplementary Material online).

### Conservation of Amino Acid Residues

To further understand the evolutionary conservation of key residues, we extracted previously defined key amino acids, as identified after re-aligning each of the ten subsets with the *E. coli* AqpZ reference sequence (Uniprot acc. P60845). Residues include (i) the two NPA boxes (N63, P64, A65, N186, P187, A188), (ii) four ar/R selectivity filters (F43, H174, T183, R189), (iii) five Froger residues (Froger et al. 1998) (A103, S190, A194, F208, W209), and (iv) conserved residues identified by (Abascal et al. 2014) (E8, S58, G59, Q88, G91, N182, G212, G215). Residues were plotted with ETE3 (Huerta-Cepas et al. 2016). For the three identified deep MIP clades, the conservation of amino acids was inferred with ConSurf (Ashkenazy et al. 2016) and plotted onto the *Arabidopsis thaliana* PIP2-4 structure (Uniprot accession 6QIM) using custom alignments (MAFFT E-INS-i) and ML trees (IQ-TREE).

We explored conserved amino acids in each of the 127 phylogenetically-defined MIP paralog clades (supplementary figs. S2 to S13, Supplementary Material online) using three approaches. First, the 127 sequence subsets were re-aligned with MAFFT L-INS-I after adding the AqpZ sequence used as a reference to identify key amino acids conserved in ≥90% of the sequences (supplementary table S4, Supplementary Material online), for which we used the R v.4.0.2 package Biostrings (Pagès et al. 2020). Second, interactive alignment files were created displaying sequence conservation scores and LOGOs (supplementary data S1, Supplementary Material online) using the R package msaR (Rauscher and Charlop-Powers 2021). Third, for each MIP paralog clade, representative sequences were identified as those retaining every conserved amino acid (across ≥90% of the sequences) for that clade (supplementary table S5, Supplementary Material online).

### MIP Domain Identification

We searched the 7,541 sequences for the presence of MIP domains using hmmsearch and the Pfam MIP HMM profile (PF00230) with an e-value threshold of 1e-3. Sequences were clustered by the number of predicted MIP domains and their taxonomic distribution assessed by their occurrence across the 10 data subsets (supplementary figs. S2 to S13, Supplementary Material online).

### Statistical Analyses

The correlation between the initial number of EukProt protein sets per eukaryotic supergroup and the number of MIP homologs in the final dataset was tested with Pearson's correlation in *R*.

## Supplementary Material

evae164_Supplementary_Data

## Data Availability

Further information and resource request should be directed to and will be fulfilled by Iker Irisarri (Irisarri.iker@gmail.com). Supplementary data, Supplementary Material online, multiple sequence alignments, trees, conserved amino acid reports, and R scripts are available in Zenodo (DOI: 10.5281/zenodo.12744918).
