# Supplementary material for: Early Diversification of Membrane Intrinsic Proteins (MIPs) in Eukaryotes
Source: Genome Biol Evol. 2024 Jul 26;16(8):evae164. doi: 10.1093/gbe/evae164 (PMC11316224; doi:10.1093/gbe/evae164)
Supplement: evae164_Supplementary_Data [file evae164_supplementary_data.zip › Suppl_Data_1/Suppl_Data_1/Amoebozoa_I_Eumycetozoa_S4.linsi.html]

msaR
