# Supplementary material for: Early Diversification of Membrane Intrinsic Proteins (MIPs) in Eukaryotes
Source: Genome Biol Evol. 2024 Jul 26;16(8):evae164. doi: 10.1093/gbe/evae164 (PMC11316224; doi:10.1093/gbe/evae164)
Supplement: evae164_Supplementary_Data [file Supplementary Figures.pdf › Supplementary Figures.pdf]

Supplementary Data, Tables, and Figures

Table S1. MIP clades as defined here based on phylogenetic trees from the ten data subsets and their reconstructed evolutionary origin (most recent common ancestor).

Table S2. Taxonomic distribution of selected MIP clades across eukaryotes with sequenced genomes in our dataset (it corresponds to Figure S15).

Table S3. Distribution of MIP homologs in our dataset for unicellular eukaryotes across here-defined paralogy clades, with emphasis on 'MIP deep clades' (MDC).

Table S4. Conserved amino acid residues for 127 defined MIP paralogy clades. Residues conserved in ≥90% of the sequences are shown, indicating the corresponding coordinate and amino acid from the *E. coli* AqpZ sequence, as well as the amino acid conserved in the paralogy clade.

Table S5. Representative sequences for 127 defined MIP paralog clades, defined as those that retain all amino acids that are conserved in ≥90% of the sequences.

Table S6. Taxonomic distribution of 32 Archaea MIP homologs from our dataset.

Table S7. Comparison of IQ-TREE and RAxML topologies for the ten data subsets (Fig. 2a-j) using topology tests.

Table S8. Comparison of IQ-TREE topologies derived from "head" and "tail" alignments for the ten data subsets (Fig. 2a-j) using topology tests; tests are repeated using either the "head" or "tail" alignments.

Figure S1. Maximum likelihood tree of 7,541 MIP homologs (IQ-TREE, midpoint rooting). Branch support is indicated as transfer bootstrap expectation values. The ten data subsets that are independently analyzed are indicated in the tree.

Figure S2. Maximum likelihood tree of alignment subset in Fig. 2a (IQ-TREE, midpoint rooting). Branch support is indicated as SH-aLRT and UFBoot, respectively. Taxa are colored according to taxonomy and MIP clades mentioned in the text are highlighted. Key amino acid residues are shown: NPA boxes, ar/R selectivity filters, Froger residues (P1-5), and conserved residues identified by Abascal et al. (2014) (asterisks).

Figure S3. Maximum likelihood tree of alignment subset in Fig. 2b (IQ-TREE, midpoint rooting). Branch support is indicated as SH-aLRT and UFBoot, respectively. Taxa are colored according to taxonomy and MIP clades mentioned in the text are highlighted. Key amino acid residues are shown: NPA boxes, ar/R selectivity filters, Froger residues (P1-5), and conserved residues identified by Abascal et al. (2014) (asterisks).

Figure S4. Maximum likelihood tree of alignment subset in Fig. 2c (IQ-TREE, midpoint rooting). Branch support is indicated as SH-aLRT and UFBoot, respectively. Taxa are colored according to taxonomy and MIP clades mentioned in the text are highlighted. Key amino acid residues are shown: NPA boxes, ar/R selectivity filters, Froger residues (P1-5), and conserved residues identified by Abascal et al. (2014) (asterisks).

Figure S5. Maximum likelihood tree of alignment subset in Fig. 2d (IQ-TREE, midpoint rooting). Branch support is indicated as SH-aLRT and UFBoot, respectively. Taxa are colored according to taxonomy and MIP clades mentioned in the text are highlighted. Key amino acid residues are shown: NPA boxes, ar/R selectivity filters, Froger residues (P1-5), and conserved residues identified by Abascal et al. (2014) (asterisks).

Figure S6. Maximum likelihood tree of alignment subset in Fig. 2e (IQ-TREE, midpoint rooting). Branch support is indicated as SH-aLRT and UFBoot, respectively. Taxa are colored according to taxonomy and MIP clades mentioned in the text are highlighted. Key amino acid residues are shown: NPA boxes, ar/R selectivity filters, Froger residues (P1-5), and conserved residues identified by Abascal et al. (2014) (asterisks).

Figure S7. Maximum likelihood tree of alignment subset in Fig. 2f (IQ-TREE, midpoint rooting). Branch support is indicated as SH-aLRT and UFBoot, respectively. Taxa are colored according to taxonomy and MIP clades mentioned in the text are highlighted. Key amino acid residues are shown: NPA boxes, ar/R selectivity filters, Froger residues (P1-5), and conserved residues identified by Abascal et al. (2014) (asterisks).

Figure S8. Maximum likelihood tree of alignment subset in Fig. 2g (IQ-TREE, midpoint rooting). Branch support is indicated as SH-aLRT and UFBoot, respectively. Taxa are colored according to taxonomy and MIP clades mentioned in the text are highlighted. Key amino acid residues are shown: NPA boxes, ar/R selectivity filters, Froger residues (P1-5), and conserved residues identified by Abascal et al. (2014) (asterisks).

Figure S9. Maximum likelihood tree of alignment subset in Fig. 2h (IQ-TREE, midpoint rooting). Branch support is indicated as SH-aLRT and UFBoot, respectively. Taxa are colored according to taxonomy and MIP clades mentioned in the text are highlighted. Key amino acid residues are shown: NPA boxes, ar/R selectivity filters, Froger residues (P1-5), and conserved residues identified by Abascal et al. (2014) (asterisks).

Figure S10. Maximum likelihood tree of alignment subset in Fig. 2i (IQ-TREE, midpoint rooting). Branch support is indicated as SH-aLRT and UFBoot, respectively. Taxa are colored according to taxonomy and MIP clades mentioned in the text are highlighted. Key amino acid residues are shown: NPA boxes, ar/R selectivity filters, Froger residues (P1-5), and conserved residues identified by Abascal et al. (2014) (asterisks).

Figure S11. Maximum likelihood tree of alignment subset in Fig. 2j (IQ-TREE, midpoint rooting). Branch support is indicated as SH-aLRT and UFBoot, respectively. Taxa are colored according to taxonomy and MIP clades mentioned in the text are highlighted. Key amino acid residues are shown: NPA boxes, ar/R selectivity filters, Froger residues (P1-5), and conserved residues identified by Abascal et al. (2014) (asterisks).

Figure S12. Maximum likelihood tree of prokaryote MIPs with representative archaeal and bacterial homologs of AqpZ, AqpN, AqpM, AqpX, and GlpF from Finn et al. (2014) and Tesan et al. (2021) (IQ-TREE, midpoint rooting). Branch support is indicated as SH-aLRT and UFBoot, respectively.

Figure S13. Maximum likelihood tree of invertebrate MIPs with representative arthropod MIPs from Stavang et al. (2015) (IQ-TREE, midpoint rooting). Branch support is indicated as SH-aLRT and UFBoot, respectively.

Figure S14. Correlation plot between the number of MIP homologs in the final dataset per eukaryotic supergroup and the number of protein sets (from genomes, transcriptomes and EST collections) in EukProt v. 2 for the different eukaryotic "supergroups". Pearson's correlation coefficient and the associated p-value are shown.

Figure S15. Taxonomic distribution of selected MIP clades across eukaryotes with sequenced genomes in our dataset. Bubble size corresponds to the number of MIP paralogs per group annotated in the genomes, and aquaporins (blue) and aquaglyceroporins (yellow) are differentiated (corresponding data in Table S2).

Supplementary Data 1. Alignments for each of the 127 defined MIP paralogy clades showing sequence conservation as both scores and LOGOs (html format).

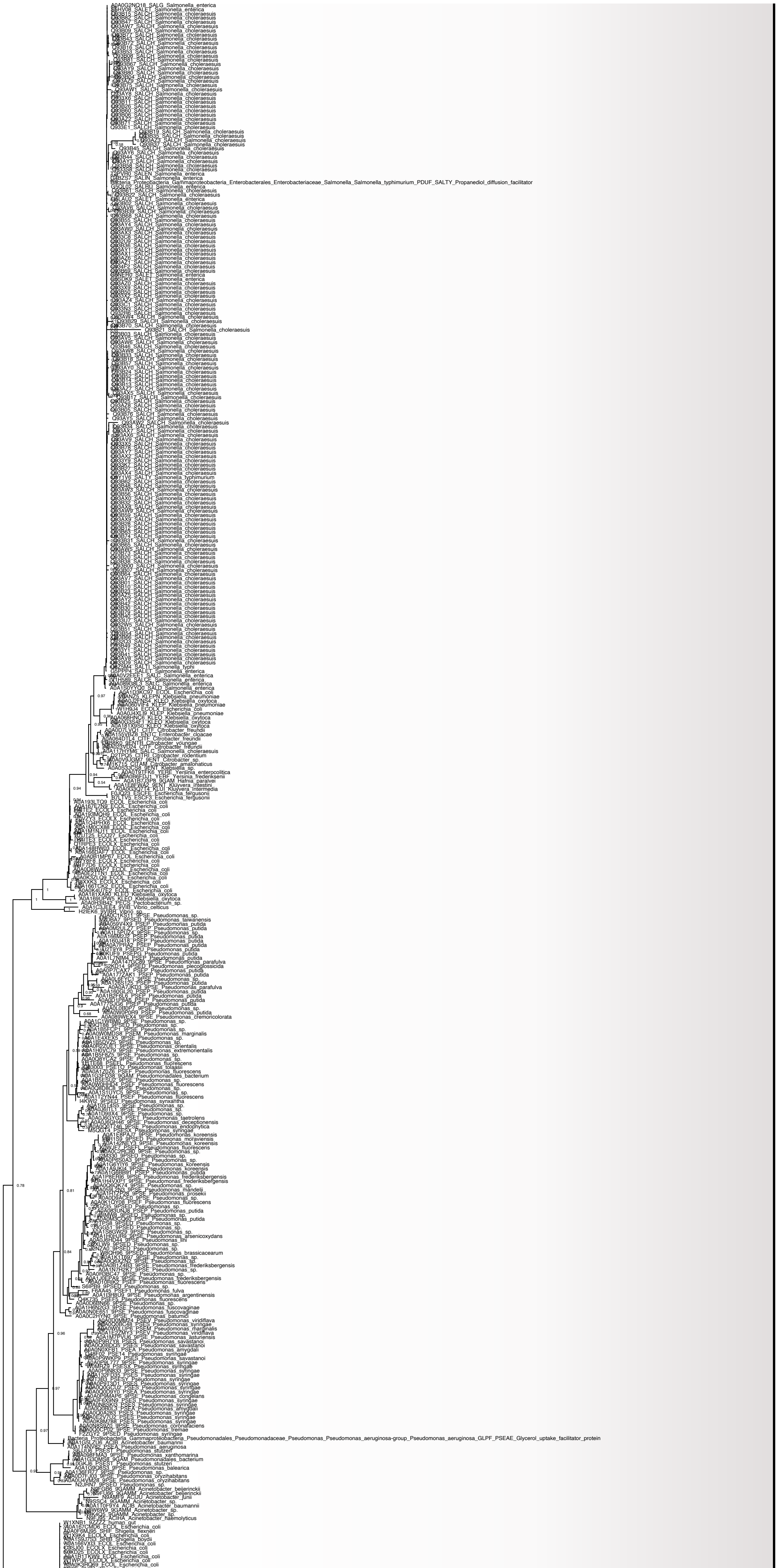

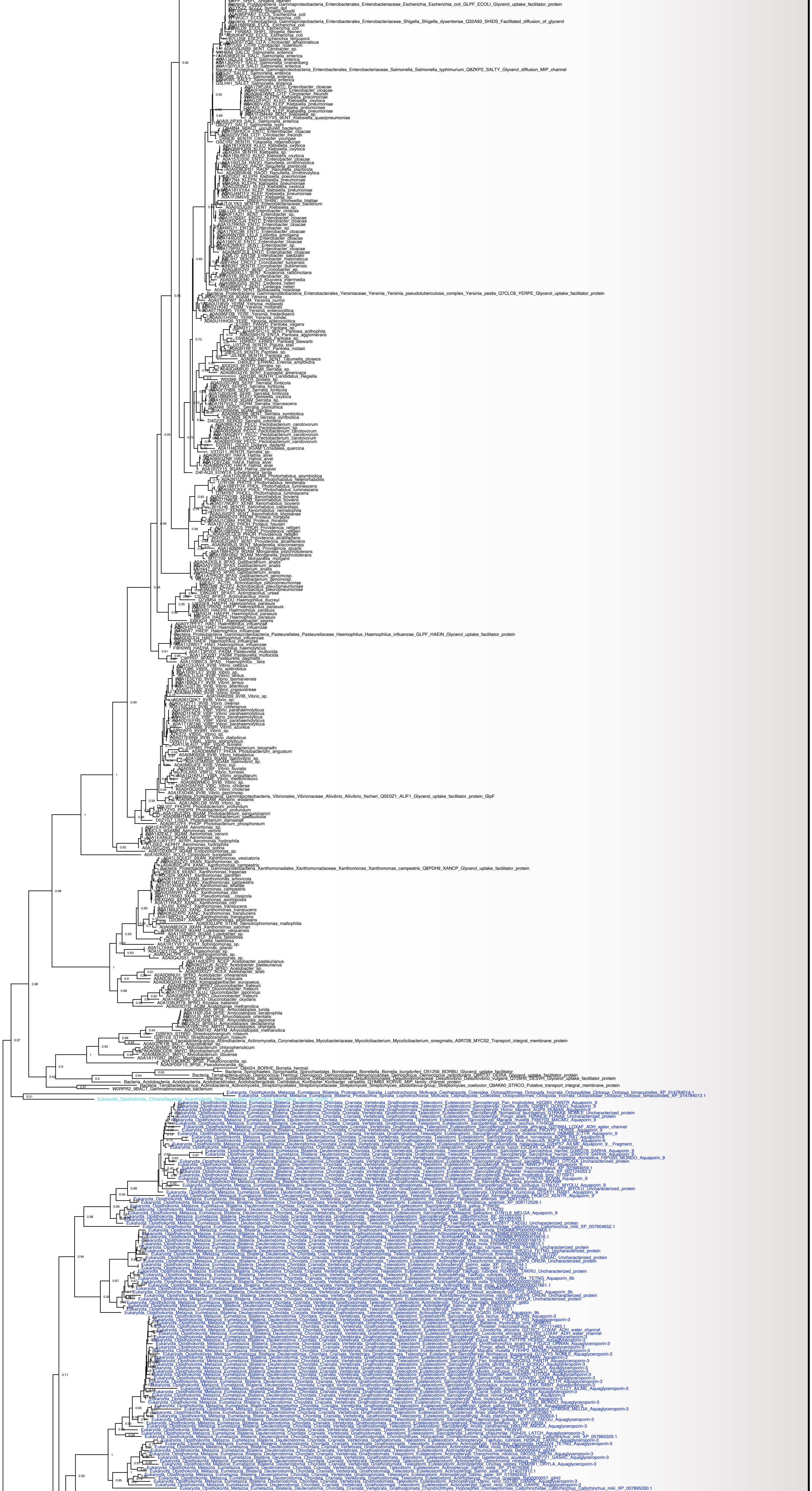

Fig. 2h

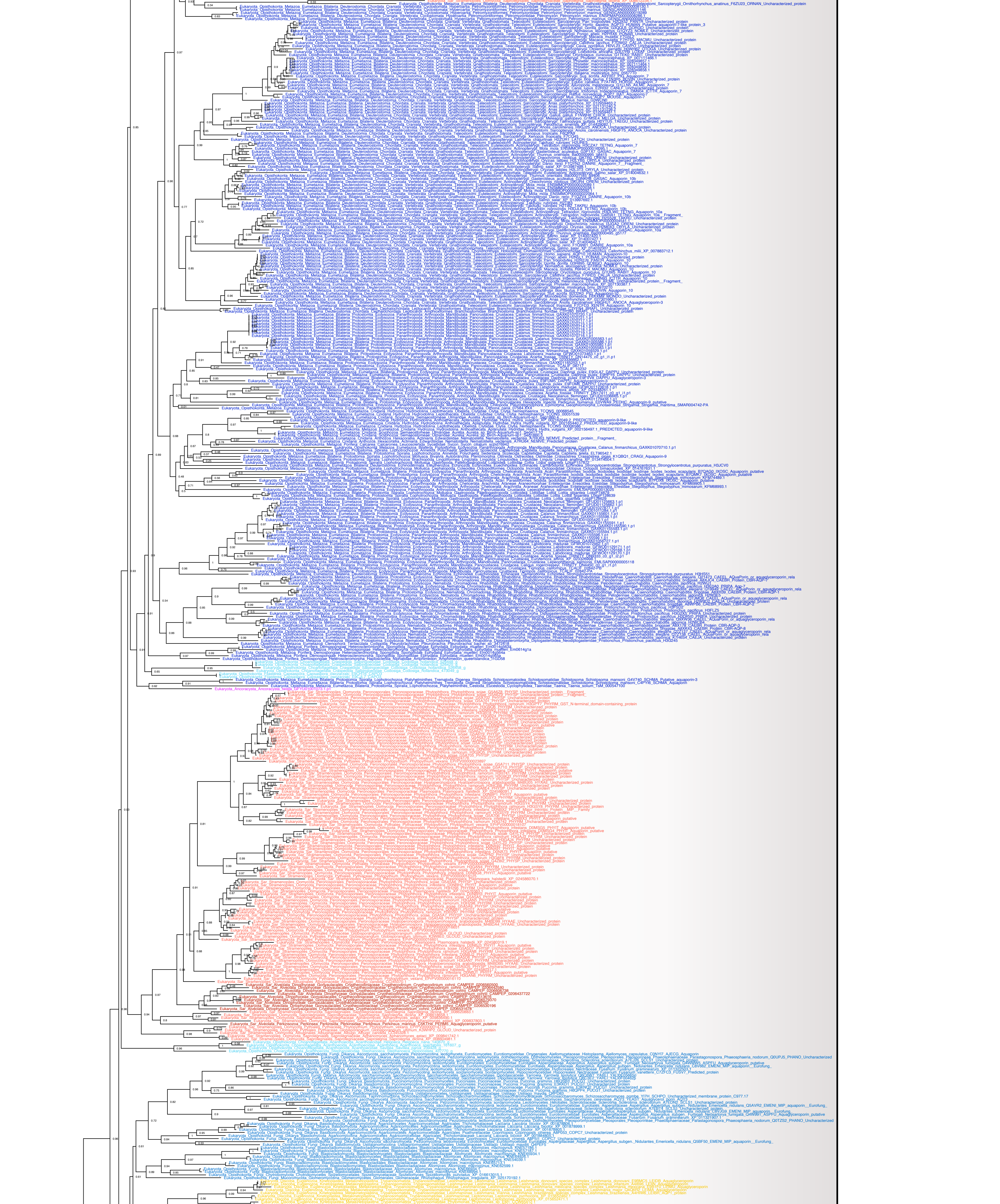

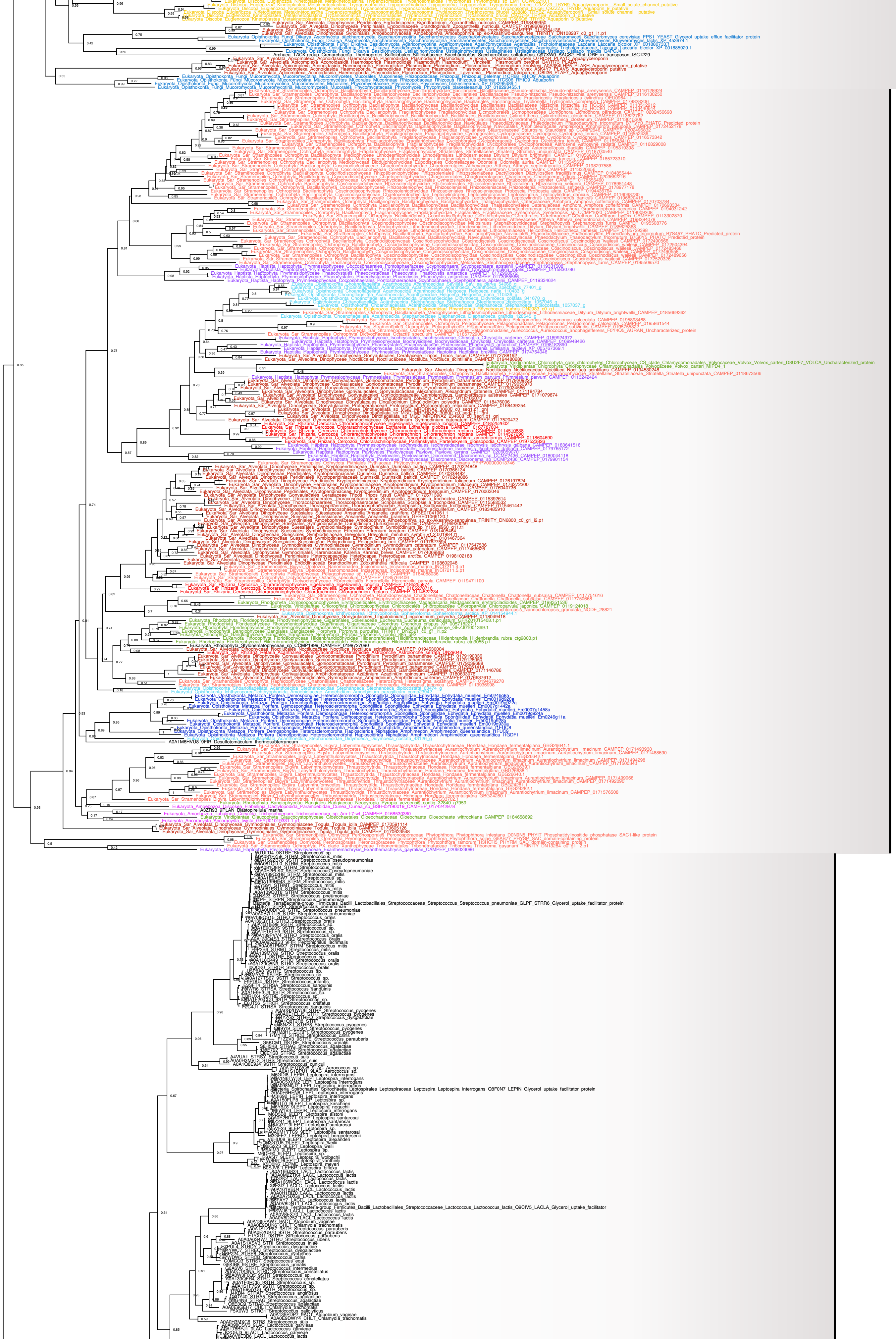

Fig. 2i

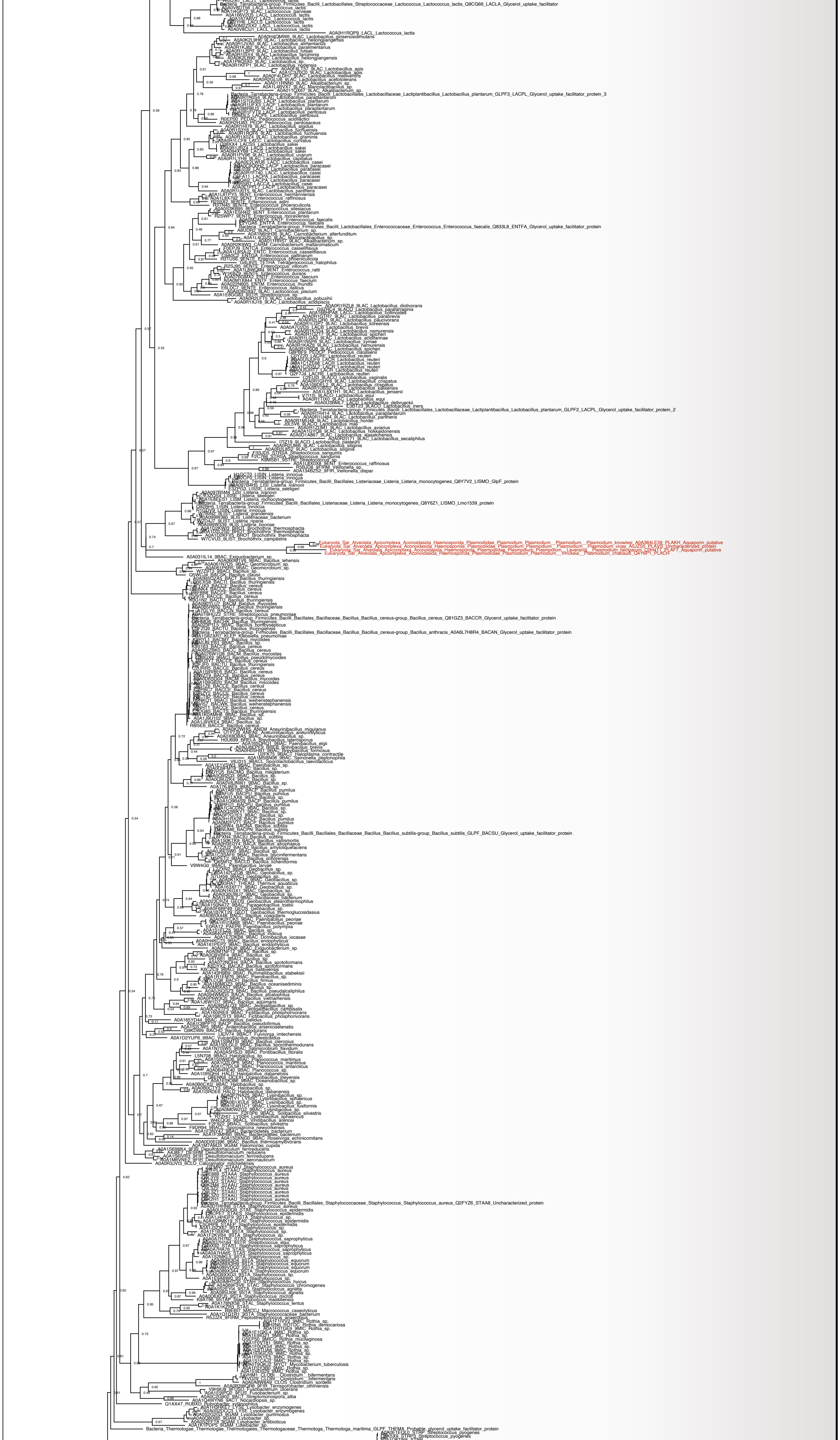

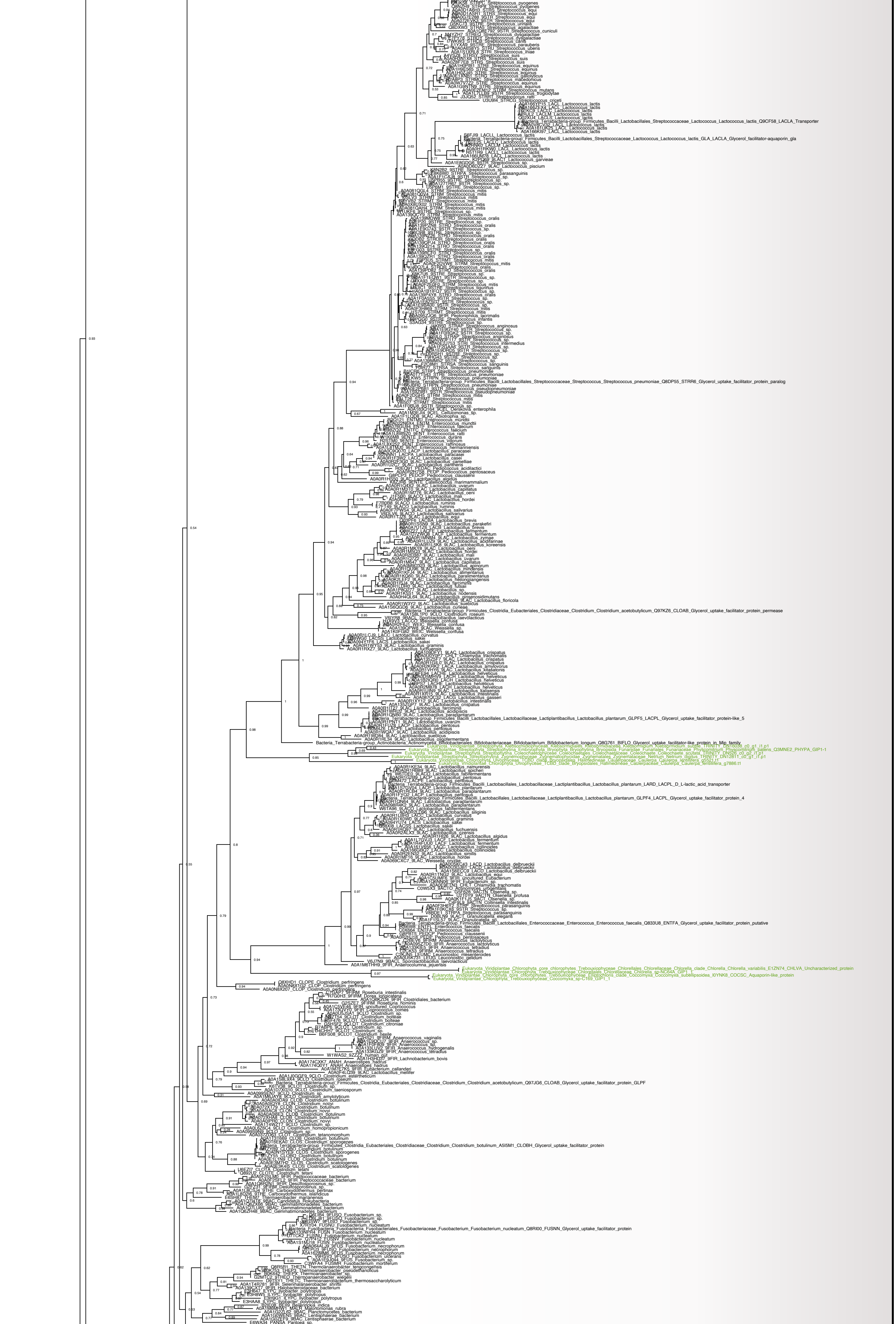

Fig. 2j

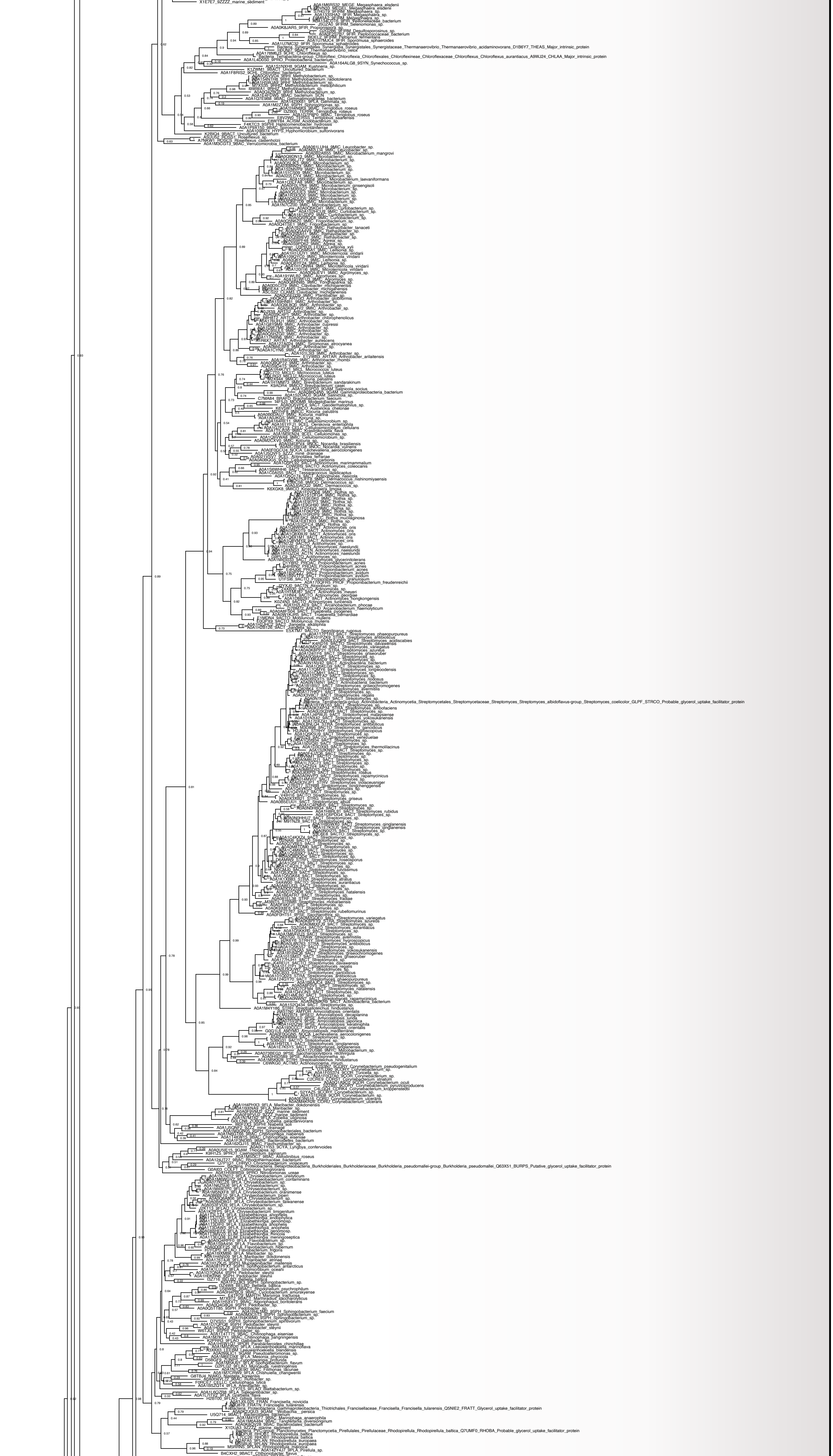

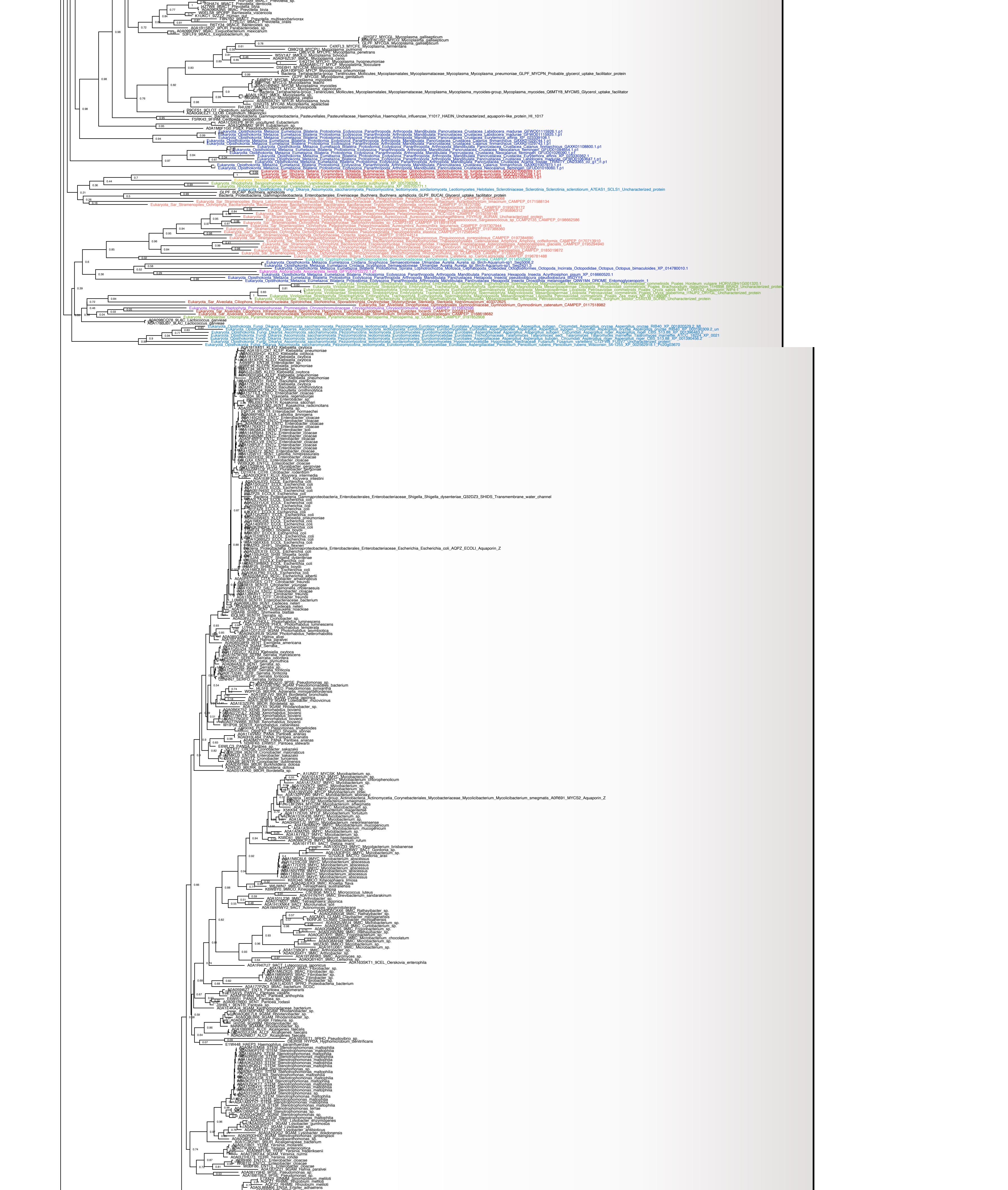

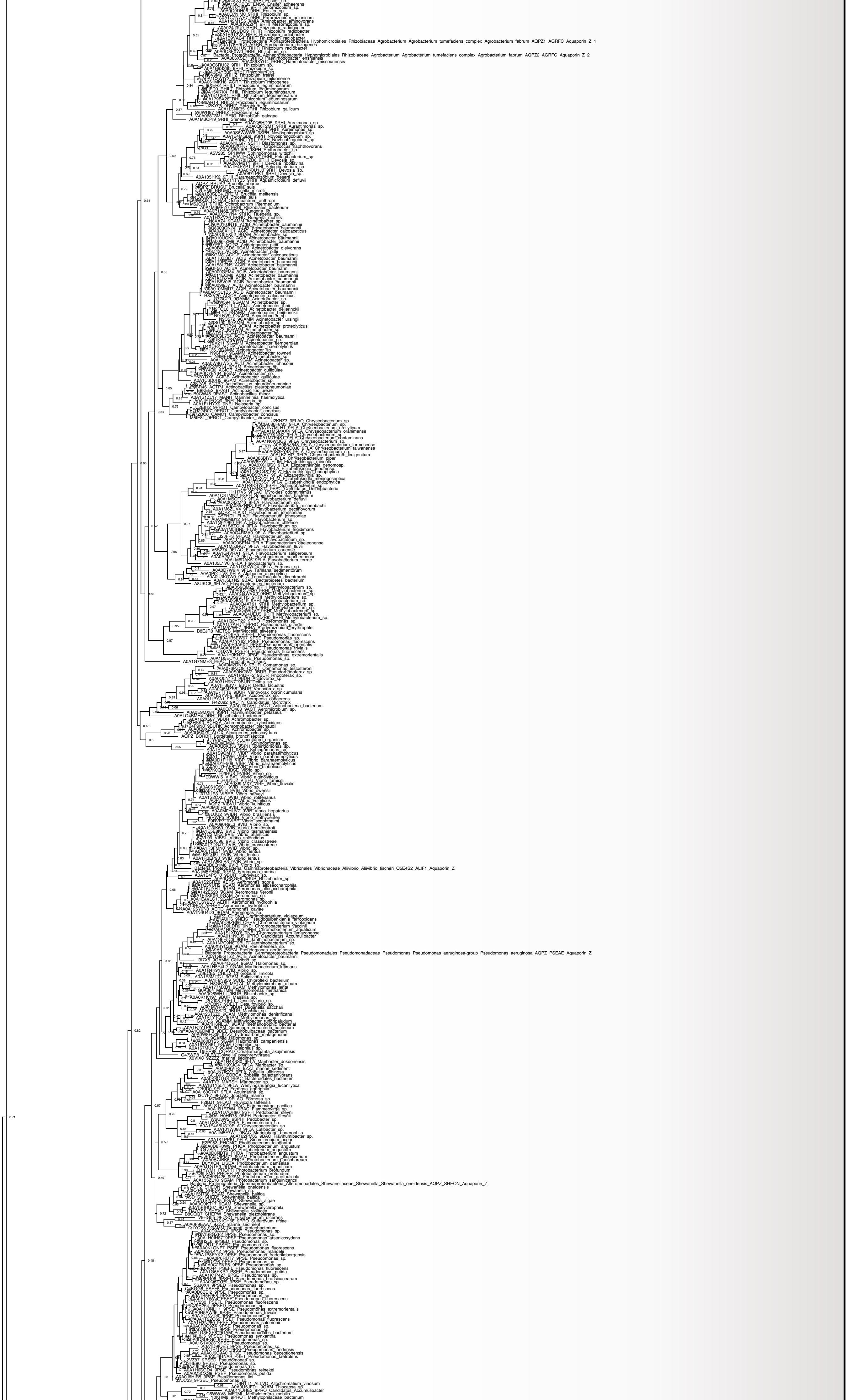

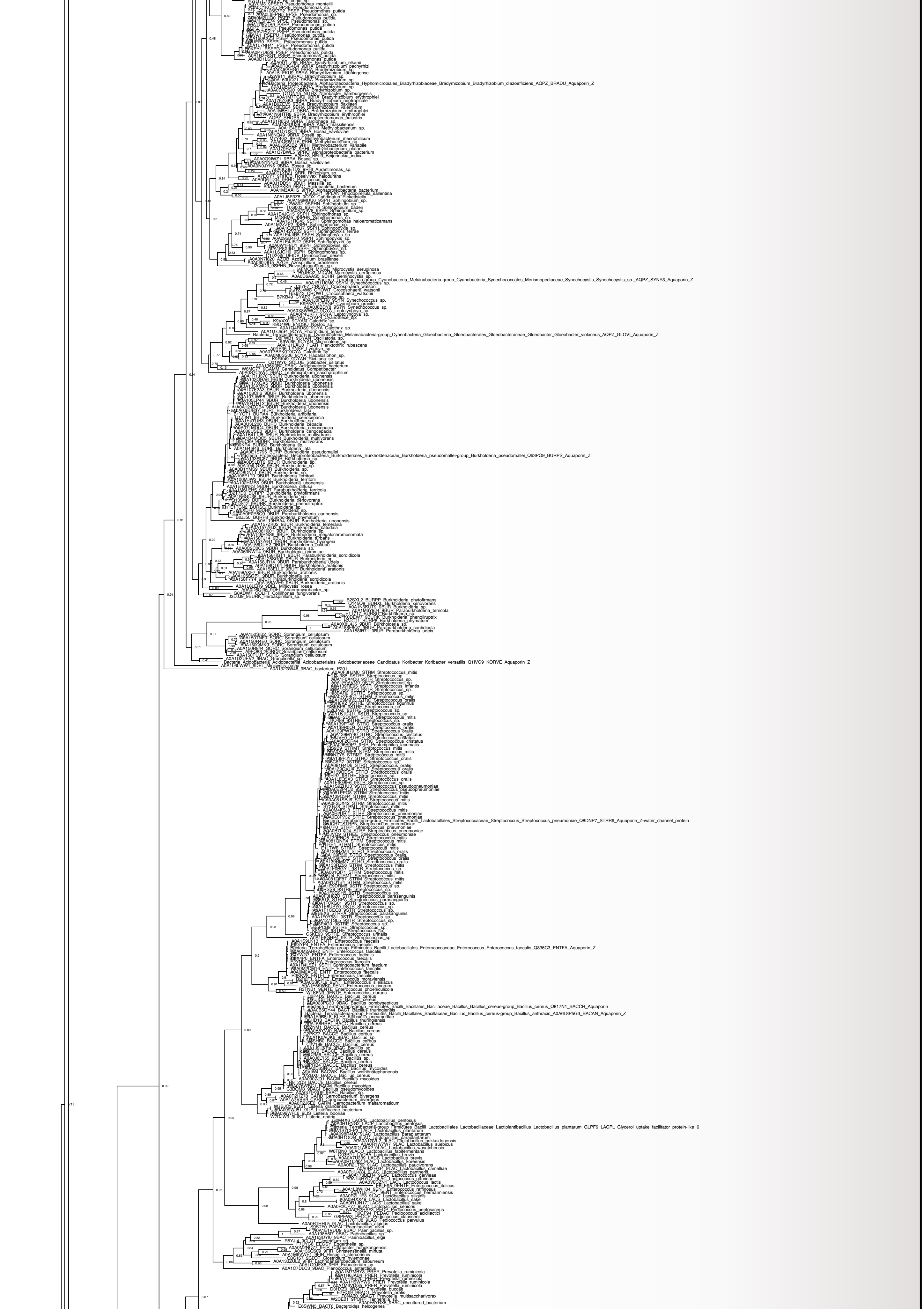

Fig. 2g

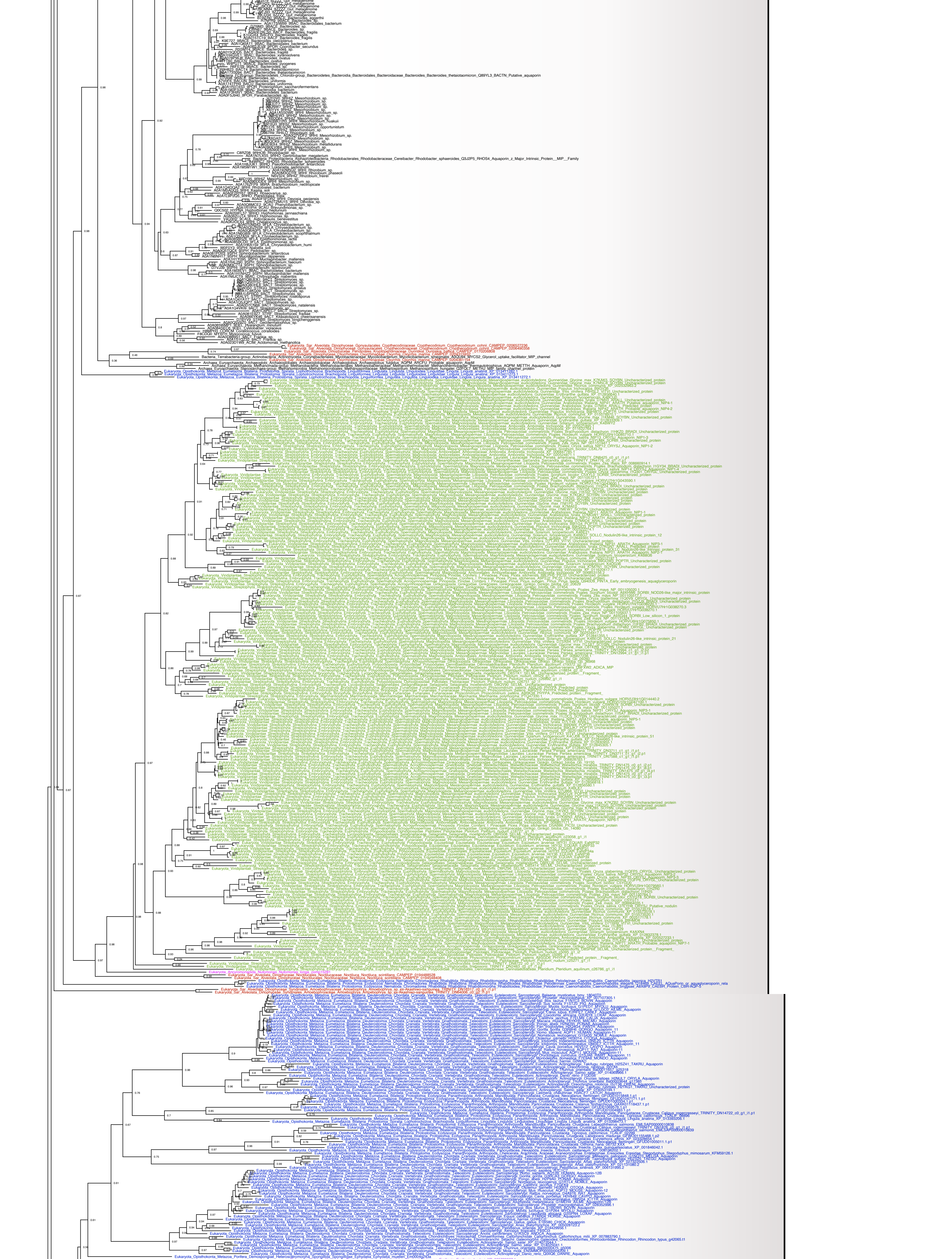

Fig. 2f

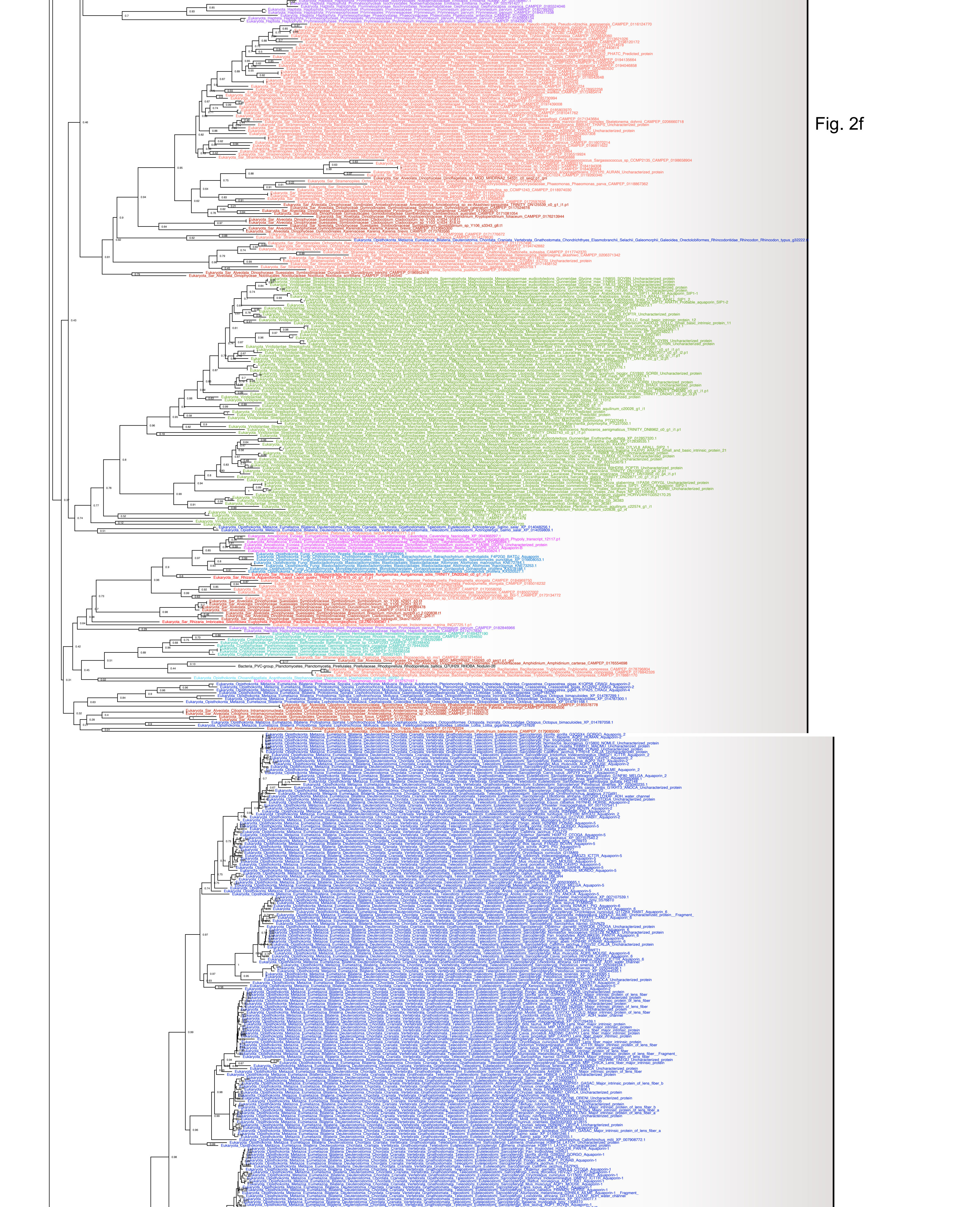

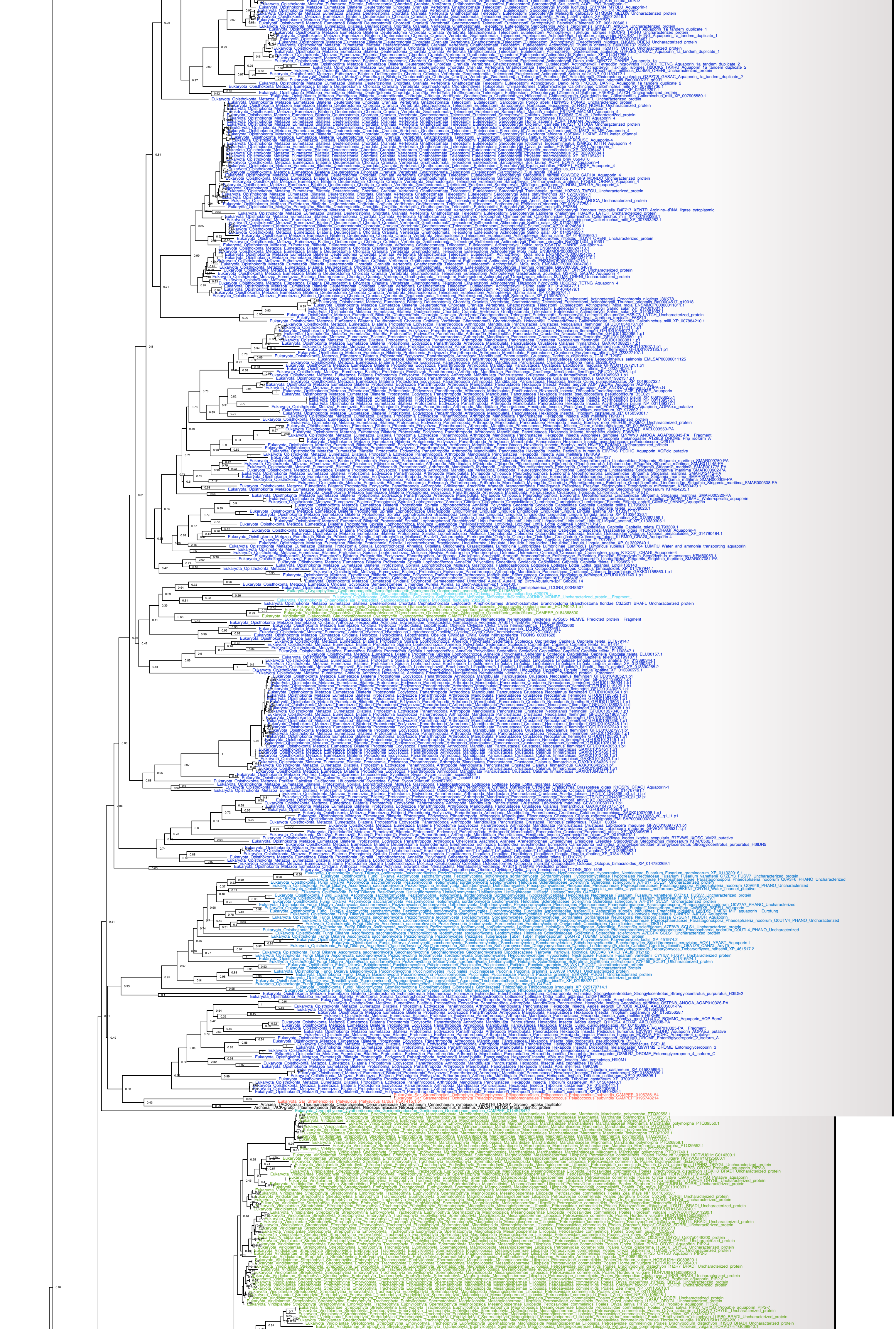

Fig. 2a

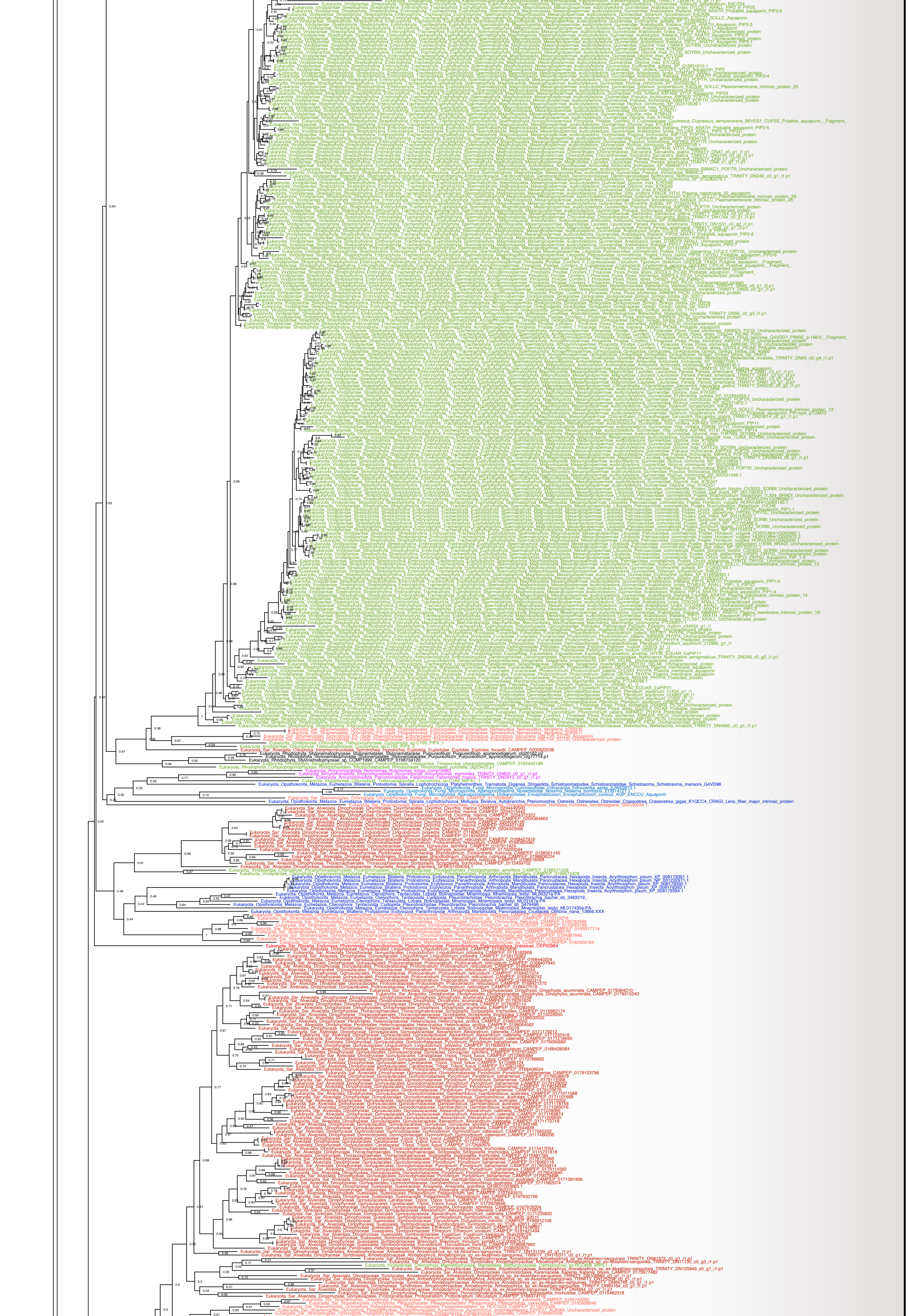

Fig. 2b

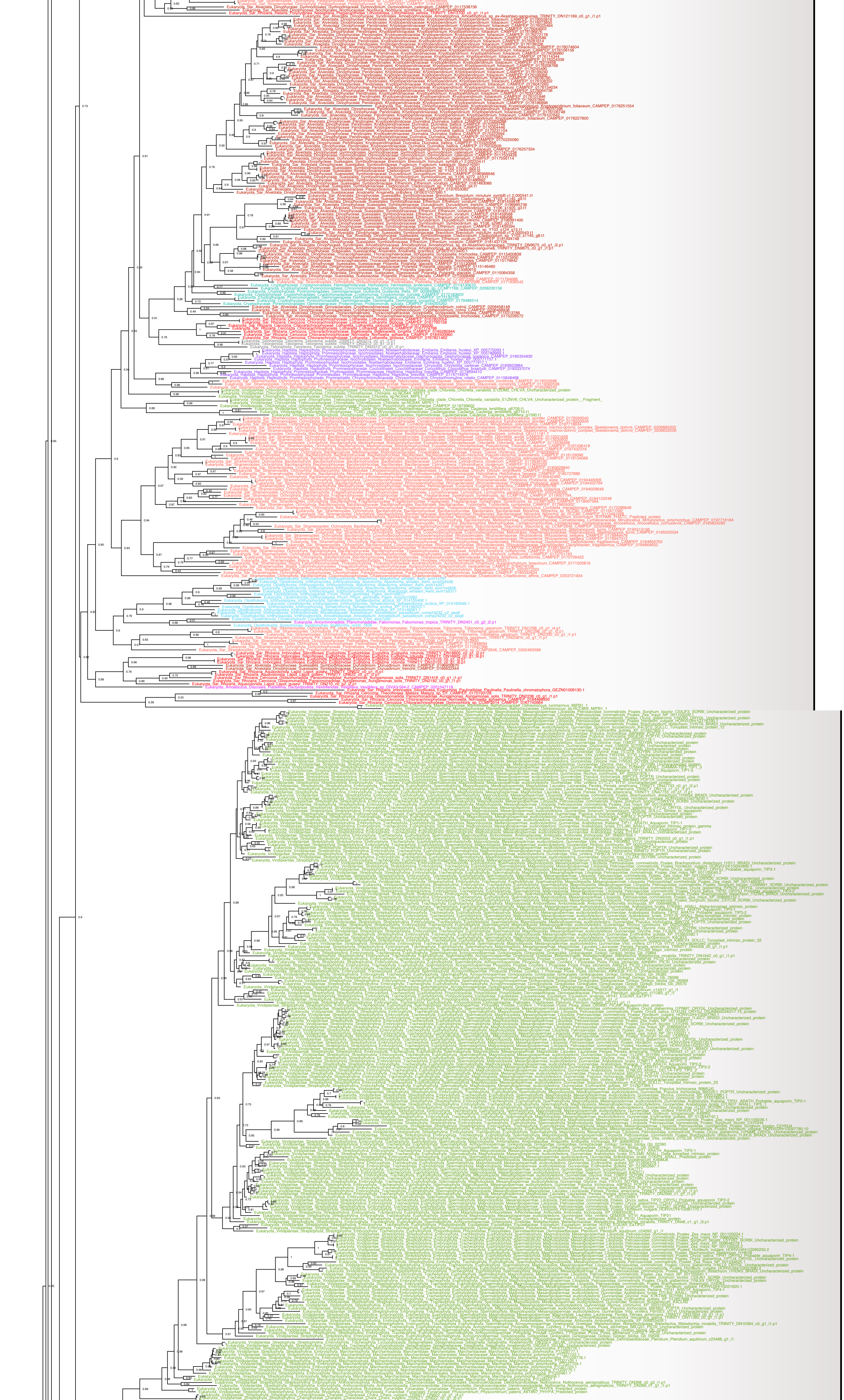

Fig. 2c

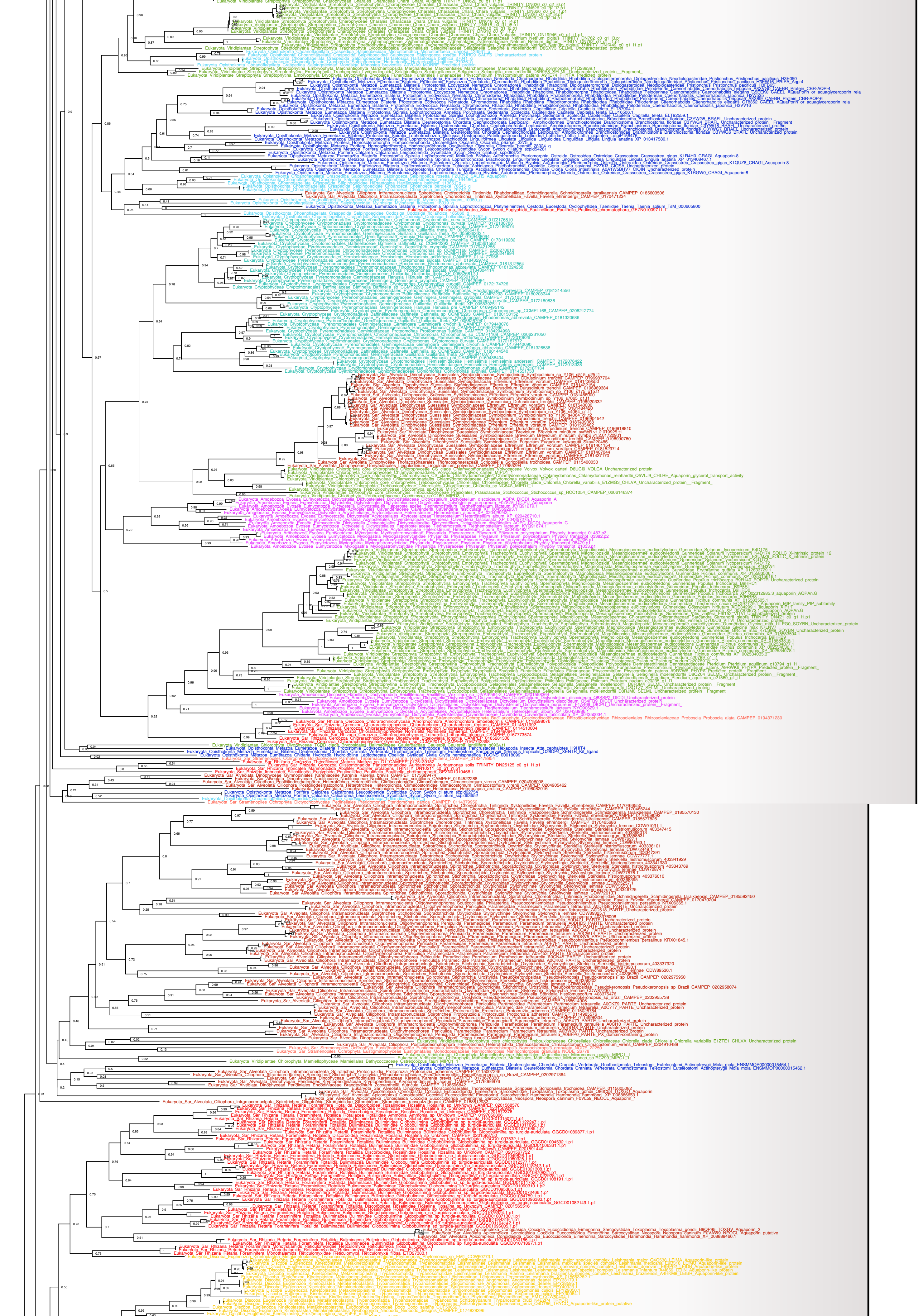

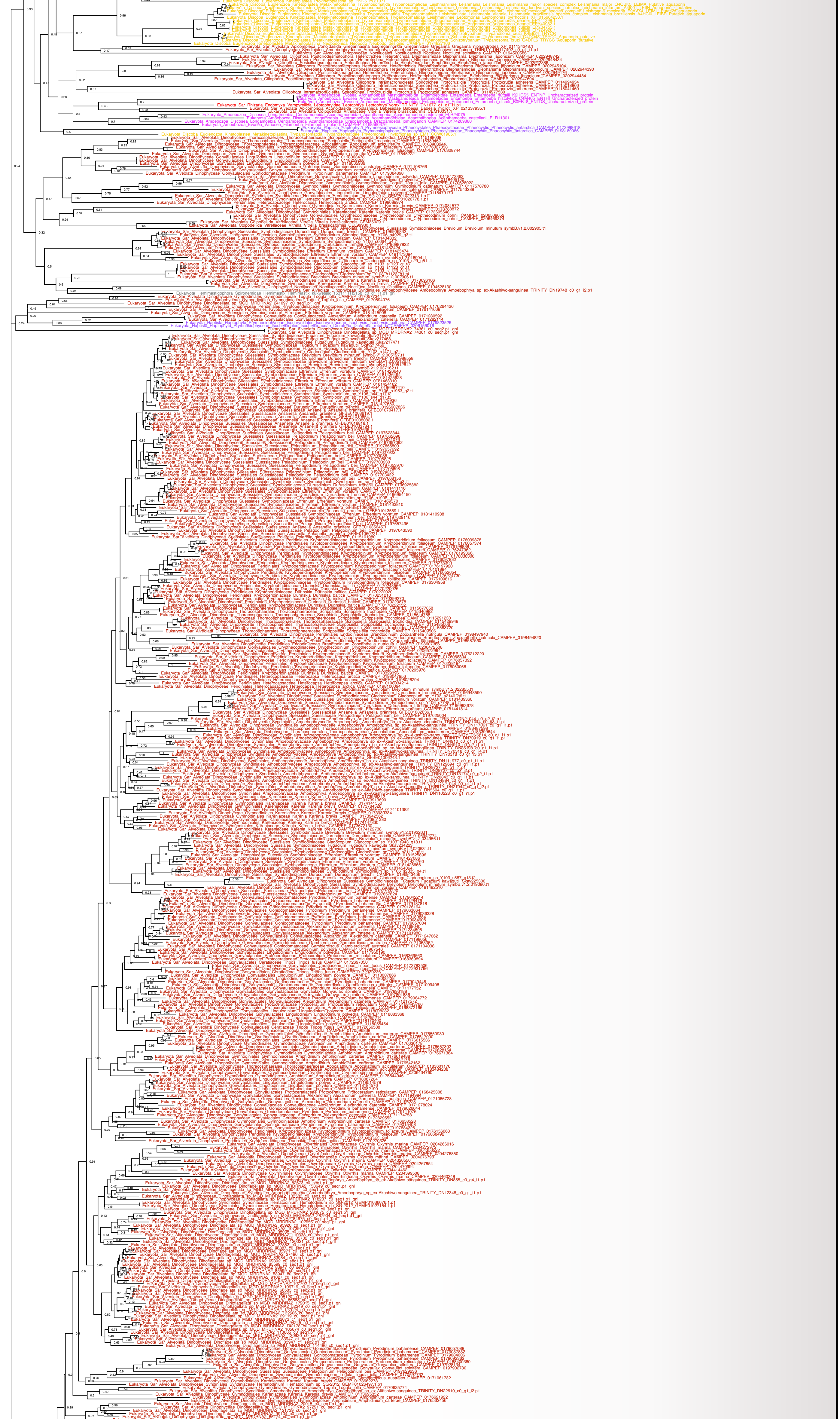

Fig. 2d

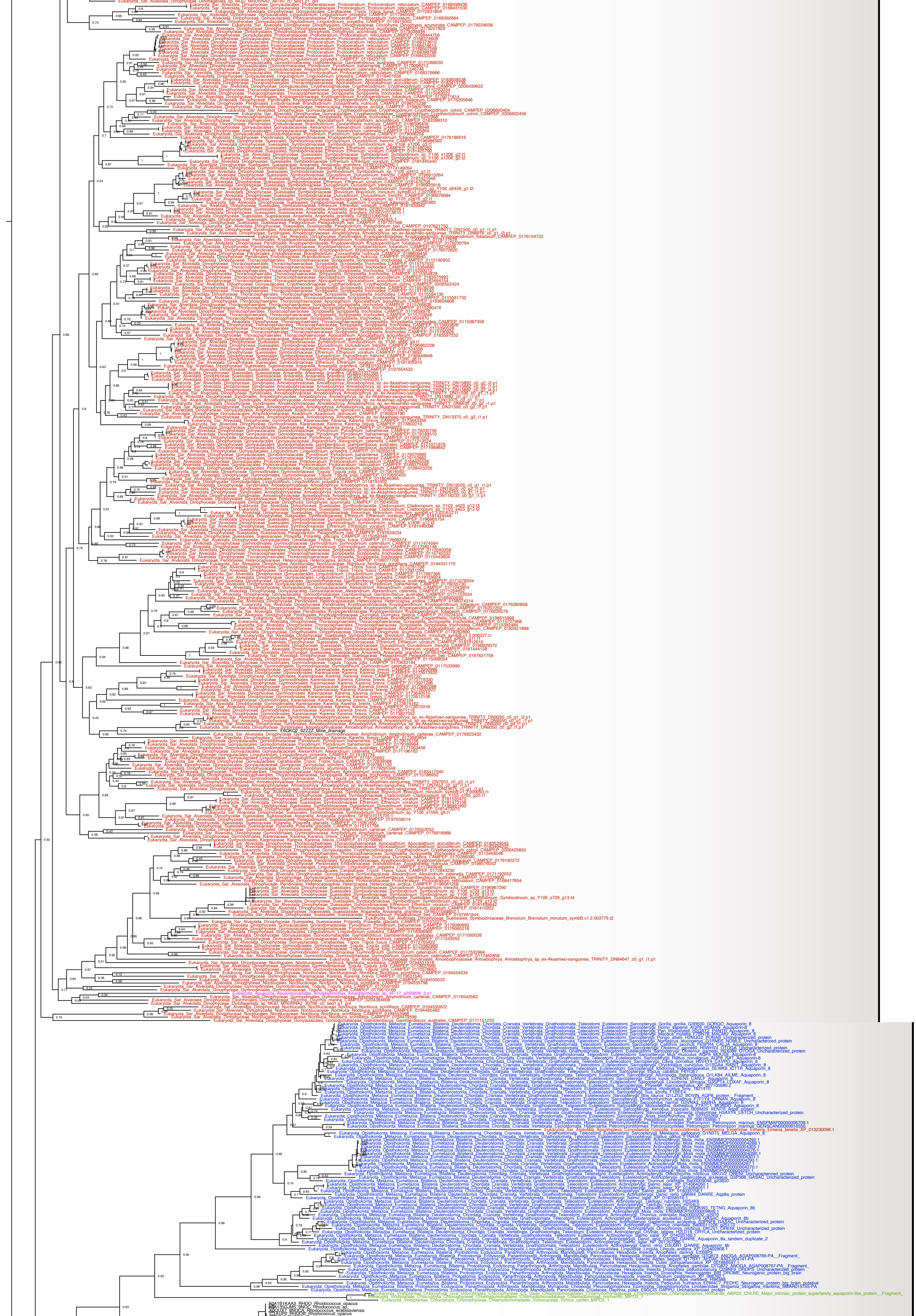

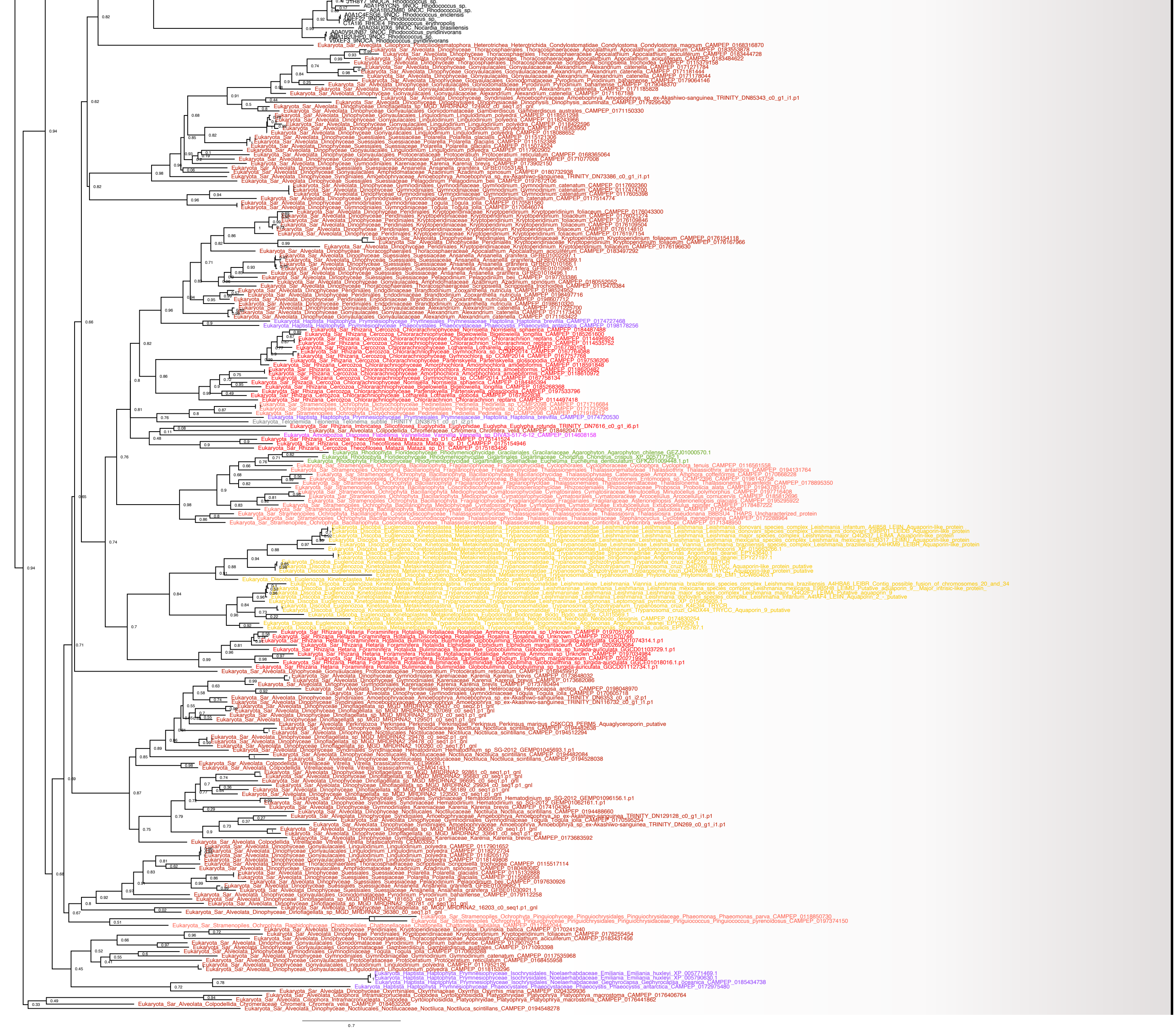

Fig. 2e

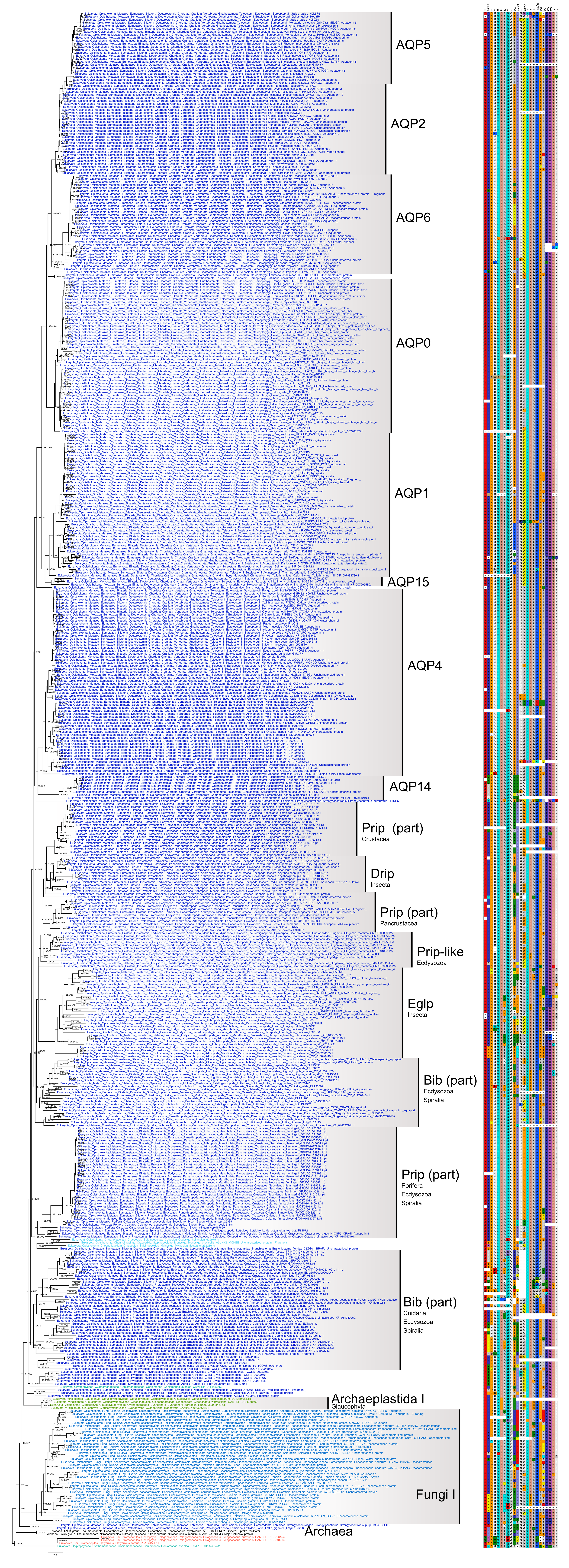
AQP5
AQP2
AQP6
AQP0
AQP1
AQP15
AQP4
AQP14
Prip (part)
Crustacea
Drip
Insecta
Prip (part)
Pancrustacea
Prip-like
Ecdysozoa
Eglp
Insecta
Bib (part)
Ecdysozoa
Spiralia
Prip (part)
Porifera
Ecdysozoa
Spiralia
Bib (part)
Cnidaria
Ecdysozoa
Spiralia
Archaeplastida I
Glaucophyta
Fungi I
Archaea

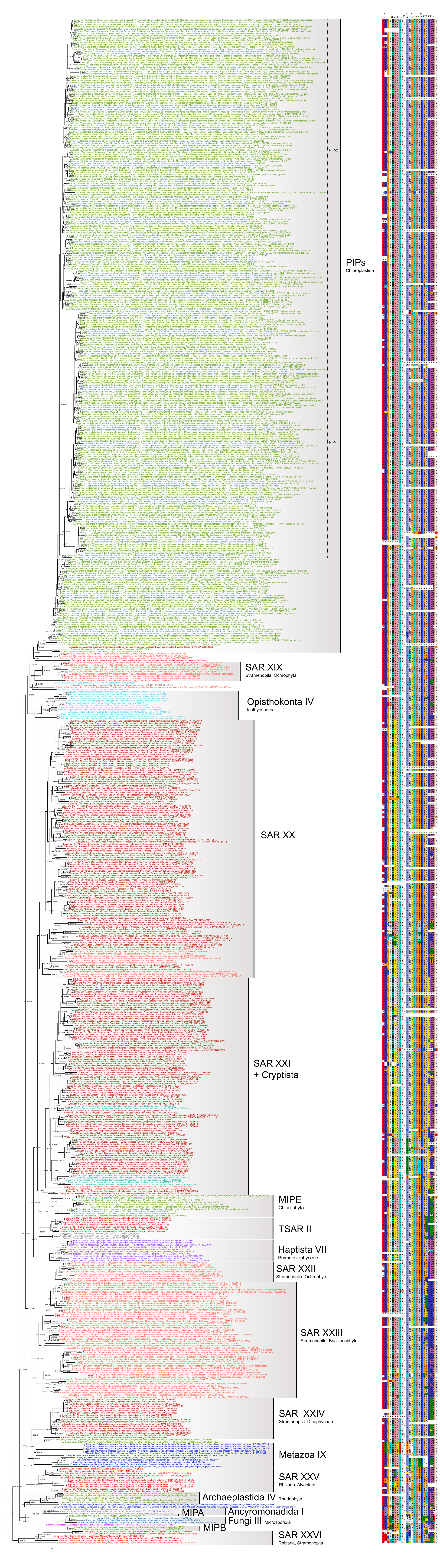
PIP-2
PIPs
Chloroplastida
PIP-1
SAR XIX
Stramenopila: Ochrophyta
Opisthokonta IV
Ichthyosporea
SAR XX
SAR XXI
+ Cryptista
MIPE
Chlorophyta
TSAR II
Haptista VII
Prymnesiophyceae
SAR XXII
Stramenopila: Ochrophyta
SAR XXIII
Stramenopila: Bacillariophyta
SAR XXIV
Stramenopila: Dinophyceae
Metazoa IX
SAR XXV
Rhizaria, Alveolata
Archaeplastida IV
Rhodophyta
MIPA
Ancyromonadida I
Fungi III
Microsporidia
MIPB
SAR XXVI
Rhizaria, Stramenopila

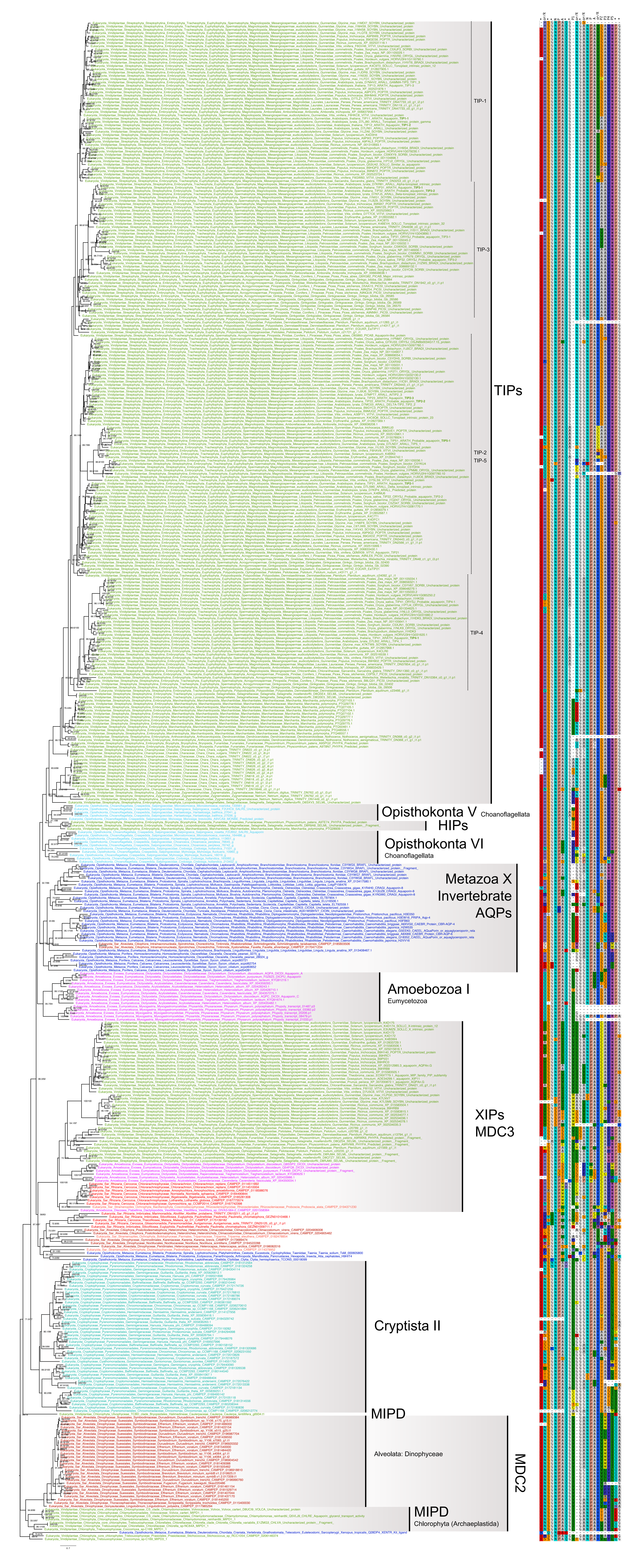
TIP-1
TIP-3
TIPs
TIP-2
TIP-5
TIP-4
Opisthokonta V
Choanoflagellata
HIPs
Opisthokonta VI
Choanoflagellata
Metazoa X
Invertebrate
AQPs
Amoebozoa I
Eumycetozoa
XIPs
MDC3
Cryptista II
MIPD
Alveolata: Dinophyceae
MDC2
MIPD
Chlorophyta (Archaeplastida)

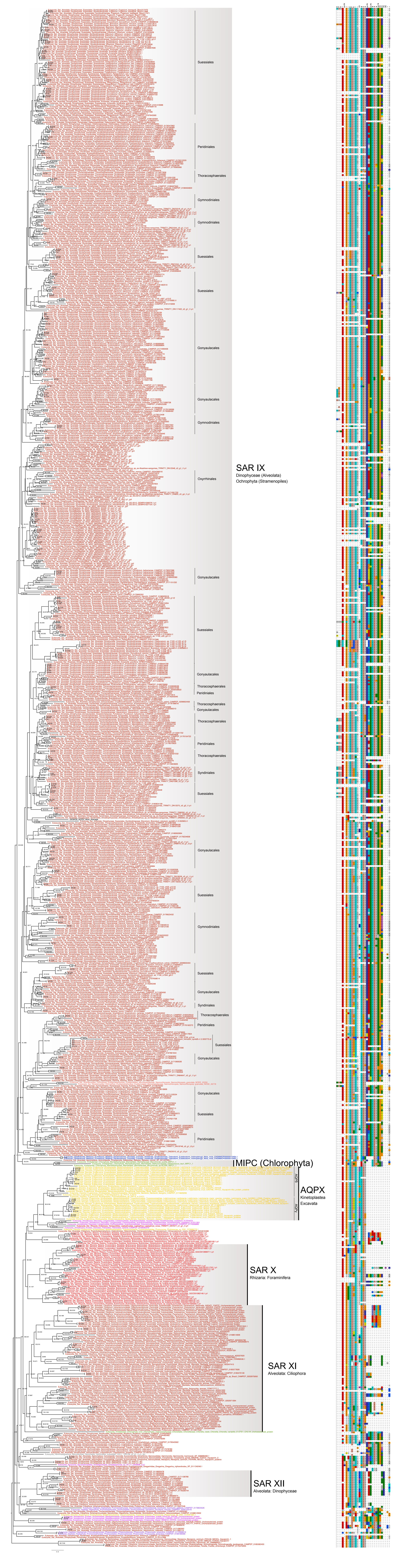

Suessiales
Peridiniales
Thoracosphaerales
Gymnodiniales
Gymnodiniales
Suessiales
Suessiales
Gonyaulacales
Gonyaulacales
Gymnodiniales
SAR IX
Dinophyceae (Alveolata)
Ochrophyta (Stramenopiles)
Oxyrrhinales
Gonyaulacales
Suessiales
Gonyaulacales
Thoracosphaerales
Peridiniales
Thoracosphaerales
Gonyaulacales
Thoracosphaerales
Peridiniales
Thoracosphaerales
Syndiniales
Suessiales
Gonyaulacales
Suessiales
Gymnodiniales
Suessiales
Gonyaulacales
Syndiniales
Thoracosphaerales
Peridiniales
Suessiales
Gonyaulacales
Gonyaulacales
Suessiales
Peridiniales
IMIPC (Chlorophyta)
AQPX
Kinetoplastea
Excavata
SAR X
Rhizaria: Foraminifera
SAR XI
Alveolata: Ciliophora
SAR XII
Alveolata: Dinophyceae

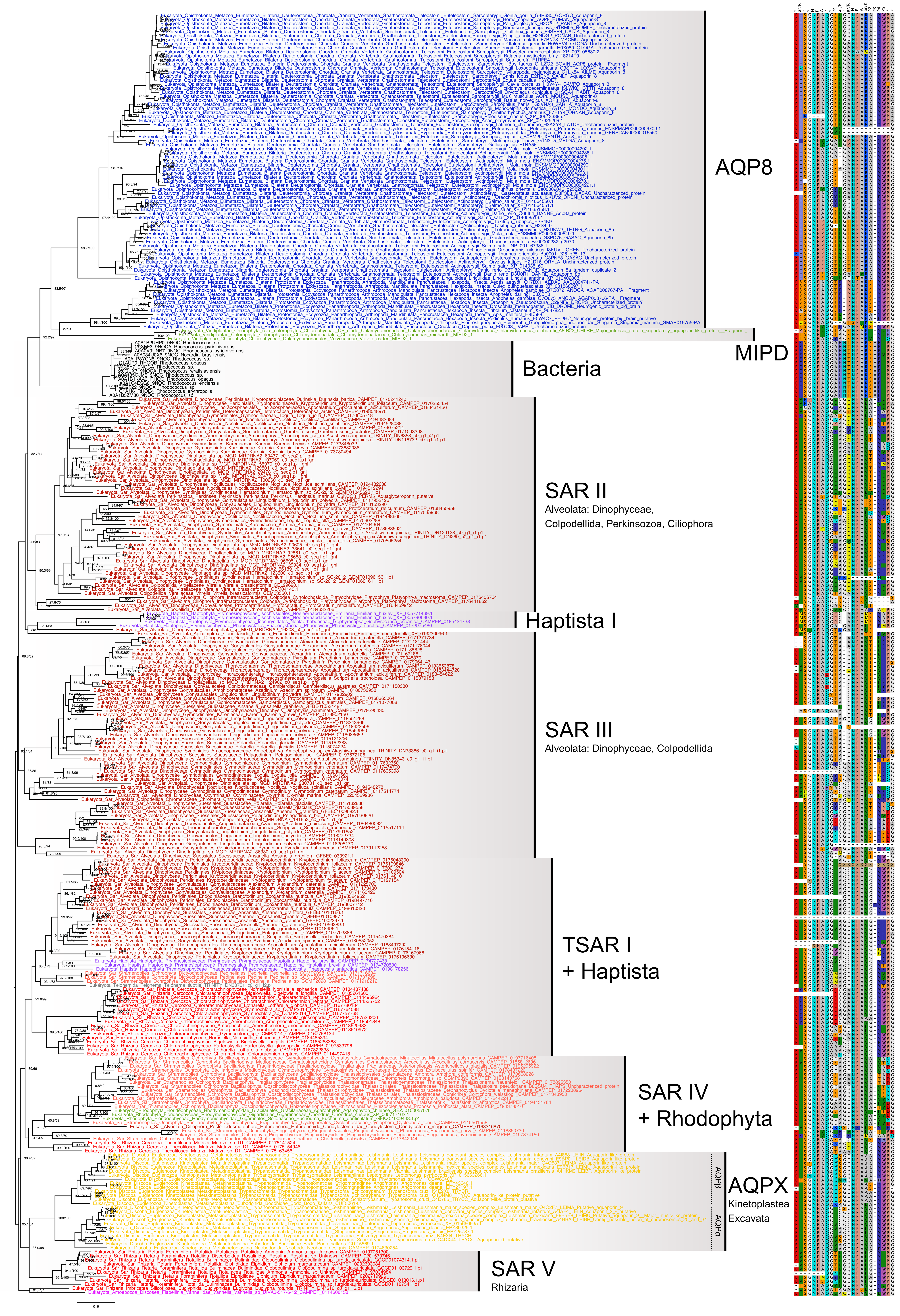
AQP8
MIPD
Bacteria
SAR II
Alveolata: Dinophyceae,
Colpodellida, Perkinsozoa, Ciliophora
Haptista I
SAR III
Alveolata: Dinophyceae, Colpodellida
TSAR I
+ Haptista
SAR IV
+ Rhodophyta
AQPX
Kinetoplastea
Excavata
AQPβ
AQPα
SAR V
Rhizaria
0.6

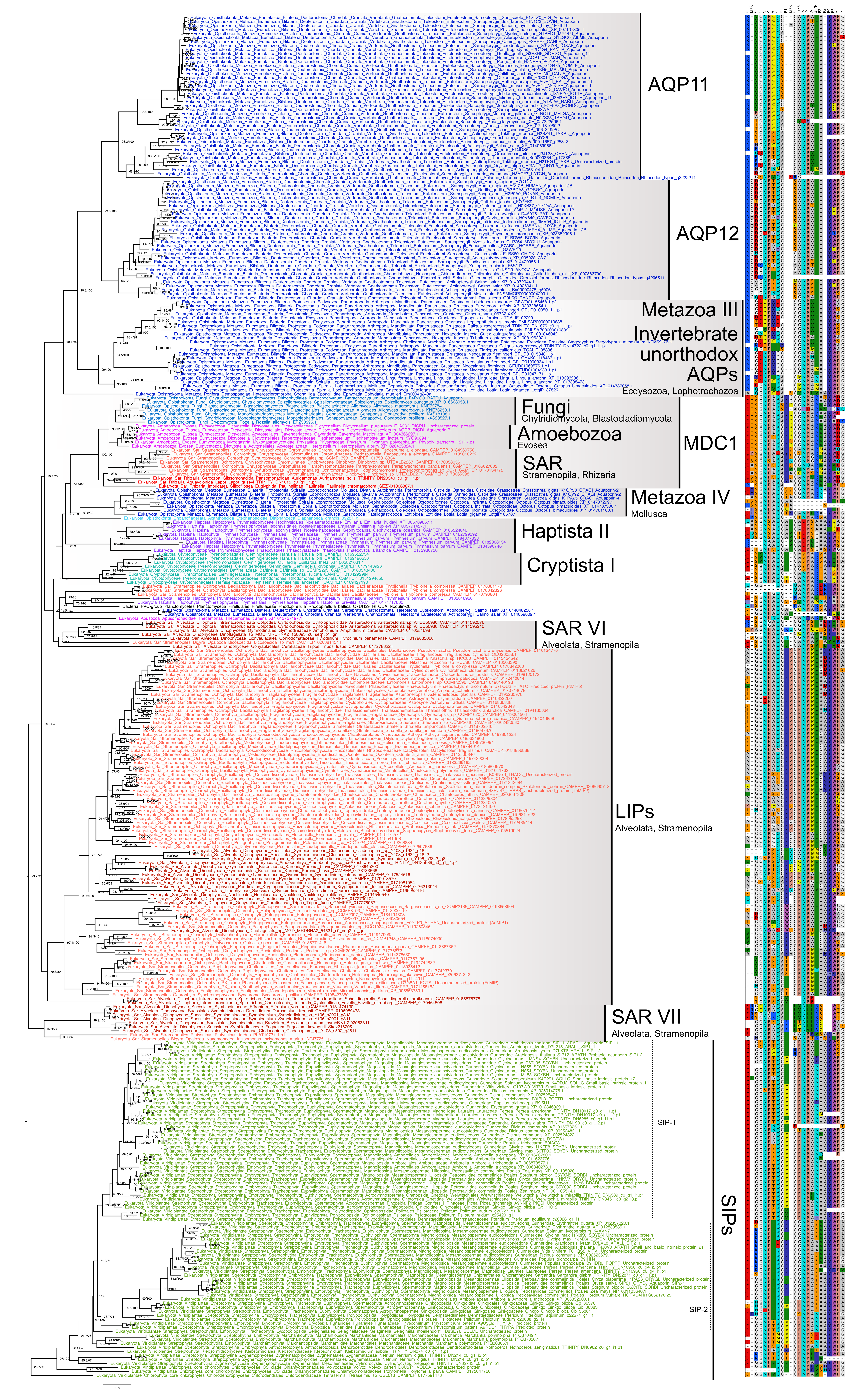
AQP11
AQP12
Metazoa III
Invertebrate
unorthodox
AQPs
Ecdysozoa, Lophotrochozoa
Fungi
Chytridiomycota, Blastocladiomycota
Amoebozoa
Evosea
SAR
Stramenopila, Rhizaria
MDC1
Metazoa IV
Mollusca
Haptista II
Cryptista I
SAR VI
Alveolata, Stramenopila
LIPs
Alveolata, Stramenopila
SAR VII
Alveolata, Stramenopila
SIP-1
SIP-2
SIPs
0.6

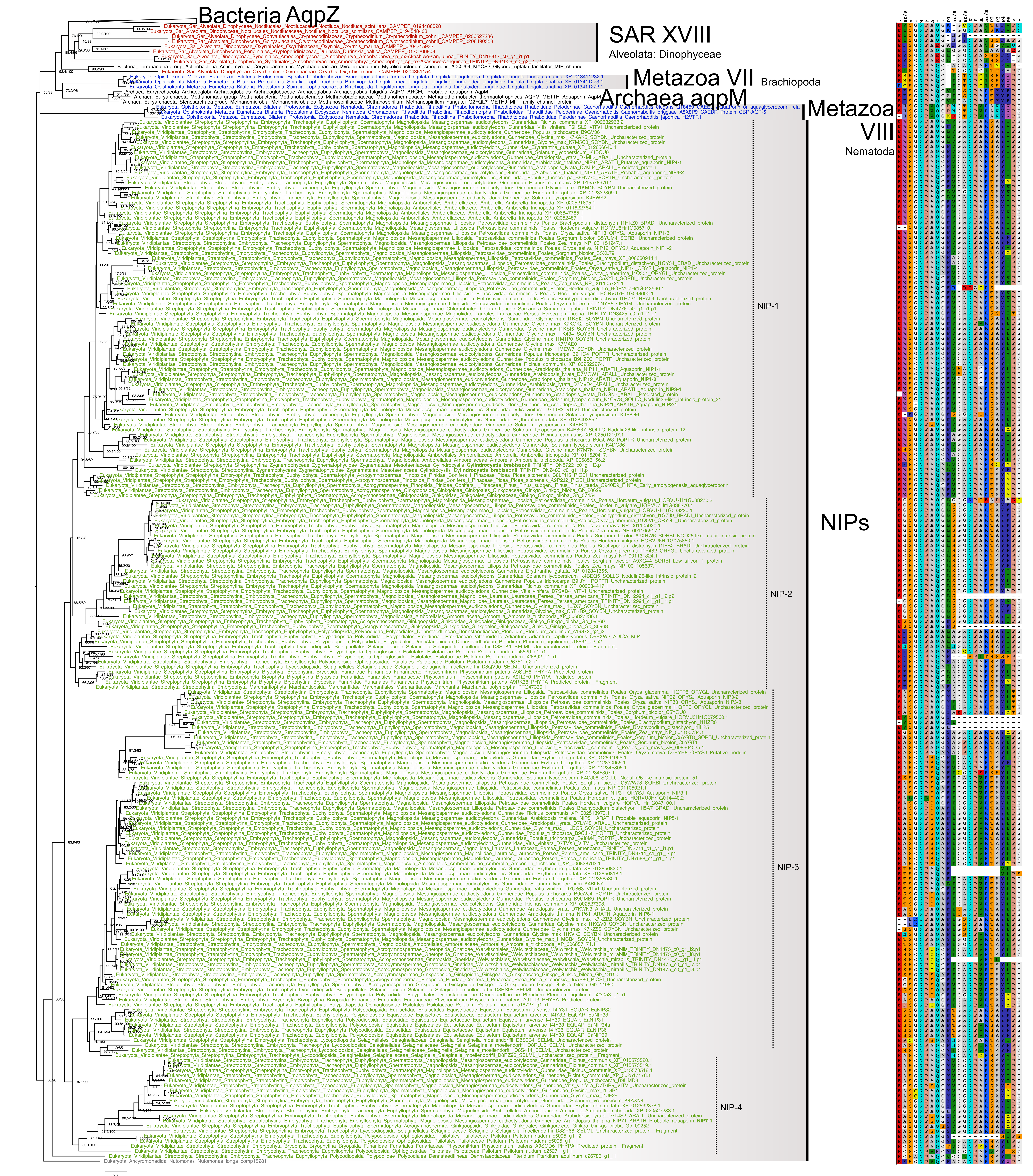

Bacteria AqpZ
SAR XVIII
Alveolata: Dinophyceae
Metazoa VII
Brachiopoda
Archaea aqpM
Metazoa VIII
Nematoda
NIPs
NIP-1
NIP-2
NIP-3
NIP-4

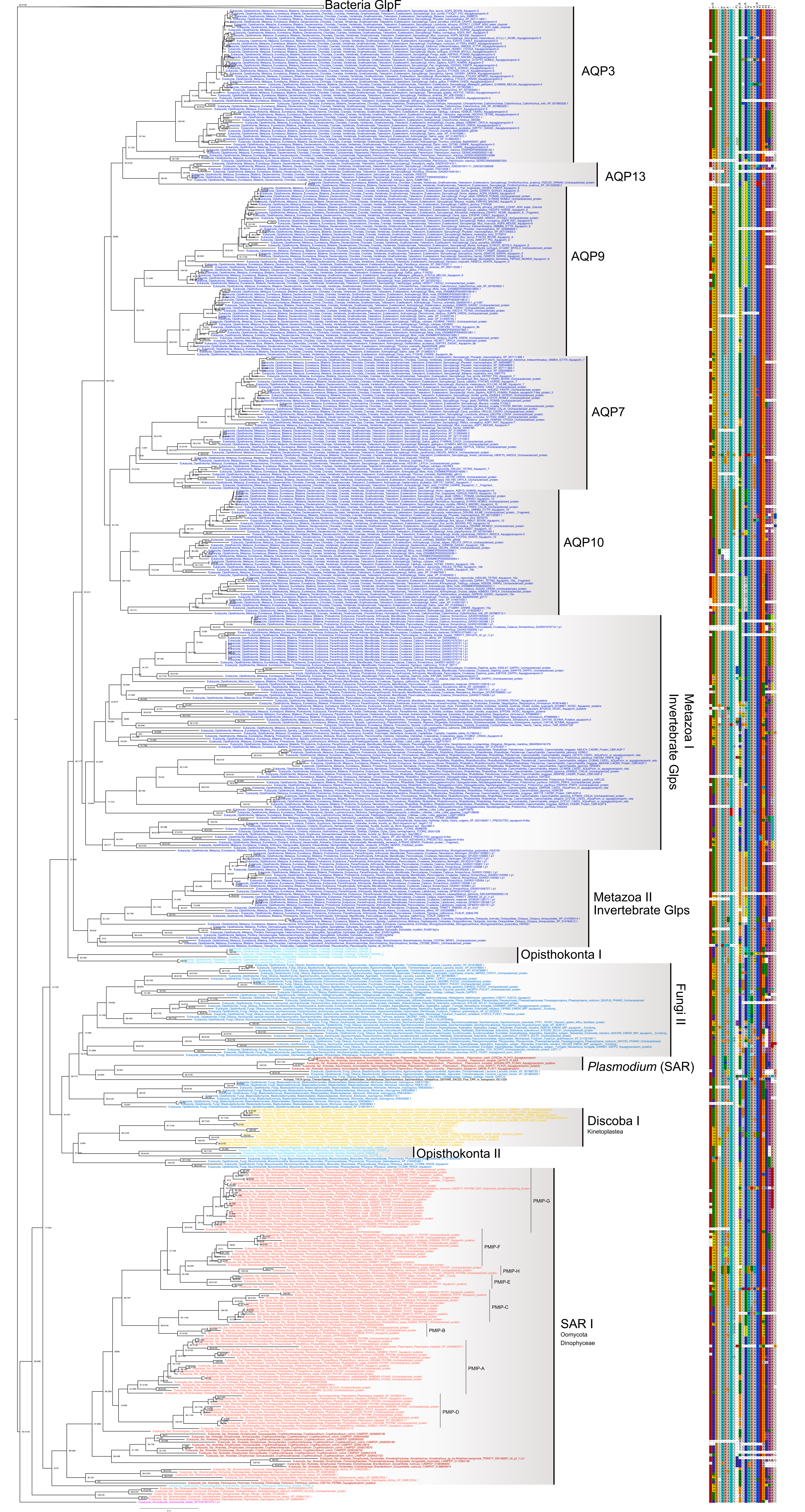
Bacteria GlpF
AQP3
AQP13
AQP9
AQP7
AQP10
Metazoa I
Invertebrate Glps
Metazoa II
Invertebrate Glps
Opisthokonta I
Fungi II
Plasmodium (SAR)
Discoba I
Kinetoplastea
Opisthokonta II
PMIP-G
PMIP-F
PMIP-H
PMIP-E
PMIP-C
PMIP-B
SAR I
Oomycota
Dinophyceae
PMIP-A
PMIP-D

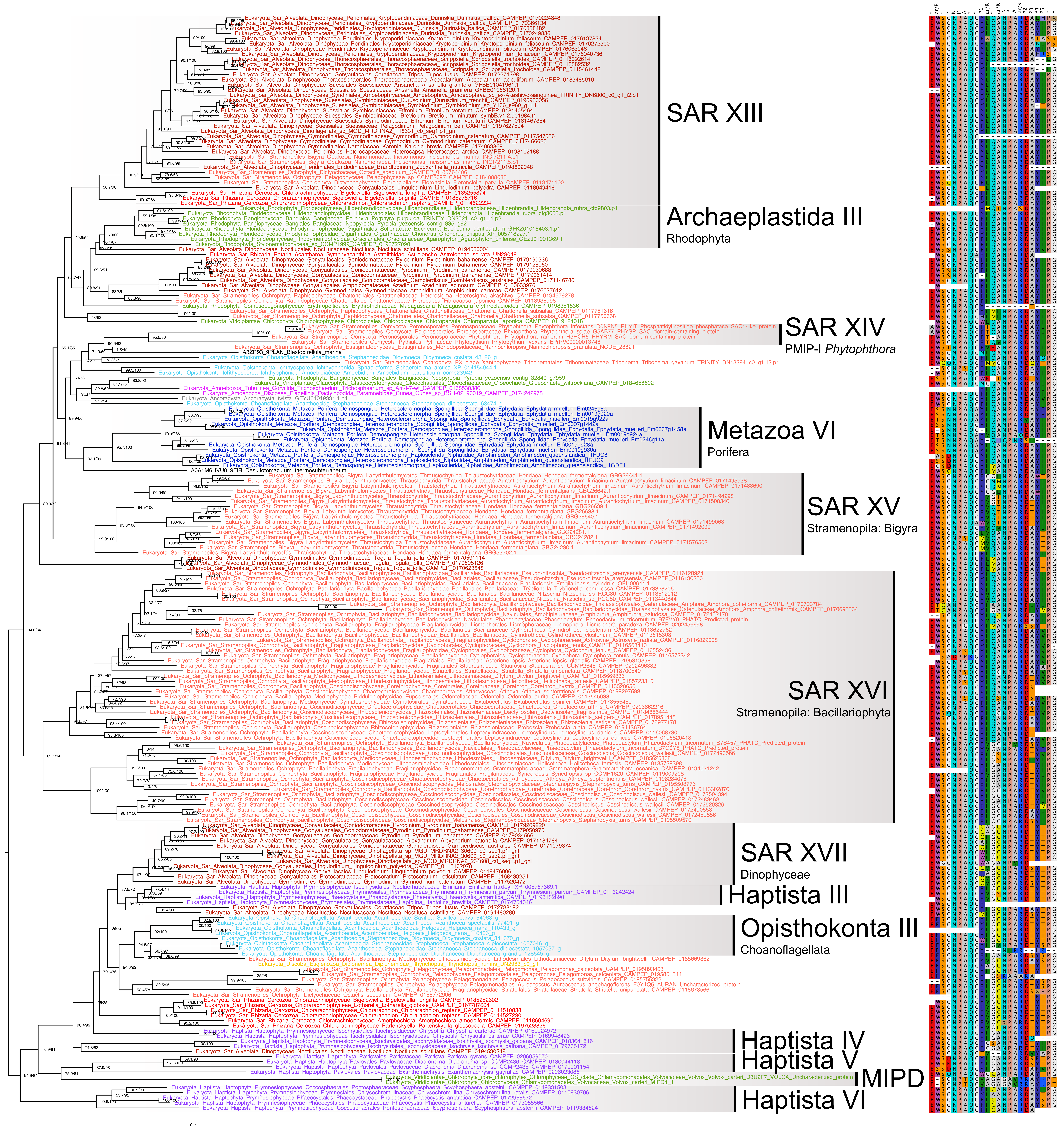
SAR XIII
Archaeplastida III
Rhodophyta
SAR XIV
PMIP-I *Phytophthora*
Metazoa VI
Porifera
SAR XV
Stramenopila: Bigyra
SAR XVI
Stramenopila: Bacillariophyta
SAR XVII
Dinophyceae
Haptista III
Opisthokonta III
Choanoflagellata
Haptista IV
Haptista V
MIPD
Haptista VI
0.4

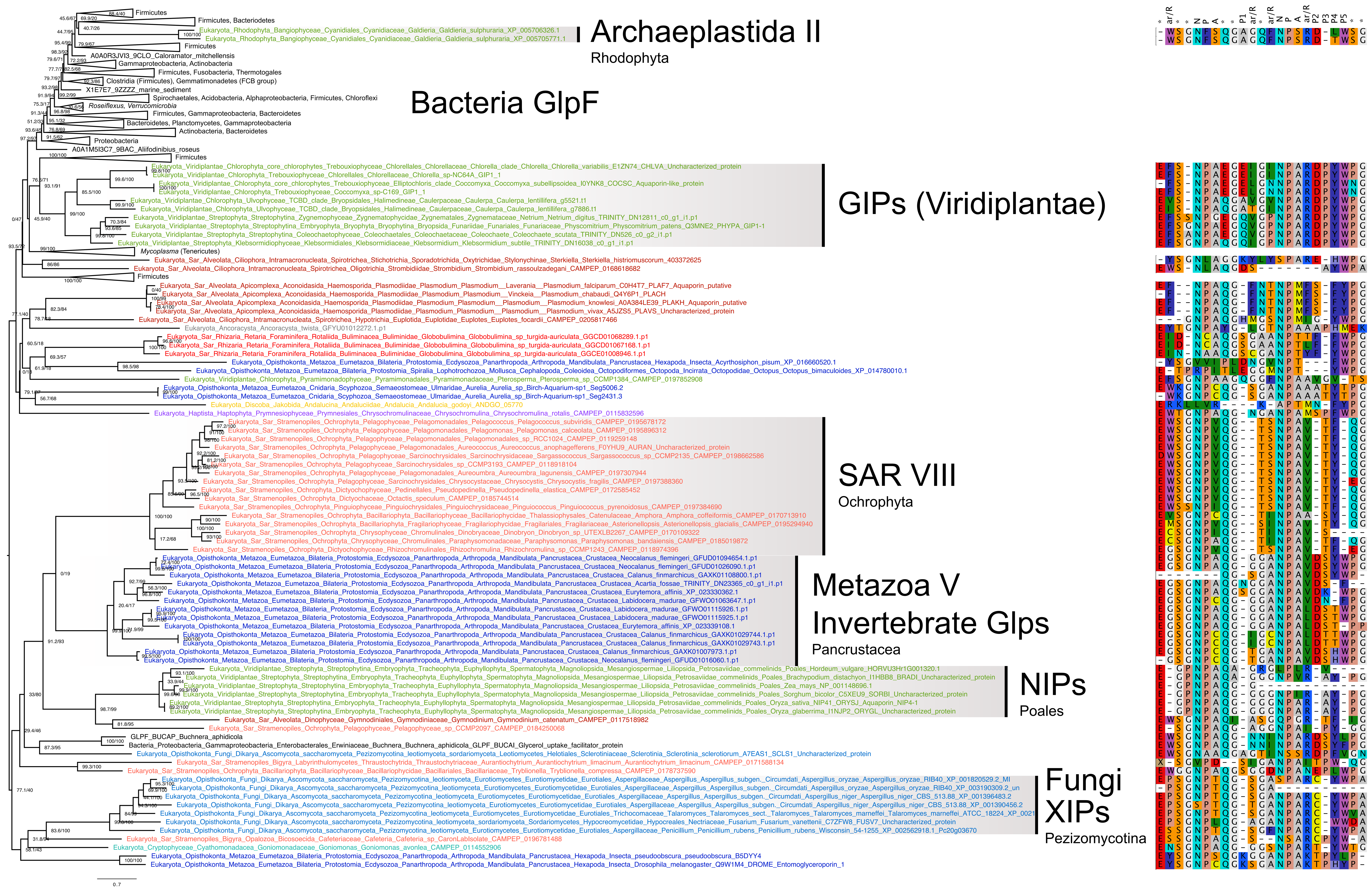

Archaeplastida II
Rhodophyta
Bacteria GlpF
GIPs (Viridiplantae)
SAR VIII
Ochrophyta
Metazoa V
Invertebrate Glps
Pancrustacea
NIPs
Poales
Fungi
XIPs
Pezizomycotina
0.7

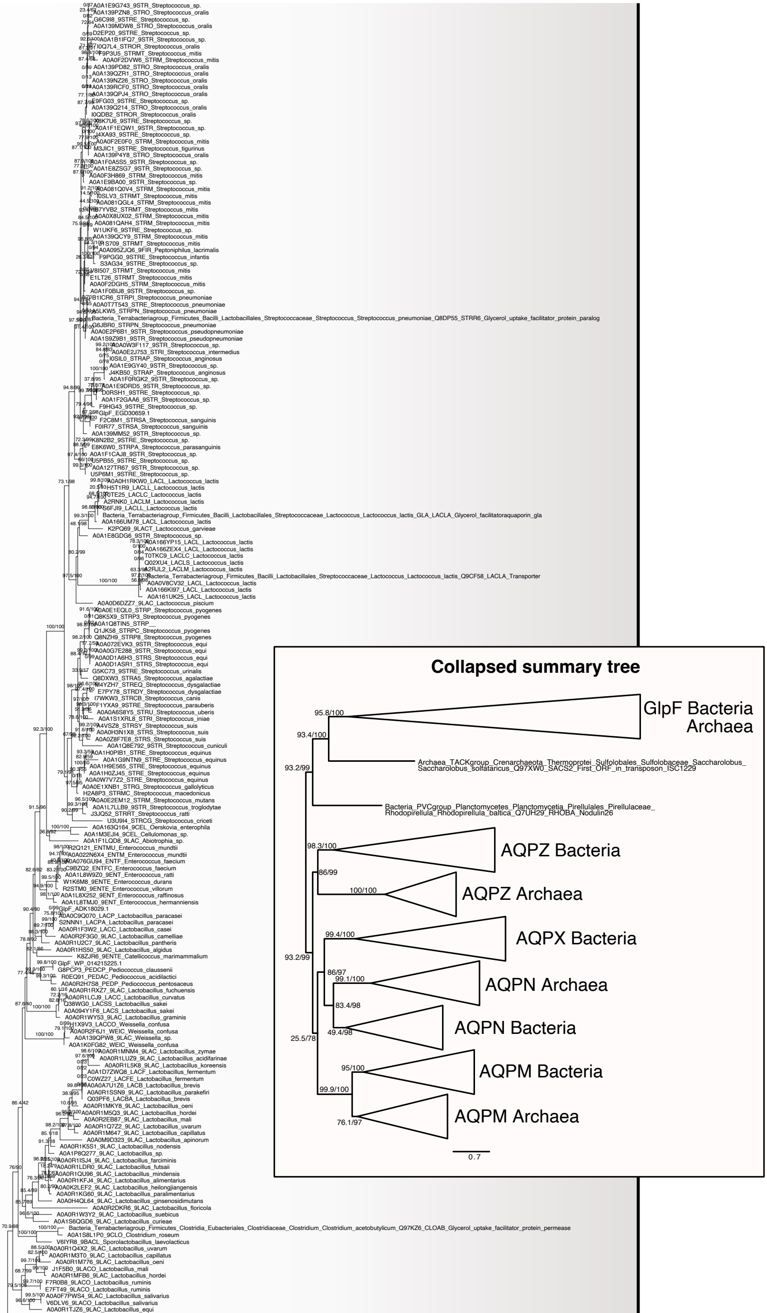
Collapsed summary tree
95.8/100
GlpF Bacteria
Archaea
93.4/100
Archaea_TACKgroup_Crenarchaeota_Thermoprotei_Sulfolobales_Sulfolobaceae_Saccharolobus_
Saccharolobus_solfataricus_Q97XW0_SACS2_First_ORF_in_transposon_ISC1229
93.2/99
Bacteria_PVCgroup_Planctomycetes_Planctomycetia_Pirellulales_Pirellulaceae_
Rhodopirellula_Rhodopirellula_baltica_Q7UH29_RHOBA_Nodulin26
98.3/100
AQPZ Bacteria
86/99
100/100
AQPZ Archaea
99.4/100
AQPX Bacteria
93.2/99
86/97
99.1/100
AQPN Archaea
83.4/98
49.4/98
AQPN Bacteria
25.5/78
95/100
AQPM Bacteria
99.9/100
76.1/97
AQPM Archaea
0.7

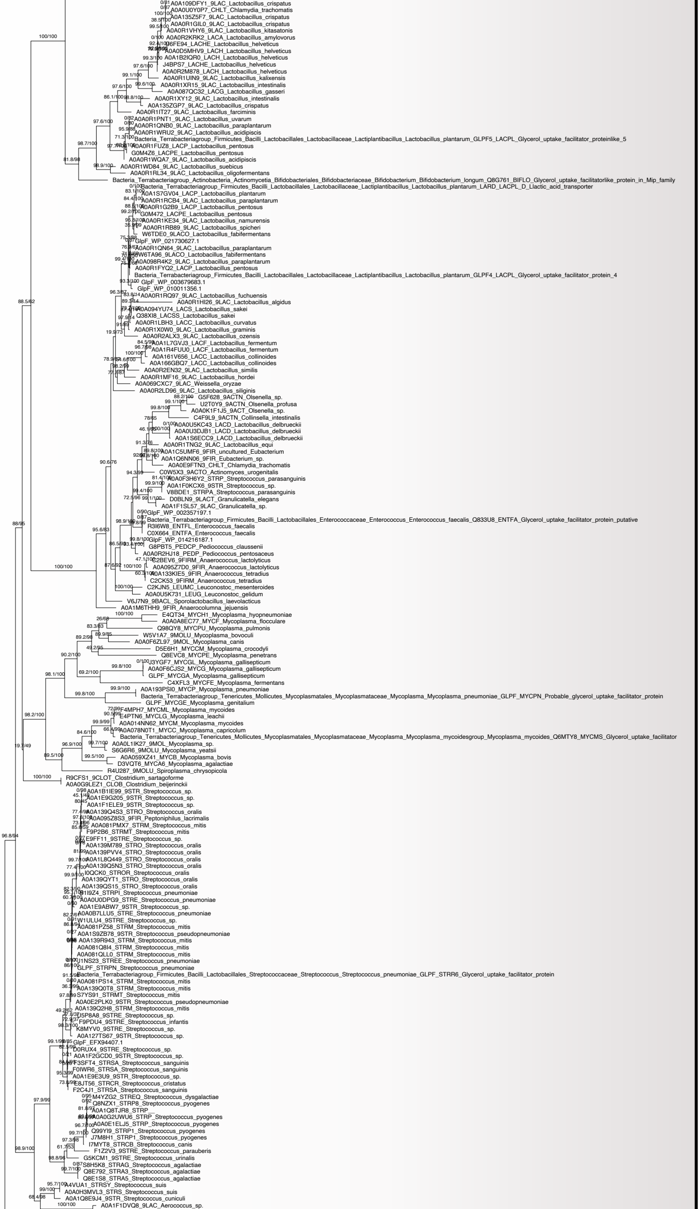
A0A109DFY1_9LAC_Lactobacillus_crispatus
A0A0U0Y0P7_CHLT_Chlamydia_trachomatis
A0A135Z5F7_9LAC_Lactobacillus_crispatus
A0A0R1GIL0_9LAC_Lactobacillus_crispatus
A0A0R1VHY6_9LAC_Lactobacillus_kitasatonis
A0A0R2KRK2_LACA_Lactobacillus_amylovorus
Q6FE94_LACHE_Lactobacillus_helveticus
A0A0D5MHV9_LACH_Lactobacillus_helveticus
A0A1B2IQR0_LACH_Lactobacillus_helveticus
J4BPS7_LACHE_Lactobacillus_helveticus
A0A0R2M878_LACH_Lactobacillus_helveticus
A0A0R1UIN9_9LAC_Lactobacillus_kalixensis
A0A0R1XR15_9LAC_Lactobacillus_intestinalis
A0A087QC32_LACG_Lactobacillus_gasseri
A0A0R1XY12_9LAC_Lactobacillus_intestinalis
A0A135ZGP7_9LAC_Lactobacillus_crispatus
A0A0R1IT27_9LAC_Lactobacillus_farciminis
A0A0R1PNT1_9LAC_Lactobacillus_uvarum
A0A0R1QNB0_9LAC_Lactobacillus_paraplantarum
A0A0R1WRU2_9LAC_Lactobacillus_acidipiscis
Bacteria_Terrabacteriagroup_Firmicutes_Bacilli_Lactobacillales_Lactobacillaceae_Lactiplantibacillus_Lactobacillus_plantarum_GLPF5_LACPL_Glycerol_uptake_facilitator_proteinlike_5
A0A0R1FUZ8_LACP_Lactobacillus_pentosus
G0M4Z6_LACPE_Lactobacillus_pentosus
A0A0R1WQA7_9LAC_Lactobacillus_acidipiscis
A0A0R1WD84_9LAC_Lactobacillus_suebicus
A0A0R1RL34_9LAC_Lactobacillus_oligofermentans
Bacteria_Terrabacteriagroup_Actinobacteria_Actinomycetia_Bifidobacteriales_Bifidobacteriaceae_Bifidobacterium_Bifidobacterium_longum_Q8G761_BIFLO_Glycerol_uptake_facilitatorlike_protein_in_Mip_family
Bacteria_Terrabacteriagroup_Firmicutes_Bacilli_Lactobacillales_Lactobacillaceae_Lactiplantibacillus_Lactobacillus_plantarum_LARD_LACPL_D_Llactic_acid_transporter
A0A1S7GV04_LACP_Lactobacillus_plantarum
A0A0R1RCB4_9LAC_Lactobacillus_paraplantarum
A0A0R1G2B9_LACP_Lactobacillus_pentosus
G0M472_LACPE_Lactobacillus_pentosus
A0A0R1KE34_9LAC_Lactobacillus_namurensis
A0A0R1RB89_9LAC_Lactobacillus_spicheri
W6TDE0_9LACO_Lactobacillus_fabifermentans
GlpF_WP_021730627.1
A0A0R1QN64_9LAC_Lactobacillus_paraplantarum
W6TA96_9LACO_Lactobacillus_fabifermentans
A0A098R4K2_9LAC_Lactobacillus_paraplantarum
A0A0R1FYQ2_LACP_Lactobacillus_pentosus
Bacteria_Terrabacteriagroup_Firmicutes_Bacilli_Lactobacillales_Lactobacillaceae_Lactiplantibacillus_Lactobacillus_plantarum_GLPF4_LACPL_Glycerol_uptake_facilitator_protein_4
GlpF_WP_003679683.1
GlpF_WP_010011356.1
A0A0R1RQ97_9LAC_Lactobacillus_fuchuensis
A0A0R1HI26_9LAC_Lactobacillus_algidus
A0A094YU74_LACS_Lactobacillus_sakei
Q38XI8_LACSS_Lactobacillus_sakei
A0A0R1LBH3_LACC_Lactobacillus_curvatus
A0A0R1X0W0_9LAC_Lactobacillus_graminis
A0A0R2ALX3_9LAC_Lactobacillus_ozensis
A0A1L7GVJ3_LACF_Lactobacillus_fermentum
A0A1R4FUU0_LACF_Lactobacillus_fermentum
A0A161V656_LACC_Lactobacillus_collinoides
A0A166GBQ7_LACC_Lactobacillus_collinoides
A0A0R2EN32_9LAC_Lactobacillus_similis
A0A0R1MF16_9LAC_Lactobacillus_hordei
A0A069CXC7_9LAC_Weissella_oryzae
A0A0R2LD96_9LAC_Lactobacillus_siliginis
G5F628_9ACTN_Olsenella_sp.
U2T0Y9_9ACTN_Olsenella_profusa
A0A0K1F1J5_9ACT_Olsenella_sp.
C4F9L9_9ACTN_Collinsella_intestinalis
A0A0U5KC43_LACD_Lactobacillus_delbrueckii
A0A0U3DJB1_LACD_Lactobacillus_delbrueckii
A0A1S6ECC9_LACD_Lactobacillus_delbrueckii
A0A0R1TNG2_9LAC_Lactobacillus_equi
A0A1C5UMF6_9FIR_uncultured_Eubacterium
A0A1Q6NN06_9FIR_Eubacterium_sp.
A0A0E9FTN3_CHLT_Chlamydia_trachomatis
C0W5X3_9ACTO_Actinomyces_urogenitalis
A0A0F3H6Y2_STRP_Streptococcus_parasanguinis
A0A1F0KCX6_9STR_Streptococcus_sp.
V8BDE1_STRPA_Streptococcus_parasanguinis
D0BLN9_9LACT_Granulicatella_elegans
A0A1F1SL57_9LAC_Granulicatella_sp.
GlpF_WP_002357197.1
Bacteria_Terrabacteriagroup_Firmicutes_Bacilli_Lactobacillales_Enterococcaceae_Enterococcus_Enterococcus_faecalis_Q833U8_ENTFA_Glycerol_uptake_facilitator_protein_putative
R3I6W8_ENTFL_Enterococcus_faecalis
C0X664_ENTFA_Enterococcus_faecalis
GlpF_WP_014216187.1
G8PBT5_PEDCP_Pediococcus_claussenii
A0A0R2HJ18_PEDP_Pediococcus_pentosaceus
C2BEV6_9FIRM_Anaerococcus_lactolyticus
A0A095Z7D0_9FIR_Anaerococcus_lactolyticus
A0A133KIE5_9FIR_Anaerococcus_tetradius
C2CK53_9FIRM_Anaerococcus_tetradius
C2KJN5_LEUMC_Leuconostoc_mesenteroides
A0A0U5K731_LEUG_Leuconostoc_gelidum
V6J7N9_9BACL_Sporolactobacillus_laevolacticus
A0A1M6THH9_9FIR_Anaerocolumna_jejuensis
E4QT34_MYCH1_Mycoplasma_hyopneumoniae
A0A0A8EC77_MYCF_Mycoplasma_flocculare
Q98QY8_MYCPU_Mycoplasma_pulmonis
W5V1A7_9MOLU_Mycoplasma_bovoculi
A0A0F6ZL97_9MOL_Mycoplasma_canis
D5E6H1_MYCCM_Mycoplasma_crocodyli
Q8EVC8_MYCPE_Mycoplasma_penetrans
J3YGF7_MYCGL_Mycoplasma_gallisepticum
A0A0F6CJS2_MYCG_Mycoplasma_gallisepticum
GLPF_MYCGA_Mycoplasma_gallisepticum
C4XFL3_MYCFE_Mycoplasma_fermentans
A0A193PSI0_MYCP_Mycoplasma_pneumoniae
Bacteria_Terrabacteriagroup_Tenericutes_Mollicutes_Mycoplasmatales_Mycoplasmataceae_Mycoplasma_Mycoplasma_pneumoniae_GLPF_MYCPN_Probable_glycerol_uptake_facilitator_protein
GLPF_MYCGE_Mycoplasma_genitalium
F4MPH7_MYCML_Mycoplasma_mycoides
E4PTN6_MYCLG_Mycoplasma_leachii
A0A014NN62_MYCM_Mycoplasma_mycoides
A0A078N0T1_MYCC_Mycoplasma_capricolum
Bacteria_Terrabacteriagroup_Tenericutes_Mollicutes_Mycoplasmatales_Mycoplasmataceae_Mycoplasma_Mycoplasma_mycoidesgroup_Mycoplasma_mycoides_Q6MTY8_MYCMS_Glycerol_uptake_facilitator
A0A0L1IK27_9MOL_Mycoplasma_sp.
S6G6R6_9MOLU_Mycoplasma_yeatsii
A0A059XZ41_MYCB_Mycoplasma_bovis
D3VQT6_MYCA6_Mycoplasma_agalactiae
R4U287_9MOLU_Spiroplasma_chrysopicola
R9CFS1_9CLOT_Clostridium_sartagoforme
A0A0G9LEZ1_CLOB_Clostridium_beijerinckii
A0A1B1IE99_9STR_Streptococcus_sp.
A0A1E9G205_9STR_Streptococcus_sp.
A0A1F1ELE9_9STR_Streptococcus_sp.
A0A139Q4S3_STRO_Streptococcus_oralis
A0A095Z8S3_9FIR_Peptoniphilus_lacrimalis
A0A081PMX7_STRM_Streptococcus_mitis
F9P2B6_STRMT_Streptococcus_mitis
E9FF11_9STRE_Streptococcus_sp.
A0A139M789_STRO_Streptococcus_oralis
A0A139PVV4_STRO_Streptococcus_oralis
A0A1L8Q449_STRO_Streptococcus_oralis
A0A139Q5N3_STRO_Streptococcus_oralis
I0QCK0_STROR_Streptococcus_oralis
A0A139QYT1_STRO_Streptococcus_oralis
A0A139QS15_STRO_Streptococcus_oralis
B1I9Z4_STRPI_Streptococcus_pneumoniae
A0A0U0DPG9_STRE_Streptococcus_pneumoniae
A0A1E9ABW7_9STR_Streptococcus_sp.
A0A0B7LLU5_STRE_Streptococcus_pneumoniae
W1ULU4_9STRE_Streptococcus_sp.
A0A081PZ58_STRM_Streptococcus_mitis
A0A1S9ZB78_9STR_Streptococcus_pseudopneumoniae
A0A139R943_STRM_Streptococcus_mitis
A0A081Q8I4_STRM_Streptococcus_mitis
A0A081QLL0_STRM_Streptococcus_mitis
Q1NS23_STREE_Streptococcus_pneumoniae
GLPF_STRPN_Streptococcus_pneumoniae
Bacteria_Terrabacteriagroup_Firmicutes_Bacilli_Lactobacillales_Streptococcaceae_Streptococcus_Streptococcus_pneumoniae_GLPF_STRR6_Glycerol_uptake_facilitator_protein
A0A081PS14_STRM_Streptococcus_mitis
A0A139Q0T8_STRM_Streptococcus_mitis
S7YS91_STRMT_Streptococcus_mitis
A0A0E2PLK0_9STR_Streptococcus_pseudopneumoniae
A0A139Q2H8_STRM_Streptococcus_mitis
U5P8A8_9STRE_Streptococcus_sp.
F9PDU4_9STRE_Streptococcus_infantis
K8MYV0_9STRE_Streptococcus_sp.
A0A127TS67_9STR_Streptococcus_sp.
GlpF_EFX94407.1
D0RUX4_9STRE_Streptococcus_sp.
A0A1F2GCD0_9STR_Streptococcus_sp.
F3SFT4_STRSA_Streptococcus_sanguinis
F0IWR6_STRSA_Streptococcus_sanguinis
A0A1E9E3U9_9STR_Streptococcus_sp.
E8JT56_STRCR_Streptococcus_cristatus
F2C4J1_STRSA_Streptococcus_sanguinis
M4YZG2_STREQ_Streptococcus_dysgalactiae
Q8NZX1_STRP8_Streptococcus_pyogenes
A0A1Q8TJR8_STRP_
A0A0G2UWU6_STRP_Streptococcus_pyogenes
A0A0E1ELJ5_STRP_Streptococcus_pyogenes
Q99YI9_STRP1_Streptococcus_pyogenes
J7M8H1_STRP1_Streptococcus_pyogenes
I7MYT8_STRCB_Streptococcus_canis
F1Z2V3_9STRE_Streptococcus_parauberis
G5KCM1_9STRE_Streptococcus_urinalis
S8H5K8_STRAG_Streptococcus_agalactiae
Q8E792_STRA3_Streptococcus_agalactiae
Q8E1S8_STRA5_Streptococcus_agalactiae
A4VUA1_STRSY_Streptococcus_suis
A0A0H3MVL3_STRS_Streptococcus_suis
A0A1Q8E9J4_9STR_Streptococcus_cuniculi
A0A1F1DVQ8_9LAC_Aerococcus_sp.

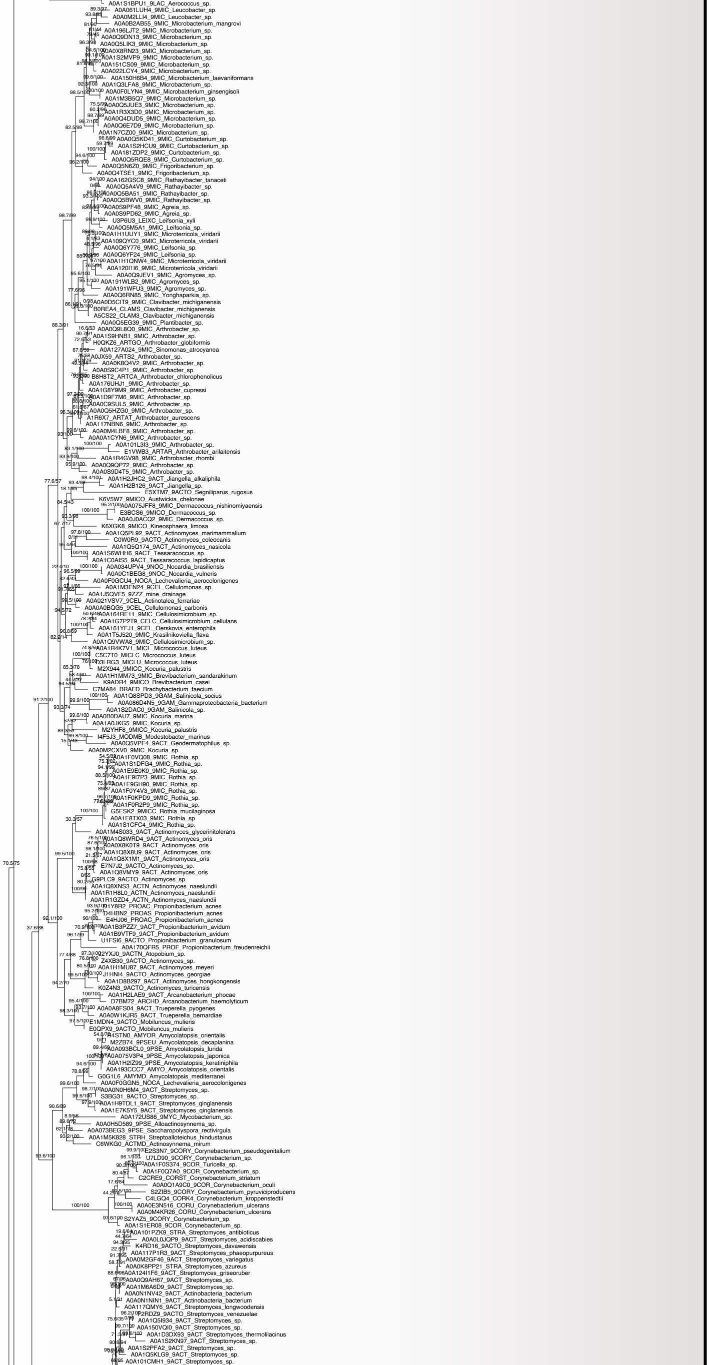
A0A1S1BPU1_9LAC_Aerococcus_sp.
A0A061LUH4_9MIC_Leucobacter_sp.
A0A0M2LLI4_9MIC_Leucobacter_sp.
A0A0B2AB55_9MIC_Microbacterium_mangrovi
A0A196LJT2_9MIC_Microbacterium_sp.
A0A0Q9DN13_9MIC_Microbacterium_sp.
A0A0Q5LIK3_9MIC_Microbacterium_sp.
A0A0X8RN23_9MIC_Microbacterium_sp.
A0A1S2MVP9_9MIC_Microbacterium_sp.
A0A151CS09_9MIC_Microbacterium_sp.
A0A022LCY4_9MIC_Microbacterium_sp.
A0A150H6B4_9MIC_Microbacterium_laevaniformans
A0A1Q3LFA8_9MIC_Microbacterium_sp.
A0A0F0LYN4_9MIC_Microbacterium_ginsengisoli
A0A1M3B5Q7_9MIC_Microbacterium_sp.
A0A0Q5JUE3_9MIC_Microbacterium_sp.
A0A1R3X3D0_9MIC_Microbacterium_sp.
A0A0Q4DUD5_9MIC_Microbacterium_sp.
A0A0Q6E7D9_9MIC_Microbacterium_sp.
A0A1N7CZ00_9MIC_Microbacterium_sp.
A0A0Q5KD41_9MIC_Curtobacterium_sp.
A0A1S2HCU9_9MIC_Curtobacterium_sp.
A0A181ZDP2_9MIC_Curtobacterium_sp.
A0A0Q5RQE8_9MIC_Curtobacterium_sp.
A0A0Q5N6Z0_9MIC_Frigoribacterium_sp.
A0A0Q4TSE1_9MIC_Frigoribacterium_sp.
A0A162GSC8_9MIC_Rathayibacter_tanaceti
A0A0Q5A4V9_9MIC_Rathayibacter_sp.
A0A0Q5BA51_9MIC_Rathayibacter_sp.
A0A0Q5BWV0_9MIC_Rathayibacter_sp.
A0A0S9PF48_9MIC_Agreia_sp.
A0A0S9PD62_9MIC_Agreia_sp.
U3P6U3_LEIXC_Leifsonia_xyli
A0A0Q5M5A1_9MIC_Leifsonia_sp.
A0A1H1UUY1_9MIC_Microterricola_viridarii
A0A109QYC0_9MIC_Microterricola_viridarii
A0A0Q6Y776_9MIC_Leifsonia_sp.
A0A0Q6YF24_9MIC_Leifsonia_sp.
A0A1H1QNW4_9MIC_Microterricola_viridarii
A0A120I1I6_9MIC_Microterricola_viridarii
A0A0Q9JEV1_9MIC_Agromyces_sp.
A0A191WLB2_9MIC_Agromyces_sp.
A0A191WFU3_9MIC_Agromyces_sp.
A0A0Q6RN85_9MIC_Yonghaparkia_sp.
A0A0D5CIT9_9MIC_Clavibacter_michiganensis
B0REA4_CLAMS_Clavibacter_michiganensis
A5CS22_CLAM3_Clavibacter_michiganensis
A0A0Q5EG39_9MIC_Plantibacter_sp.
A0A0Q9L8Q0_9MIC_Arthrobacter_sp.
A0A1S9HNB1_9MIC_Arthrobacter_sp.
H0QKZ6_ARTGO_Arthrobacter_globiformis
A0A127A024_9MIC_Sinomonas_atrocyanea
A0JX59_ARTS2_Arthrobacter_sp.
A0A0K8Q4V2_9MIC_Arthrobacter_sp.
A0A0S9C4P1_9MIC_Arthrobacter_sp.
B8H8T2_ARTCA_Arthrobacter_chlorophenolicus
A0A176UHJ1_9MIC_Arthrobacter_sp.
A0A1G8Y9M9_9MIC_Arthrobacter_cupressi
A0A1D9F7M6_9MIC_Arthrobacter_sp.
A0A0C9SUL5_9MIC_Arthrobacter_sp.
A0A0Q5HZG0_9MIC_Arthrobacter_sp.
A1R6X7_ARTAT_Arthrobacter_aurescens
A0A117NBN6_9MIC_Arthrobacter_sp.
A0A0M4LBF8_9MIC_Arthrobacter_sp.
A0A0A1CYN6_9MIC_Arthrobacter_sp.
A0A101L3I3_9MIC_Arthrobacter_sp.
E1VWB3_ARTAR_Arthrobacter_arilaitensis
A0A1R4GV98_9MIC_Arthrobacter_rhombi
A0A0Q9QP72_9MIC_Arthrobacter_sp.
A0A0S9D4T5_9MIC_Arthrobacter_sp.
A0A1H2JHC2_9ACT_Jiangella_alkaliphila
A0A1H2B126_9ACT_Jiangella_sp.
E5XTM7_9ACTO_Segniliparus_rugosus
K6V5W7_9MICO_Austwickia_chelonae
A0A075JFF8_9MIC_Dermacoccus_nishinomiyaensis
E3BCS6_9MICO_Dermacoccus_sp.
A0A0J0ACQ2_9MIC_Dermacoccus_sp.
K6XGK8_9MICO_Kineosphaera_limosa
A0A1Q5PL92_9ACT_Actinomyces_marimammalium
C0W0R9_9ACTO_Actinomyces_coleocanis
A0A1Q5Q174_9ACT_Actinomyces_nasicola
A0A1S6WHH6_9ACT_Tessaracoccus_sp.
A0A1C0AIS5_9ACT_Tessaracoccus_lapidicaptus
A0A034UPV4_9NOC_Nocardia_brasiliensis
A0A0C1BEG8_9NOC_Nocardia_vulneris
A0A0F0GCU4_NOCA_Lechevalieria_aerocolonigenes
A0A1M3EN24_9CEL_Cellulomonas_sp.
A0A1J5QVF5_9ZZZ_mine_drainage
A0A021VSV7_9CEL_Actinotalea_ferrariae
A0A0A0BQG5_9CEL_Cellulomonas_carbonis
A0A164RE11_9MIC_Cellulosimicrobium_sp.
A0A1G7P2T9_CELC_Cellulosimicrobium_cellulans
A0A161YFJ1_9CEL_Oerskovia_enterophila
A0A1T5J520_9MIC_Krasilnikoviella_flava
A0A1Q9VWA8_9MIC_Cellulosimicrobium_sp.
A0A1R4K7V1_MICL_Micrococcus_luteus
C5C7T0_MICLC_Micrococcus_luteus
D3LRG3_MICLU_Micrococcus_luteus
M2X944_9MICC_Kocuria_palustris
A0A1H1MM73_9MIC_Brevibacterium_sandarakinum
K9ADR4_9MICO_Brevibacterium_casei
C7MA84_BRAFD_Brachybacterium_faecium
A0A1Q8SPD3_9GAM_Salinicola_socius
A0A086D4N5_9GAM_Gammaproteobacteria_bacterium
A0A1S2DAC0_9GAM_Salinicola_sp.
A0A0B0DAU7_9MIC_Kocuria_marina
A0A1A0JKG5_9MIC_Kocuria_sp.
M2YHF8_9MICC_Kocuria_palustris
I4F5J3_MODMB_Modestobacter_marinus
A0A0Q5VPE4_9ACT_Geodermatophilus_sp.
A0A0M2CXV0_9MIC_Kocuria_sp.
A0A1F0VQ08_9MIC_Rothia_sp.
A0A1S1DFG4_9MIC_Rothia_sp.
A0A1E9E0K0_9MIC_Rothia_sp.
A0A1E9I7P3_9MIC_Rothia_sp.
A0A1E9GH90_9MIC_Rothia_sp.
A0A1F0Y4V3_9MIC_Rothia_sp.
A0A1F0KPD9_9MIC_Rothia_sp.
A0A1F0R2P9_9MIC_Rothia_sp.
G5ESK2_9MICC_Rothia_mucilaginosa
A0A1E8TX03_9MIC_Rothia_sp.
A0A1S1CFC4_9MIC_Rothia_sp.
A0A1M4S033_9ACT_Actinomyces_glycerinitolerans
A0A1Q8WRD4_9ACT_Actinomyces_oris
A0A0X8K0T9_9ACT_Actinomyces_oris
A0A1Q8X8U9_9ACT_Actinomyces_oris
A0A1Q8X1M1_9ACT_Actinomyces_oris
E7N7J2_9ACTO_Actinomyces_sp.
A0A1Q8VMY9_9ACT_Actinomyces_oris
G9PLC9_9ACTO_Actinomyces_sp.
A0A1Q8XNS3_ACTN_Actinomyces_naeslundii
A0A1R1H8L0_ACTN_Actinomyces_naeslundii
A0A1R1GZD4_ACTN_Actinomyces_naeslundii
D1Y8R2_PROAC_Propionibacterium_acnes
D4HBN2_PROAS_Propionibacterium_acnes
E4HJ06_PROAC_Propionibacterium_acnes
A0A1B3PZZ7_9ACT_Propionibacterium_avidum
A0A1B9VTF9_9ACT_Propionibacterium_avidum
U1FSI6_9ACTO_Propionibacterium_granulosum
A0A170QFR5_PROF_Propionibacterium_freudenreichii
J2YXJ0_9ACTN_Atopobium_sp.
Z4XB30_9ACTO_Actinomyces_sp.
A0A1H1MU87_9ACT_Actinomyces_meyeri
J1HNI4_9ACTO_Actinomyces_georgiae
A0A1D8B297_9ACT_Actinomyces_hongkongensis
K0Z4N3_9ACTO_Actinomyces_turicensis
A0A1H2LAE9_9ACT_Arcanobacterium_phocae
D7BM72_ARCHD_Arcanobacterium_haemolyticum
A0A0A8FS04_9ACT_Trueperella_pyogenes
A0A0W1KJR5_9ACT_Trueperella_bernardiae
E1MDN4_9ACTO_Mobiluncus_mulieris
E0QPX9_9ACTO_Mobiluncus_mulieris
R4STN0_AMYOR_Amycolatopsis_orientalis
M2ZB74_9PSEU_Amycolatopsis_decaplanina
A0A093BCL0_9PSE_Amycolatopsis_lurida
A0A075V3P4_9PSE_Amycolatopsis_japonica
A0A1H2IZ99_9PSE_Amycolatopsis_keratiniphila
A0A193CCC7_AMYO_Amycolatopsis_orientalis
G0G1L6_AMYMD_Amycolatopsis_mediterranei
A0A0F0GGN5_NOCA_Lechevalieria_aerocolonigenes
A0A0N0H6M4_9ACT_Streptomyces_sp.
S3BG31_9ACTO_Streptomyces_sp.
A0A1H9TDL1_9ACT_Streptomyces_qinglanensis
A0A1E7K5Y5_9ACT_Streptomyces_qinglanensis
A0A172US86_9MYC_Mycobacterium_sp.
A0A0H5D589_9PSE_Alloactinosynnema_sp.
A0A073BEG3_9PSE_Saccharopolyspora_rectivirgula
A0A1M5K828_STRH_Streptoalloteichus_hindustanus
C6WKG0_ACTMD_Actinosynnema_mirum
E2S3N7_9CORY_Corynebacterium_pseudogenitalium
U7LD90_9CORY_Corynebacterium_sp.
A0A1F0S374_9COR_Turicella_sp.
A0A1F0Q7A0_9COR_Corynebacterium_sp.
C2CRE9_CORST_Corynebacterium_striatum
A0A0Q1A9C0_9COR_Corynebacterium_oculi
S2ZIB5_9CORY_Corynebacterium_pyruviciproducens
C4LGQ4_CORK4_Corynebacterium_kroppenstedtii
A0A0E3N516_CORU_Corynebacterium_ulcerans
A0A0M4KR26_CORU_Corynebacterium_ulcerans
S2YAZ5_9CORY_Corynebacterium_sp.
A0A1S1ER08_9COR_Corynebacterium_sp.
A0A101PZK9_STRA_Streptomyces_antibioticus
A0A0L0JQP9_9ACT_Streptomyces_acidiscabies
K4RD16_9ACTO_Streptomyces_davawensis
A0A117P1R3_9ACT_Streptomyces_phaeopurpureus
A0A0M2GF46_9ACT_Streptomyces_variegatus
A0A0K8PP21_STRA_Streptomyces_azureus
A0A124I1F6_9ACT_Streptomyces_griseoruber
A0A0Q9AH67_9ACT_Streptomyces_sp.
A0A1M6A6D9_9ACT_Streptomyces_sp.
A0A0N1NV42_9ACT_Actinobacteria_bacterium
A0A0N1NIN1_9ACT_Actinobacteria_bacterium
A0A117QMY6_9ACT_Streptomyces_longwoodensis
F2RDZ9_9ACTO_Streptomyces_venezuelae
A0A1Q5I934_9ACT_Streptomyces_sp.
A0A150VQI0_9ACT_Streptomyces_sp.
A0A1D3DX93_9ACT_Streptomyces_thermolilacinus
A0A1S2KN97_9ACT_Streptomyces_sp.
A0A1S2PFA2_9ACT_Streptomyces_sp.
A0A1Q5KLG9_9ACT_Streptomyces_sp.
A0A101CMH1_9ACT_Streptomyces_sp.

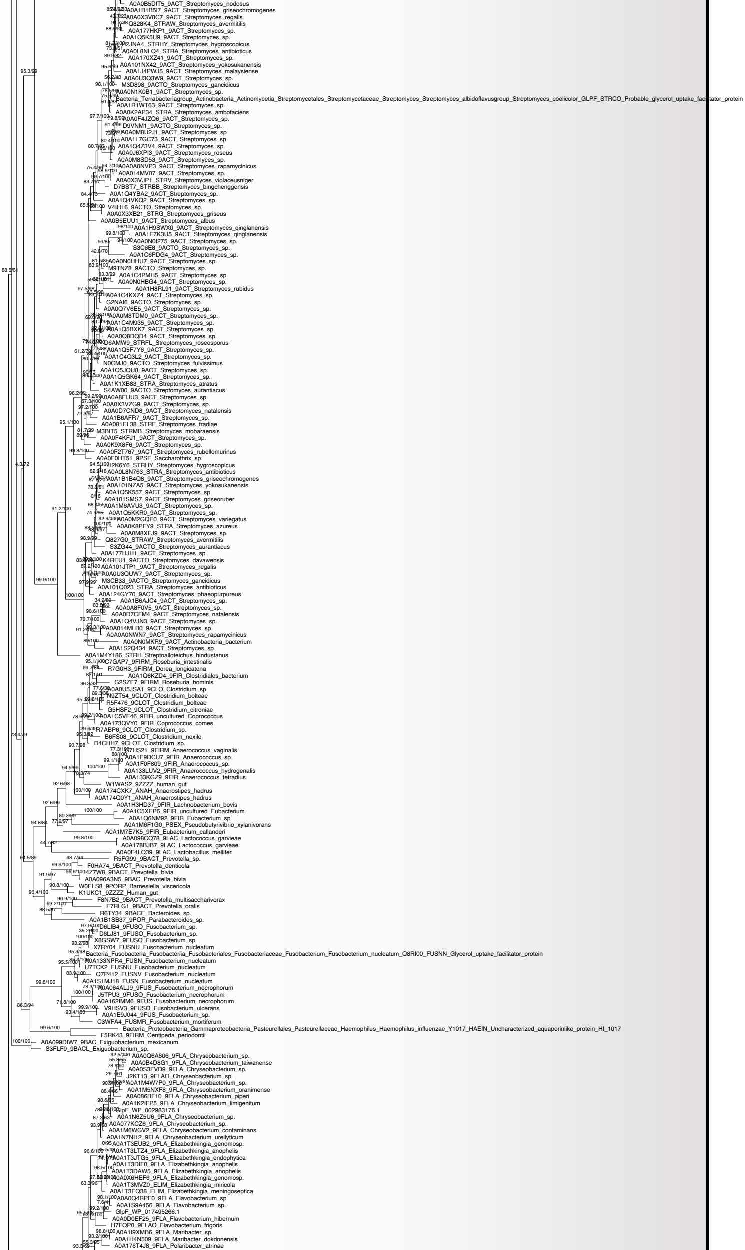

A0A0B5DIT5_9ACT_Streptomyces_nodosus
A0A1B1B5I7_9ACT_Streptomyces_griseochromogenes
A0A0X3V8C7_9ACT_Streptomyces_regalis
Q828K4_STRAW_Streptomyces_avermitilis
A0A177HKP1_9ACT_Streptomyces_sp.
A0A1Q5K5U9_9ACT_Streptomyces_sp.
A0A0L8NLQ4_STRA_Streptomyces_antibioticus
A0A170XZ41_9ACT_Streptomyces_sp.
A0A101NX42_9ACT_Streptomyces_yokosukanensis
A0A1J4PWJ5_9ACT_Streptomyces_malaysiense
A0A0U3Q3W9_9ACT_Streptomyces_sp.
M3D898_9ACTO_Streptomyces_gancidicus
A0A0N1K0B1_9ACT_Streptomyces_sp.
Bacteria_Terrabacteriagroup_Actinobacteria_Actinomycetia_Streptomycetales_Streptomycetaceae_Streptomyces_Streptomyces_albidoflavusgroup_Streptomyces_coelicolor_GLPF_STRCO_Probable_glycerol_uptake_facilitator_protein
A0A1R1WT63_9ACT_Streptomyces_sp.
A0A0K2AP34_STRA_Streptomyces_ambofaciens
A0A0F4JZQ6_9ACT_Streptomyces_sp.
D9VNM1_9ACTO_Streptomyces_sp.
A0A0M8U2J1_9ACT_Streptomyces_sp.
A0A1L7GC73_9ACT_Streptomyces_sp.
A0A1Q4Z3V4_9ACT_Streptomyces_sp.
A0A0J6XPI3_9ACT_Streptomyces_roseus
A0A0M8SD53_9ACT_Streptomyces_sp.
A0A0A0NVP3_9ACT_Streptomyces_rapamycinicus
A0A014MV07_9ACT_Streptomyces_sp.
A0A0X3VJP1_STRV_Streptomyces_violaceusniger
D7BST7_STRBB_Streptomyces_bingchenggensis
A0A1Q4YBA2_9ACT_Streptomyces_sp.
A0A1Q4VKQ2_9ACT_Streptomyces_sp.
V4IH16_9ACTO_Streptomyces_sp.
A0A0X3XB21_STRG_Streptomyces_griseus
A0A0B5EUU1_9ACT_Streptomyces_albus
A0A1H9SWX0_9ACT_Streptomyces_qinglanensis
A0A1E7K3U5_9ACT_Streptomyces_qinglanensis
A0A0N0I275_9ACT_Streptomyces_sp.
S3C6E8_9ACTO_Streptomyces_sp.
A0A1C6PDG4_9ACT_Streptomyces_sp.
A0A0N0HHU7_9ACT_Streptomyces_sp.
M9TNZ8_9ACTO_Streptomyces_sp.
A0A1C4PMH5_9ACT_Streptomyces_sp.
A0A0N0HBG4_9ACT_Streptomyces_sp.
A0A1H8RL91_9ACT_Streptomyces_rubidus
A0A1C4KXZ4_9ACT_Streptomyces_sp.
G2NAI6_9ACTO_Streptomyces_sp.
A0A0Q7V6E5_9ACT_Streptomyces_sp.
A0A0M8TDM0_9ACT_Streptomyces_sp.
A0A1C4M935_9ACT_Streptomyces_sp.
A0A1Q5BXK7_9ACT_Streptomyces_sp.
A0A0Q8DQD4_9ACT_Streptomyces_sp.
D6AMW9_STRFL_Streptomyces_roseosporus
A0A1Q5F7Y6_9ACT_Streptomyces_sp.
A0A1C4Q3L2_9ACT_Streptomyces_sp.
N0CMJ0_9ACTO_Streptomyces_fulvissimus
A0A1Q5JQU8_9ACT_Streptomyces_sp.
A0A1Q5GK64_9ACT_Streptomyces_sp.
A0A1K1XB83_STRA_Streptomyces_atratus
S4AW00_9ACTO_Streptomyces_aurantiacus
A0A0A8EUU3_9ACT_Streptomyces_sp.
A0A0X3VZG9_9ACT_Streptomyces_sp.
A0A0D7CND8_9ACT_Streptomyces_natalensis
A0A1B6AFR7_9ACT_Streptomyces_sp.
A0A081EL38_STRF_Streptomyces_fradiae
M3BIT5_STRMB_Streptomyces_mobaraensis
A0A0F4KFJ1_9ACT_Streptomyces_sp.
A0A0K9X8F6_9ACT_Streptomyces_sp.
A0A0F2T767_9ACT_Streptomyces_rubellomurinus
A0A0F0HT51_9PSE_Saccharothrix_sp.
H2K6Y6_STRHY_Streptomyces_hygroscopicus
A0A0L8N763_STRA_Streptomyces_antibioticus
A0A1B1B4Q8_9ACT_Streptomyces_griseochromogenes
A0A101NZA5_9ACT_Streptomyces_yokosukanensis
A0A1Q5K557_9ACT_Streptomyces_sp.
A0A101SMS7_9ACT_Streptomyces_griseoruber
A0A1M6AVU3_9ACT_Streptomyces_sp.
A0A1Q5KKR0_9ACT_Streptomyces_sp.
A0A0M2GQE0_9ACT_Streptomyces_variegatus
A0A0K8PFY9_STRA_Streptomyces_azureus
A0A0M8XFJ9_9ACT_Streptomyces_sp.
Q827G0_STRAW_Streptomyces_avermitilis
S3ZG44_9ACTO_Streptomyces_aurantiacus
A0A177HJH1_9ACT_Streptomyces_sp.
K4REU1_9ACTO_Streptomyces_davawensis
A0A101JTP1_9ACT_Streptomyces_regalis
A0A0U3QUW7_9ACT_Streptomyces_sp.
M3CB33_9ACTO_Streptomyces_gancidicus
A0A101Q023_STRA_Streptomyces_antibioticus
A0A124GY70_9ACT_Streptomyces_phaeopurpureus
A0A1B6AJC4_9ACT_Streptomyces_sp.
A0A0A8F0V5_9ACT_Streptomyces_sp.
A0A0D7CFM4_9ACT_Streptomyces_natalensis
A0A1Q4VJN3_9ACT_Streptomyces_sp.
A0A014MLB0_9ACT_Streptomyces_sp.
A0A0A0NWN7_9ACT_Streptomyces_rapamycinicus
A0A0N0MKR9_9ACT_Actinobacteria_bacterium
A0A1S2Q434_9ACT_Streptomyces_sp.
A0A1M4Y186_STRH_Streptoalloteichus_hindustanus
C7GAP7_9FIRM_Roseburia_intestinalis
R7G0H3_9FIRM_Dorea_longicatena
A0A1Q6KZD4_9FIR_Clostridiales_bacterium
G2SZE7_9FIRM_Roseburia_hominis
A0A0U5JSA1_9CLO_Clostridium_sp.
N9ZT54_9CLOT_Clostridium_bolteae
R5F476_9CLOT_Clostridium_bolteae
G5HSF2_9CLOT_Clostridium_citroniae
A0A1C5VE46_9FIR_uncultured_Coprococcus
A0A173QVY0_9FIR_Coprococcus_comes
R7ABP6_9CLOT_Clostridium_sp.
B6FS08_9CLOT_Clostridium_nexile
D4CHH7_9CLOT_Clostridium_sp.
D7HS21_9FIRM_Anaerococcus_vaginalis
A0A1E9DCU7_9FIR_Anaerococcus_sp.
A0A1F0F809_9FIR_Anaerococcus_sp.
A0A133LUV2_9FIR_Anaerococcus_hydrogenalis
A0A133KGZ9_9FIR_Anaerococcus_tetradius
W1WAS2_9ZZZZ_human_gut
A0A174CXK7_ANAH_Anaerostipes_hadrus
A0A174Q0Y1_ANAH_Anaerostipes_hadrus
A0A1H3HD37_9FIR_Lachnobacterium_bovis
A0A1C5XEP6_9FIR_uncultured_Eubacterium
A0A1Q6NM92_9FIR_Eubacterium_sp.
A0A1M6F1G0_PSEX_Pseudobutyrivibrio_xylanivorans
A0A1M7E7K5_9FIR_Eubacterium_callanderi
A0A098CQ78_9LAC_Lactococcus_garvieae
A0A178BJB7_9LAC_Lactococcus_garvieae
A0A0F4LQ39_9LAC_Lactobacillus_mellifer
R5FG99_9BACT_Prevotella_sp.
F0HA74_9BACT_Prevotella_denticola
I4Z7W8_9BACT_Prevotella_bivia
A0A096A3N5_9BAC_Prevotella_bivia
W0ELS8_9PORP_Barnesiella_viscericola
K1UKC1_9ZZZZ_Human_gut
F8N7B2_9BACT_Prevotella_multisaccharivorax
E7RLG1_9BACT_Prevotella_oralis
R6TY34_9BACE_Bacteroides_sp.
A0A1B1SB37_9POR_Parabacteroides_sp.
D6LIB4_9FUSO_Fusobacterium_sp.
D6LJ81_9FUSO_Fusobacterium_sp.
X8GSW7_9FUSO_Fusobacterium_sp.
X7RY04_FUSNU_Fusobacterium_nucleatum
Bacteria_Fusobacteria_Fusobacteriia_Fusobacteriales_Fusobacteriaceae_Fusobacterium_Fusobacterium_nucleatum_Q8RI00_FUSNN_Glycerol_uptake_facilitator_protein
A0A133NPR4_FUSN_Fusobacterium_nucleatum
U7TCK2_FUSNU_Fusobacterium_nucleatum
Q7P412_FUSNV_Fusobacterium_nucleatum
A0A1S1MJ18_FUSN_Fusobacterium_nucleatum
A0A064ALJ9_9FUS_Fusobacterium_necrophorum
J5TPU3_9FUSO_Fusobacterium_necrophorum
A0A162IMM6_9FUS_Fusobacterium_necrophorum
V9HSV3_9FUSO_Fusobacterium_ulcerans
A0A1E9J044_9FUS_Fusobacterium_sp.
C3WFA4_FUSMR_Fusobacterium_mortiferum
Bacteria_Proteobacteria_Gammaproteobacteria_Pasteurellales_Pasteurellaceae_Haemophilus_Haemophilus_influenzae_Y1017_HAEIN_Uncharacterized_aquaporinlike_protein_HI_1017
F5RK43_9FIRM_Centipeda_periodontii
A0A099DIW7_9BAC_Exiguobacterium_mexicanum
S3FLF9_9BACL_Exiguobacterium_sp.
A0A0Q6A806_9FLA_Chryseobacterium_sp.
A0A0B4D8G1_9FLA_Chryseobacterium_taiwanense
A0A0S3FVD9_9FLA_Chryseobacterium_sp.
J2KT13_9FLAO_Chryseobacterium_sp.
A0A1M4W7P0_9FLA_Chryseobacterium_sp.
A0A1M5NXF8_9FLA_Chryseobacterium_oranimense
A0A086BF10_9FLA_Chryseobacterium_piperi
A0A1K2IFP5_9FLA_Chryseobacterium_limigenitum
GlpF_WP_002983176.1
A0A1N6Z5U6_9FLA_Chryseobacterium_sp.
A0A077KCZ6_9FLA_Chryseobacterium_sp.
A0A1M6WGV2_9FLA_Chryseobacterium_contaminans
A0A1N7NI12_9FLA_Chryseobacterium_ureilyticum
A0A1T3EUB2_9FLA_Elizabethkingia_genomosp.
A0A1T3LTZ4_9FLA_Elizabethkingia_anophelis
A0A1T3JTG5_9FLA_Elizabethkingia_endophytica
A0A1T3DIF0_9FLA_Elizabethkingia_anophelis
A0A1T3DAW5_9FLA_Elizabethkingia_anophelis
A0A0X6HEF6_9FLA_Elizabethkingia_genomosp.
A0A1T3MVZ0_ELIM_Elizabethkingia_miricola
A0A1T3EQ38_ELIM_Elizabethkingia_meningoseptica
A0A0Q4RPF0_9FLA_Flavobacterium_sp.
A0A1S9A456_9FLA_Flavobacterium_sp.
GlpF_WP_017495266.1
A0A0D0EF25_9FLA_Flavobacterium_hibernum
H7FQP0_9FLAO_Flavobacterium_frigoris
A0A1I9XMB6_9FLA_Maribacter_sp.
A0A1H4N509_9FLA_Maribacter_dokdonensis
A0A176T4J8_9FLA_Polaribacter_atrinae

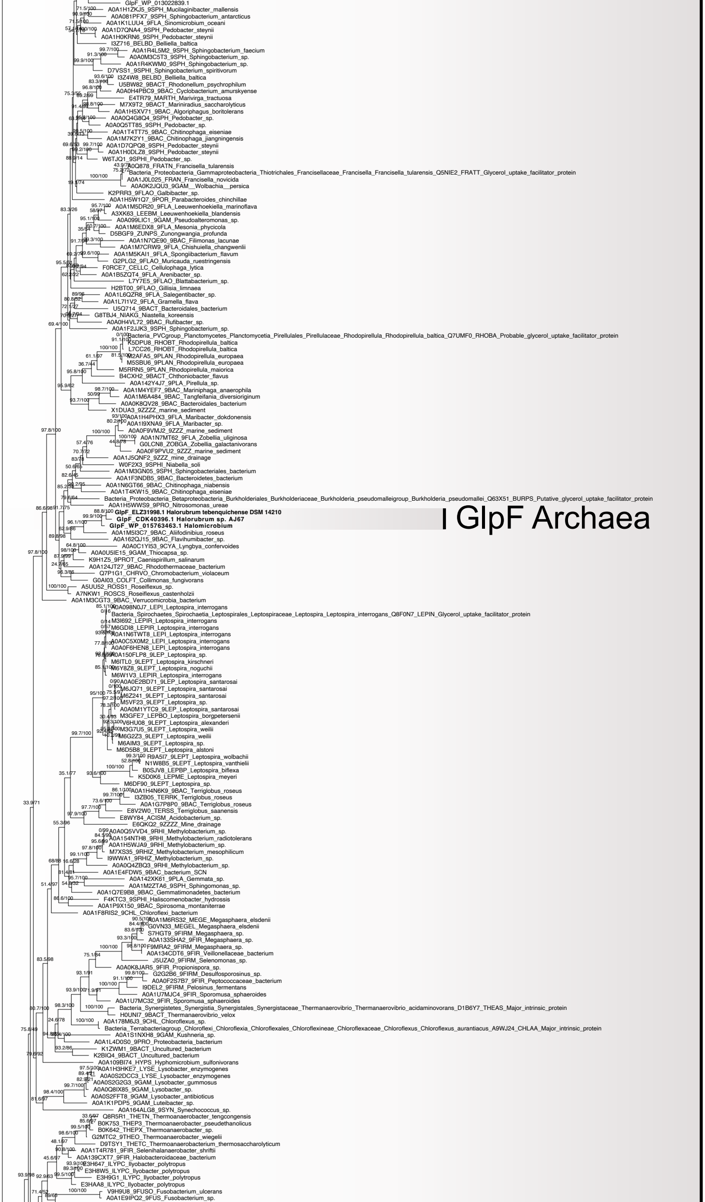
GlpF_ELZ31998.1 Halorubrum tebenquichense DSM 14210
GlpF_CDK40396.1 Halorubrum sp. AJ67
GlpF_WP_015763463.1 Halomicrobium
GlpF Archaea

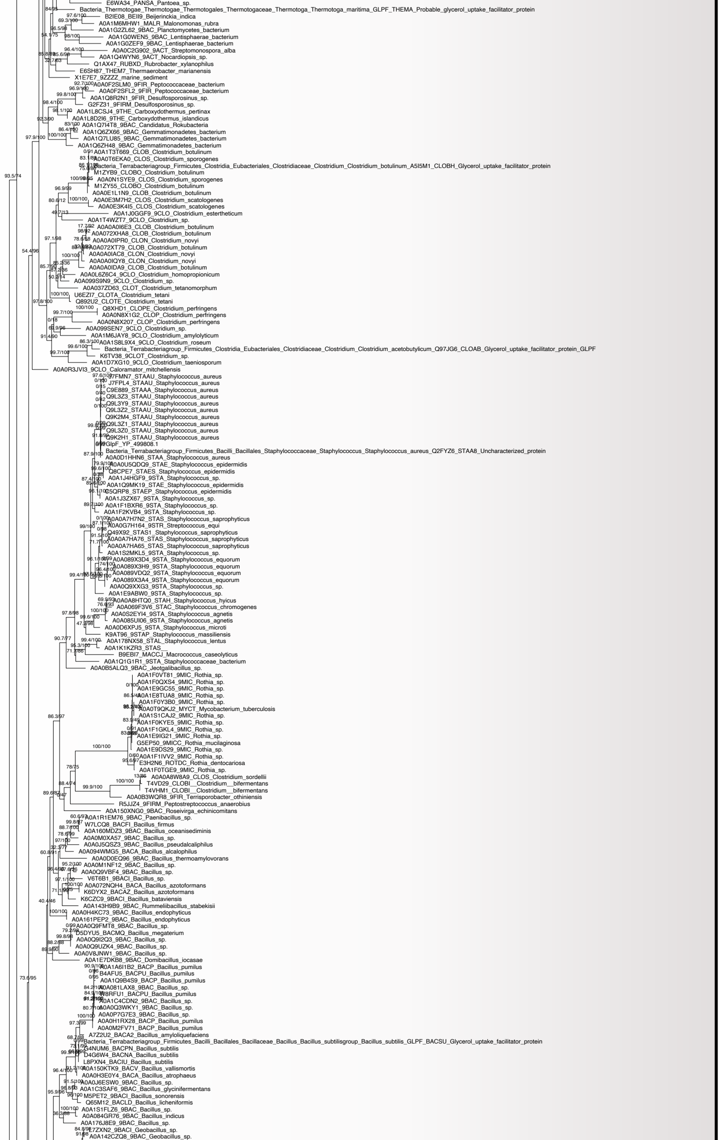
E6WA34_PANSA_Pantoea_sp.
Bacteria_Thermotogae_Thermotogae_Thermotogales_Thermotogaceae_Thermotoga_Thermotoga_maritima_GLPF_THEMA_Probable_glycerol_uptake_facilitator_protein
B2IE08_BEII9_Beijerinckia_indica
A0A1M6MHW1_MALR_Malonomonas_rubra
A0A1G2ZL62_9BAC_Planctomycetes_bacterium
A0A1G0WEN5_9BAC_Lentisphaerae_bacterium
A0A1G0ZEF9_9BAC_Lentisphaerae_bacterium
A0A0C2G902_9ACT_Streptomonospora_alba
A0A1Q4WYN6_9ACT_Nocardiopsis_sp.
Q1AX47_RUBXD_Rubrobacter_xylanophilus
E6SH87_THEM7_Thermaerobacter_marianensis
X1E7E7_9ZZZZ_marine_sediment
A0A0F2SLM0_9FIR_Peptococcaceae_bacterium
A0A0F2SFL2_9FIR_Peptococcaceae_bacterium
A0A1Q8R2N1_9FIR_Desulfosporosinus_sp.
G2FZ31_9FIRM_Desulfosporosinus_sp.
A0A1L8CSJ4_9THE_Carboxydothermus_pertinax
A0A1L8D2I6_9THE_Carboxydothermus_islandicus
A0A1Q7I4T8_9BAC_Candidatus_Rokubacteria
A0A1Q6ZX66_9BAC_Gemmatimonadetes_bacterium
A0A1Q7LU85_9BAC_Gemmatimonadetes_bacterium
A0A1Q6ZH48_9BAC_Gemmatimonadetes_bacterium
A0A1T3T669_CLOB_Clostridium_botulinum
A0A0T6EKA0_CLOS_Clostridium_sporogenes
Bacteria_Terrabacteriagroup_Firmicutes_Clostridia_Eubacteriales_Clostridiaceae_Clostridium_Clostridium_botulinum_A5I5M1_CLOBH_Glycerol_uptake_facilitator_protein
M1ZYB9_CLOBO_Clostridium_botulinum
A0A0N1SYE9_CLOS_Clostridium_sporogenes
M1ZY55_CLOBO_Clostridium_botulinum
A0A0E1L1N9_CLOB_Clostridium_botulinum
A0A0E3M7H2_CLOS_Clostridium_scatologenes
A0A0E3K4I5_CLOS_Clostridium_scatologenes
A0A1J0GGF9_9CLO_Clostridium_estertheticum
A0A1T4WZT7_9CLO_Clostridium_sp.
A0A0A0I6E3_CLOB_Clostridium_botulinum
A0A072XHA8_CLOB_Clostridium_botulinum
A0A0A0IPR0_CLON_Clostridium_novyi
A0A072XT79_CLOB_Clostridium_botulinum
A0A0A0IAC8_CLON_Clostridium_novyi
A0A0A0IQY8_CLON_Clostridium_novyi
A0A0A0IDA9_CLOB_Clostridium_botulinum
A0A0L6Z6C4_9CLO_Clostridium_homopropionicum
A0A099S9N9_9CLO_Clostridium_sp.
A0A037ZD63_CLOT_Clostridium_tetanomorphum
U6EZI7_CLOTA_Clostridium_tetani
Q892U2_CLOTE_Clostridium_tetani
Q8XHD1_CLOPE_Clostridium_perfringens
A0A0N8X1G2_CLOP_Clostridium_perfringens
A0A0N8X207_CLOP_Clostridium_perfringens
A0A099SEN7_9CLO_Clostridium_sp.
A0A1M6JAY8_9CLO_Clostridium_amylolyticum
A0A1S8L9X4_9CLO_Clostridium_roseum
Bacteria_Terrabacteriagroup_Firmicutes_Clostridia_Eubacteriales_Clostridiaceae_Clostridium_Clostridium_acetobutylicum_Q97JG6_CLOAB_Glycerol_uptake_facilitator_protein_GLPF
K6TV38_9CLOT_Clostridium_sp.
A0A1D7XG10_9CLO_Clostridium_taeniosporum
A0A0R3JVI3_9CLO_Caloramator_mitchellensis
J7FMN7_STAAU_Staphylococcus_aureus
J7FPL4_STAAU_Staphylococcus_aureus
C9E889_STAAA_Staphylococcus_aureus
Q9L3Z3_STAAU_Staphylococcus_aureus
Q9L3Y9_STAAU_Staphylococcus_aureus
Q9L3Z2_STAAU_Staphylococcus_aureus
Q9K2M4_STAAU_Staphylococcus_aureus
Q9L3Z1_STAAU_Staphylococcus_aureus
Q9L3Z0_STAAU_Staphylococcus_aureus
Q9K2H1_STAAU_Staphylococcus_aureus
GlpF_YP_499808.1
Bacteria_Terrabacteriagroup_Firmicutes_Bacilli_Bacillales_Staphylococcaceae_Staphylococcus_Staphylococcus_aureus_Q2FYZ6_STAA8_Uncharacterized_protein
A0A0D1HHN6_STAA_Staphylococcus_aureus
A0A0U5QDQ9_STAE_Staphylococcus_epidermidis
Q8CPE7_STAES_Staphylococcus_epidermidis
A0A1J4HGF9_9STA_Staphylococcus_sp.
A0A1Q9MK19_STAE_Staphylococcus_epidermidis
Q5QRP8_STAEP_Staphylococcus_epidermidis
A0A1J3ZX67_9STA_Staphylococcus_sp.
A0A1F1BXR6_9STA_Staphylococcus_sp.
A0A1F2KVB4_9STA_Staphylococcus_sp.
A0A0A7H7N2_STAS_Staphylococcus_saprophyticus
A0A0G7H164_9STR_Streptococcus_equi
Q49X92_STAS1_Staphylococcus_saprophyticus
A0A0A7HA76_STAS_Staphylococcus_saprophyticus
A0A0A7HA65_STAS_Staphylococcus_saprophyticus
A0A1S2MKL5_9STA_Staphylococcus_sp.
A0A089X3D4_9STA_Staphylococcus_equorum
A0A089X3H9_9STA_Staphylococcus_equorum
A0A089VDQ2_9STA_Staphylococcus_equorum
A0A089X3A4_9STA_Staphylococcus_equorum
A0A0Q9XXG3_9STA_Staphylococcus_sp.
A0A1E9ABW0_9STA_Staphylococcus_sp.
A0A0A8HTQ0_STAH_Staphylococcus_hyicus
A0A069F3V6_STAC_Staphylococcus_chromogenes
A0A0S2EYI4_9STA_Staphylococcus_agnetis
A0A085UI06_9STA_Staphylococcus_agnetis
A0A0D6XPJ5_9STA_Staphylococcus_microti
K9AT96_9STAP_Staphylococcus_massiliensis
A0A178NX58_STAL_Staphylococcus_lentus
A0A1K1KZR3_STAS__
B9EBI7_MACCJ_Macrococcus_caseolyticus
A0A1Q1G1R1_9STA_Staphylococcaceae_bacterium
A0A0B5ALQ3_9BAC_Jeotgalibacillus_sp.
A0A1F0VT81_9MIC_Rothia_sp.
A0A1F0QXS4_9MIC_Rothia_sp.
A0A1E9GC55_9MIC_Rothia_sp.
A0A1E8TUA8_9MIC_Rothia_sp.
A0A1F0Y3B0_9MIC_Rothia_sp.
A0A0T9QKJ2_MYCT_Mycobacterium_tuberculosis
A0A1S1CAJ2_9MIC_Rothia_sp.
A0A1F0KYE5_9MIC_Rothia_sp.
A0A1F1GKL4_9MIC_Rothia_sp.
A0A1E9IG21_9MIC_Rothia_sp.
G5EP50_9MICC_Rothia_mucilaginosa
A0A1E9DS29_9MIC_Rothia_sp.
A0A1F1IVV2_9MIC_Rothia_sp.
E3H2N6_ROTDC_Rothia_dentocariosa
A0A1F0TGE9_9MIC_Rothia_sp.
A0A0A8W8A9_CLOS_Clostridium_sordellii
T4VD29_CLOBI__Clostridium__bifermentans
T4VHM1_CLOBI__Clostridium__bifermentans
A0A0B3WQR8_9FIR_Terrisporobacter_othiniensis
R5JJZ4_9FIRM_Peptostreptococcus_anaerobius
A0A150XNG0_9BAC_Roseivirga_echinicomitans
A0A1R1EM76_9BAC_Paenibacillus_sp.
W7LCQ8_BACFI_Bacillus_firmus
A0A160MDZ3_9BAC_Bacillus_oceanisediminis
A0A0M0XA57_9BAC_Bacillus_sp.
A0A0J5QSZ3_9BAC_Bacillus_pseudalcaliphilus
A0A094WMG5_BACA_Bacillus_alcalophilus
A0A0D0EQ96_9BAC_Bacillus_thermoamylovorans
A0A0M1NF12_9BAC_Bacillus_sp.
A0A0Q9VBF4_9BAC_Bacillus_sp.
V6T6B1_9BACI_Bacillus_sp.
A0A072NQH4_BACA_Bacillus_azotoformans
K6DYX2_BACAZ_Bacillus_azotoformans
K6CZC9_9BACI_Bacillus_bataviensis
A0A143H9B9_9BAC_Rummeliibacillus_stabekisii
A0A0H4KC73_9BAC_Bacillus_endophyticus
A0A161PEP2_9BAC_Bacillus_endophyticus
A0A0Q9FMT8_9BAC_Bacillus_sp.
D5DYU5_BACMQ_Bacillus_megaterium
A0A0Q9I2Q3_9BAC_Bacillus_sp.
A0A0Q9UZK4_9BAC_Bacillus_sp.
A0A0V8JNW1_9BAC_Bacillus_sp.
A0A1E7DKB8_9BAC_Domibacillus_iocasae
A0A1A6I1B2_BACP_Bacillus_pumilus
B4AFU5_BACPU_Bacillus_pumilus
A0A1Q9B4S9_BACP_Bacillus_pumilus
A0A081LAX8_9BAC_Bacillus_sp.
W8RFU1_BACPU_Bacillus_pumilus
A0A1C4CDN2_9BAC_Bacillus_sp.
A0A0Q3WKY1_9BAC_Bacillus_sp.
A0A0P7G7E3_9BAC_Bacillus_sp.
A0A0H1RX28_BACP_Bacillus_pumilus
A0A0M2FV71_BACP_Bacillus_pumilus
A7Z2U2_BACA2_Bacillus_amyloliquefaciens
Bacteria_Terrabacteriagroup_Firmicutes_Bacilli_Bacillales_Bacillaceae_Bacillus_Bacillus_subtilisgroup_Bacillus_subtilis_GLPF_BACSU_Glycerol_uptake_facilitator_protein
G4NUM6_BACPN_Bacillus_subtilis
D4G6W4_BACNA_Bacillus_subtilis
L8PXN4_BACIU_Bacillus_subtilis
A0A150KTK9_BACV_Bacillus_vallismortis
A0A0H3E0Y4_BACA_Bacillus_atrophaeus
A0A0J6ESW0_9BAC_Bacillus_sp.
A0A1C3SAF6_9BAC_Bacillus_glycinifermentans
M5PET2_9BACI_Bacillus_sonorensis
Q65M12_BACLD_Bacillus_licheniformis
A0A1S1FLZ6_9BAC_Bacillus_sp.
A0A084GR76_9BAC_Bacillus_indicus
A0A176J8E9_9BAC_Bacillus_sp.
E7ZXN2_9BACI_Geobacillus_sp.
A0A142CZQ8_9BAC_Geobacillus_sp.

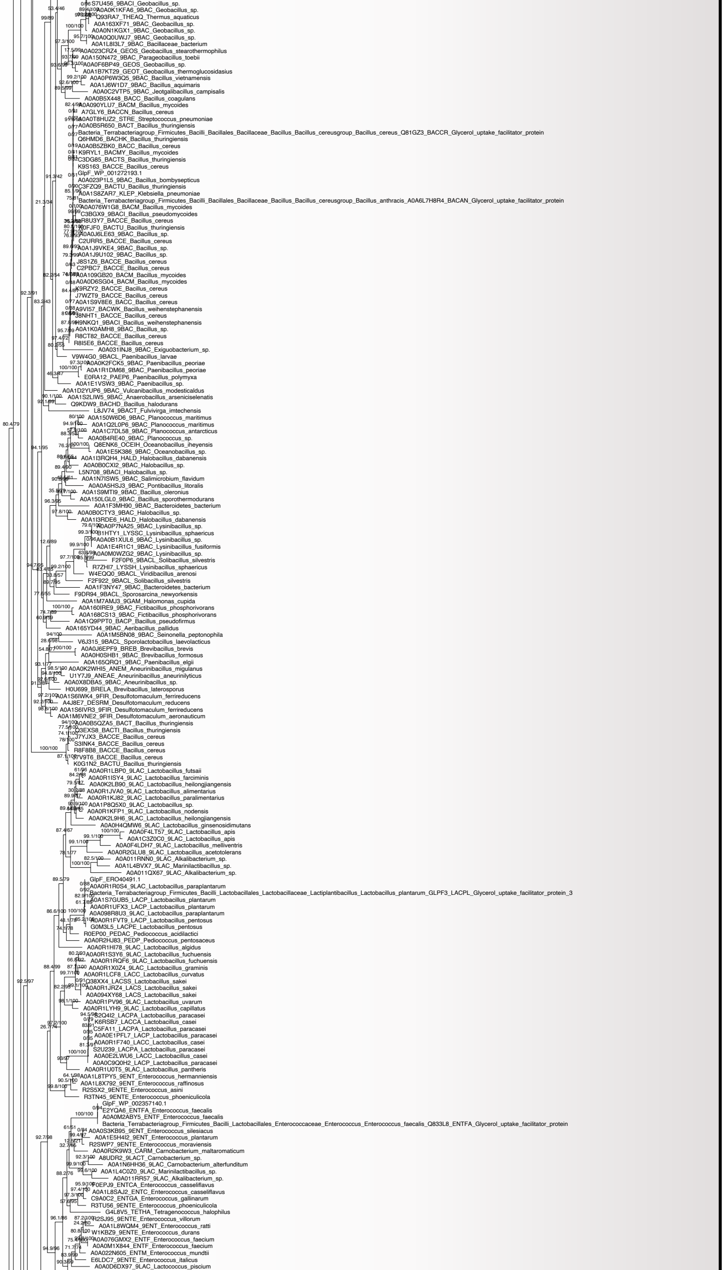
S7U456_9BACI_Geobacillus_sp.
A0A0K1KFA6_9BAC_Geobacillus_sp.
Q93RA7_THEAQ_Thermus_aquaticus
A0A163XF71_9BAC_Geobacillus_sp.
A0A0N1KGX1_9BAC_Geobacillus_sp.
A0A0Q0UWJ7_9BAC_Geobacillus_sp.
A0A1L8I3L7_9BAC_Bacillaceae_bacterium
A0A023CRZ4_GEOS_Geobacillus_stearothermophilus
A0A150N472_9BAC_Parageobacillus_toebii
A0A0F6BP49_GEOS_Geobacillus_sp.
A0A1B7KT29_GEOT_Geobacillus_thermoglucosidasius
A0A0P6W3Q5_9BAC_Bacillus_vietnamensis
A0A1J6W1D7_9BAC_Bacillus_aquimaris
A0A0C2VTP5_9BAC_Jeotgalibacillus_campisalis
A0A0B5X448_BACC_Bacillus_coagulans
A0A090YLU7_BACM_Bacillus_mycoides
A7GLY6_BACCN_Bacillus_cereus
A0A0T8HUZ2_STRE_Streptococcus_pneumoniae
A0A0B5R650_BACT_Bacillus_thuringiensis
Bacteria_Terrabacteriagroup_Firmicutes_Bacilli_Bacillales_Bacillaceae_Bacillus_Bacillus_cereusgroup_Bacillus_cereus_Q81GZ3_BACCR_Glycerol_uptake_facilitator_protein
Q6HMD6_BACHK_Bacillus_thuringiensis
A0A0B5ZBK0_BACC_Bacillus_cereus
K9RYL1_BACMY_Bacillus_mycoides
C3DG85_BACTS_Bacillus_thuringiensis
K9S163_BACCE_Bacillus_cereus
GlpF_WP_001272193.1
A0A023P1L5_9BAC_Bacillus_bombysepticus
C3FZQ9_BACTU_Bacillus_thuringiensis
A0A1S8ZAR7_KLEP_Klebsiella_pneumoniae
Bacteria_Terrabacteriagroup_Firmicutes_Bacilli_Bacillales_Bacillaceae_Bacillus_Bacillus_cereusgroup_Bacillus_anthracis_A0A6L7H8R4_BACAN_Glycerol_uptake_facilitator_protein
A0A076W1G8_BACM_Bacillus_mycoides
C3BGX9_9BACI_Bacillus_pseudomycoides
R8U3Y7_BACCE_Bacillus_cereus
A0FJF0_BACTU_Bacillus_thuringiensis
A0A0J6LE63_9BAC_Bacillus_sp.
C2URR5_BACCE_Bacillus_cereus
A0A1J9VKE4_9BAC_Bacillus_sp.
A0A1J9U102_9BAC_Bacillus_sp.
J8S1Z6_BACCE_Bacillus_cereus
C2PBC7_BACCE_Bacillus_cereus
A0A109GB20_BACM_Bacillus_mycoides
A0A0D6SG04_BACM_Bacillus_mycoides
K9RZY2_BACCE_Bacillus_cereus
J7WZT9_BACCE_Bacillus_cereus
A0A1S9V8E6_BACC_Bacillus_cereus
A9VI57_BACWK_Bacillus_weihenstephanensis
J8NHT1_BACCE_Bacillus_cereus
H9NKQ1_9BACI_Bacillus_weihenstephanensis
A0A1K0AMH8_9BAC_Bacillus_sp.
R8CT82_BACCE_Bacillus_cereus
R8I5E6_BACCE_Bacillus_cereus
A0A031INJ8_9BAC_Exiguobacterium_sp.
V9W4G0_9BACL_Paenibacillus_larvae
A0A0K2FCK5_9BAC_Paenibacillus_peoriae
A0A1R1DM68_9BAC_Paenibacillus_peoriae
E0RA12_PAEP6_Paenibacillus_polymyxa
A0A1E1VSW3_9BAC_Paenibacillus_sp.
A0A1D2YUP6_9BAC_Vulcanibacillus_modesticaldus
A0A1S2LIW5_9BAC_Anaerobacillus_arseniciselenatis
Q9KDW9_BACHD_Bacillus_halodurans
L8JV74_9BACT_Fulvivirga_imtechensis
A0A150W6D6_9BAC_Planococcus_maritimus
A0A1Q2L0P6_9BAC_Planococcus_maritimus
A0A1C7DL58_9BAC_Planococcus_antarcticus
A0A0B4RE40_9BAC_Planococcus_sp.
Q8ENK6_OCEIH_Oceanobacillus_iheyensis
A0A1E5K386_9BAC_Oceanobacillus_sp.
A0A1I3RQH4_HALD_Halobacillus_dabanensis
A0A0B0CXI2_9BAC_Halobacillus_sp.
L5N708_9BACI_Halobacillus_sp.
A0A1N7ISW5_9BAC_Salimicrobium_flavidum
A0A0A5HSJ3_9BAC_Pontibacillus_litoralis
A0A1S9MTI9_9BAC_Bacillus_oleronius
A0A150LGL0_9BAC_Bacillus_sporothermodurans
A0A1F3MH90_9BAC_Bacteroidetes_bacterium
A0A0B0CTY3_9BAC_Halobacillus_sp.
A0A1I3RDE6_HALD_Halobacillus_dabanensis
A0A0P7NA25_9BAC_Lysinibacillus_sp.
B1HTY1_LYSSC_Lysinibacillus_sphaericus
A0A0B1XUL6_9BAC_Lysinibacillus_sp.
A0A1E4R1C1_9BAC_Lysinibacillus_fusiformis
A0A0M0WZG2_9BAC_Lysinibacillus_sp.
F2F0P6_9BACL_Solibacillus_silvestris
R7ZHI7_LYSSH_Lysinibacillus_sphaericus
W4EQQ0_9BACL_Viridibacillus_arenosi
F2F922_9BACL_Solibacillus_silvestris
A0A1F3NY47_9BAC_Bacteroidetes_bacterium
F9DR94_9BACL_Sporosarcina_newyorkensis
A0A1M7AMJ3_9GAM_Halomonas_cupida
A0A160IRE9_9BAC_Fictibacillus_phosphorivorans
A0A168CS13_9BAC_Fictibacillus_phosphorivorans
A0A1Q9PPT0_BACP_Bacillus_pseudofirmus
A0A165YD44_9BAC_Aeribacillus_pallidus
A0A1M5BN08_9BAC_Seinonella_peptonophila
V6J315_9BACL_Sporolactobacillus_laevolacticus
A0A0J6EPF9_BREB_Brevibacillus_brevis
A0A0H0SHB1_9BAC_Brevibacillus_formosus
A0A165QRQ1_9BAC_Paenibacillus_elgii
A0A0K2WHI5_ANEM_Aneurinibacillus_migulanus
U1Y7J9_ANEAE_Aneurinibacillus_aneurinilyticus
A0A0X8DBA5_9BAC_Aneurinibacillus_sp.
H0U699_BRELA_Brevibacillus_laterosporus
A0A1S6IWK4_9FIR_Desulfotomaculum_ferrireducens
A4J8E7_DESRM_Desulfotomaculum_reducens
A0A1S6IVR3_9FIR_Desulfotomaculum_ferrireducens
A0A1M6VNE2_9FIR_Desulfotomaculum_aeronauticum
A0A0B5QZA5_BACT_Bacillus_thuringiensis
Q3EXS8_BACTI_Bacillus_thuringiensis
J7YJX3_BACCE_Bacillus_cereus
S3INK4_BACCE_Bacillus_cereus
R8F8B8_BACCE_Bacillus_cereus
J7V9T6_BACCE_Bacillus_cereus
K0G1N2_BACTU_Bacillus_thuringiensis
A0A0R1LBP0_9LAC_Lactobacillus_futsaii
A0A0R1ISY4_9LAC_Lactobacillus_farciminis
A0A0K2LB90_9LAC_Lactobacillus_heilongjiangensis
A0A0R1JVA0_9LAC_Lactobacillus_alimentarius
A0A0R1KJ82_9LAC_Lactobacillus_paralimentarius
A0A1P8Q5X0_9LAC_Lactobacillus_sp.
A0A0R1KFP1_9LAC_Lactobacillus_nodensis
A0A0K2L9H6_9LAC_Lactobacillus_heilongjiangensis
A0A0H4QMW6_9LAC_Lactobacillus_ginsenosidimutans
A0A0F4LT57_9LAC_Lactobacillus_apis
A0A1C3Z0C0_9LAC_Lactobacillus_apis
A0A0F4LDH7_9LAC_Lactobacillus_melliventris
A0A0R2GLU8_9LAC_Lactobacillus_acetotolerans
A0A011RNN0_9LAC_Alkalibacterium_sp.
A0A1L4BVX7_9LAC_Marinilactibacillus_sp.
A0A011QX67_9LAC_Alkalibacterium_sp.
GlpF_ERO40491.1
A0A0R1R0S4_9LAC_Lactobacillus_paraplantarum
Bacteria_Terrabacteriagroup_Firmicutes_Bacilli_Lactobacillales_Lactobacillaceae_Lactiplantibacillus_Lactobacillus_plantarum_GLPF3_LACPL_Glycerol_uptake_facilitator_protein_3
A0A1S7GUB5_LACP_Lactobacillus_plantarum
A0A0R1UFX3_LACP_Lactobacillus_plantarum
A0A098R8U3_9LAC_Lactobacillus_paraplantarum
A0A0R1FVT9_LACP_Lactobacillus_pentosus
G0M3L5_LACPE_Lactobacillus_pentosus
R0EP00_PEDAC_Pediococcus_acidilactici
A0A0R2HJ83_PEDP_Pediococcus_pentosaceus
A0A0R1HI78_9LAC_Lactobacillus_algidus
A0A0R1S3Y6_9LAC_Lactobacillus_fuchuensis
A0A0R1RQF6_9LAC_Lactobacillus_fuchuensis
A0A0R1X0Z4_9LAC_Lactobacillus_graminis
A0A0R1LCF8_LACC_Lactobacillus_curvatus
Q38XX4_LACSS_Lactobacillus_sakei
A0A0R1JRZ4_LACS_Lactobacillus_sakei
A0A094XY68_LACS_Lactobacillus_sakei
A0A0R1PV96_9LAC_Lactobacillus_uvarum
A0A0R1LYH9_9LAC_Lactobacillus_capillatus
S2Q4I2_LACPA_Lactobacillus_paracasei
K6RSB7_LACCA_Lactobacillus_casei
C5FA11_LACPA_Lactobacillus_paracasei
A0A0E1PFL7_LACP_Lactobacillus_paracasei
A0A0R1F740_LACC_Lactobacillus_casei
S2U239_LACPA_Lactobacillus_paracasei
A0A0E2LWU6_LACC_Lactobacillus_casei
A0A0C9Q0H2_LACP_Lactobacillus_paracasei
A0A0R1U0T5_9LAC_Lactobacillus_pantheris
A0A1L8TPY5_9ENT_Enterococcus_hermanniensis
A0A1L8X792_9ENT_Enterococcus_raffinosus
R2S5X2_9ENTE_Enterococcus_asini
R3TN45_9ENTE_Enterococcus_phoeniculicola
GlpF_WP_002357140.1
E2YQA6_ENTFA_Enterococcus_faecalis
A0A0M2ABY5_ENTF_Enterococcus_faecalis
Bacteria_Terrabacteriagroup_Firmicutes_Bacilli_Lactobacillales_Enterococcaceae_Enterococcus_Enterococcus_faecalis_Q833L8_ENTFA_Glycerol_uptake_facilitator_protein
A0A0S3KB95_9ENT_Enterococcus_silesiacus
A0A1E5H4I2_9ENT_Enterococcus_plantarum
R2SWP7_9ENTE_Enterococcus_moraviensis
A0A0R2K9W3_CARM_Carnobacterium_maltaromaticum
A8UDR2_9LACT_Carnobacterium_sp.
A0A1N6HH36_9LAC_Carnobacterium_alterfunditum
A0A1L4C0Z0_9LAC_Marinilactibacillus_sp.
A0A011RR57_9LAC_Alkalibacterium_sp.
F0EPJ9_ENTCA_Enterococcus_casseliflavus
A0A1L8SAJ2_ENTC_Enterococcus_casseliflavus
C9A0C2_ENTGA_Enterococcus_gallinarum
R3TU56_9ENTE_Enterococcus_phoeniculicola
G4L8V5_TETHA_Tetragenococcus_halophilus
R2SJ95_9ENTE_Enterococcus_villorum
A0A1L8WQM4_9ENT_Enterococcus_ratti
W1KBZ9_9ENTE_Enterococcus_durans
A0A076GMX2_ENTF_Enterococcus_faecium
A0A0M1X844_ENTF_Enterococcus_faecium
A0A022N605_ENTM_Enterococcus_mundtii
E6LDC7_9ENTE_Enterococcus_italicus
A0A0D6DX97_9LAC_Lactococcus_piscium
GlpF Bacteria

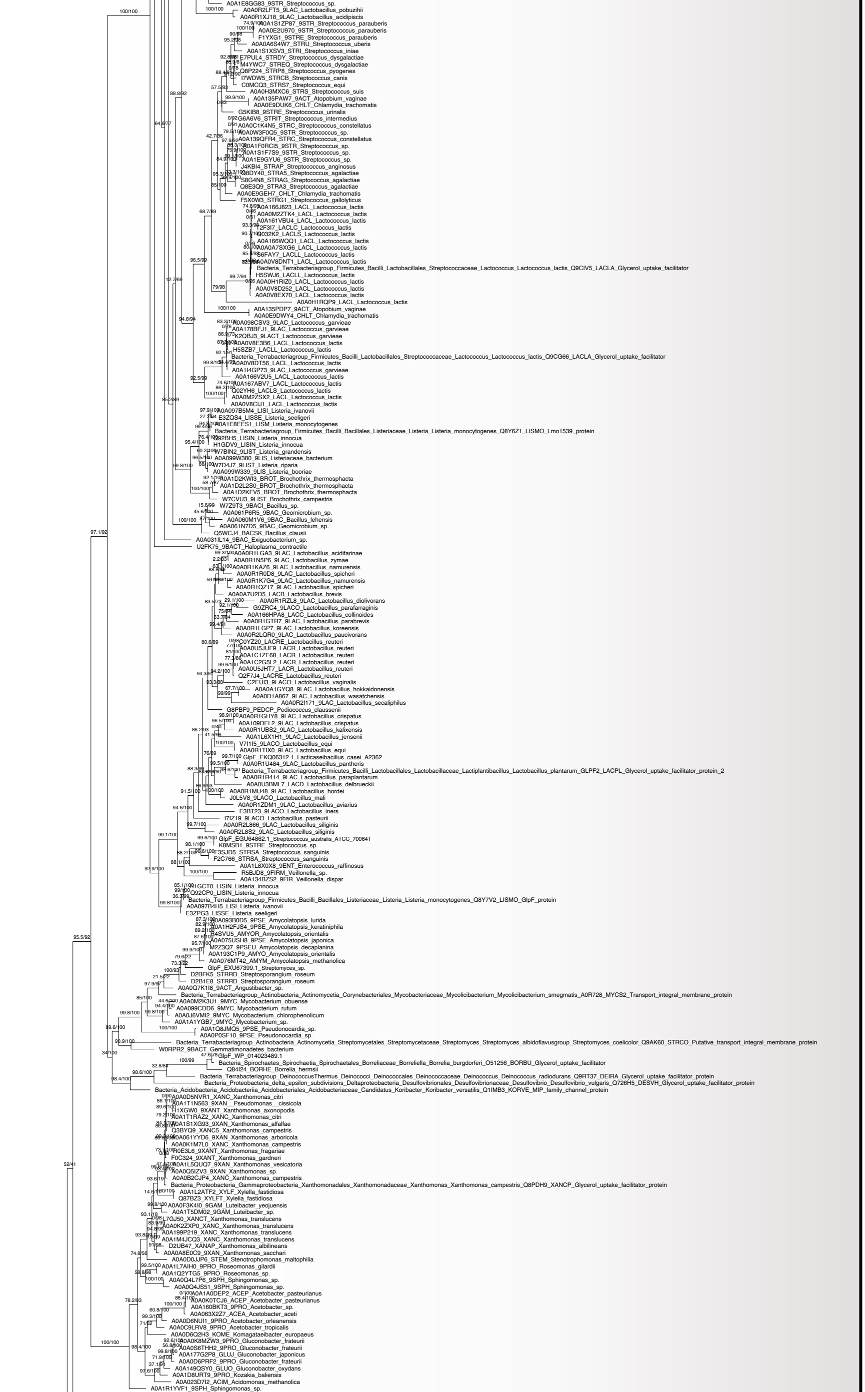
A0A1E8GG83_9STR_Streptococcus_sp.
A0A0R2LFT5_9LAC_Lactobacillus_pobuzihii
A0A0R1XJ18_9LAC_Lactobacillus_acidipiscis
A0A1S1ZP87_9STR_Streptococcus_parauberis
A0A0E2U970_9STR_Streptococcus_parauberis
F1YXG1_9STRE_Streptococcus_parauberis
A0A0A6S4W7_STRU_Streptococcus_uberis
A0A1S1XSV3_STRI_Streptococcus_iniae
E7PUL4_STRDY_Streptococcus_dysgalactiae
M4YWC7_STREQ_Streptococcus_dysgalactiae
Q8P224_STRP8_Streptococcus_pyogenes
I7WDW5_STRCB_Streptococcus_canis
C0MCQ3_STRS7_Streptococcus_equi
A0A0H3MXC6_STRS_Streptococcus_suis
A0A135PAW7_9ACT_Atopobium_vaginae
A0A0E9DUK6_CHLT_Chlamydia_trachomatis
G5KIB8_9STRE_Streptococcus_urinalis
G6A6V6_STRIT_Streptococcus_intermedius
A0A0C1K4N5_STRC_Streptococcus_constellatus
A0A0W3F0Q5_9STR_Streptococcus_sp.
A0A139QFR4_STRC_Streptococcus_constellatus
A0A1F0RCI5_9STR_Streptococcus_sp.
A0A1S1F7S9_9STR_Streptococcus_sp.
A0A1E9GYU6_9STR_Streptococcus_sp.
J4KBI4_STRAP_Streptococcus_anginosus
Q8DY40_STRA5_Streptococcus_agalactiae
S8G4N8_STRAG_Streptococcus_agalactiae
Q8E3Q9_STRA3_Streptococcus_agalactiae
A0A0E9GEH7_CHLT_Chlamydia_trachomatis
F5X0W3_STRG1_Streptococcus_gallolyticus
A0A166J823_LACL_Lactococcus_lactis
A0A0M2ZTK4_LACL_Lactococcus_lactis
A0A161VBU4_LACL_Lactococcus_lactis
T2F3I7_LACLC_Lactococcus_lactis
Q032K2_LACLS_Lactococcus_lactis
A0A166WQQ1_LACL_Lactococcus_lactis
A0A0A7SXG6_LACL_Lactococcus_lactis
S6FAY7_LACLL_Lactococcus_lactis
A0A0V8DNT1_LACL_Lactococcus_lactis
Bacteria_Terrabacteriagroup_Firmicutes_Bacilli_Lactobacillales_Streptococcaceae_Lactococcus_Lactococcus_lactis_Q9CIV5_LACLA_Glycerol_uptake_facilitator
H5SWJ6_LACLL_Lactococcus_lactis
A0A0H1RIZ0_LACL_Lactococcus_lactis
A0A0V8D252_LACL_Lactococcus_lactis
A0A0V8EX70_LACL_Lactococcus_lactis
A0A0H1RQP9_LACL_Lactococcus_lactis
A0A135PDP7_9ACT_Atopobium_vaginae
A0A0E9DWY4_CHLT_Chlamydia_trachomatis
A0A098CSV3_9LAC_Lactococcus_garvieae
A0A178BFJ1_9LAC_Lactococcus_garvieae
K2QBJ3_9LACT_Lactococcus_garvieae
A0A0V8E3B6_LACL_Lactococcus_lactis
H5SZB7_LACLL_Lactococcus_lactis
Bacteria_Terrabacteriagroup_Firmicutes_Bacilli_Lactobacillales_Streptococcaceae_Lactococcus_Lactococcus_lactis_Q9CG66_LACLA_Glycerol_uptake_facilitator
A0A0V8DT56_LACL_Lactococcus_lactis
A0A1I4GP73_9LAC_Lactococcus_garvieae
A0A166V2U5_LACL_Lactococcus_lactis
A0A167ABV7_LACL_Lactococcus_lactis
Q02YH6_LACLS_Lactococcus_lactis
A0A0M2ZSX2_LACL_Lactococcus_lactis
A0A0V8CIJ1_LACL_Lactococcus_lactis
A0A097B5M4_LISI_Listeria_ivanovii
E3ZQS4_LISSE_Listeria_seeligeri
A0A1E8EES1_LISM_Listeria_monocytogenes
Bacteria_Terrabacteriagroup_Firmicutes_Bacilli_Bacillales_Listeriaceae_Listeria_Listeria_monocytogenes_Q8Y6Z1_LISMO_Lmo1539_protein
Q92BH5_LISIN_Listeria_innocua
H1GDV9_LISIN_Listeria_innocua
W7BIN2_9LIST_Listeria_grandensis
A0A099W380_9LIS_Listeriaceae_bacterium
W7D4J7_9LIST_Listeria_riparia
A0A099W339_9LIS_Listeria_booriae
A0A1D2KWI3_BROT_Brochothrix_thermosphacta
A0A1D2L2S0_BROT_Brochothrix_thermosphacta
A0A1D2KFV5_BROT_Brochothrix_thermosphacta
W7CVU3_9LIST_Brochothrix_campestris
W7Z9T3_9BACI_Bacillus_sp.
A0A061P6R5_9BAC_Geomicrobium_sp.
A0A060M1V6_9BAC_Bacillus_lehensis
A0A061N7D5_9BAC_Geomicrobium_sp.
Q5WCJ4_BACSK_Bacillus_clausii
A0A031IL14_9BAC_Exiguobacterium_sp.
U2FK75_9BACT_Haloplasma_contractile
A0A0R1LGA3_9LAC_Lactobacillus_acidifarinae
A0A0R1N5P6_9LAC_Lactobacillus_zymae
A0A0R1KAZ6_9LAC_Lactobacillus_namurensis
A0A0R1R0D8_9LAC_Lactobacillus_spicheri
A0A0R1K7G4_9LAC_Lactobacillus_namurensis
A0A0R1QZ17_9LAC_Lactobacillus_spicheri
A0A0A7U2D5_LACB_Lactobacillus_brevis
A0A0R1RZL8_9LAC_Lactobacillus_diolivorans
G9ZRC4_9LACO_Lactobacillus_parafarraginis
A0A166HPA8_LACC_Lactobacillus_collinoides
A0A0R1GTR7_9LAC_Lactobacillus_parabrevis
A0A0R1LGP7_9LAC_Lactobacillus_koreensis
A0A0R2LQR0_9LAC_Lactobacillus_paucivorans
C0YZ20_LACRE_Lactobacillus_reuteri
A0A0U5JUF9_LACR_Lactobacillus_reuteri
A0A1C1ZE68_LACR_Lactobacillus_reuteri
A0A1C2G5L2_LACR_Lactobacillus_reuteri
A0A0U5JHT7_LACR_Lactobacillus_reuteri
Q2F7J4_LACRE_Lactobacillus_reuteri
C2EUI3_9LACO_Lactobacillus_vaginalis
A0A0A1GYQ8_9LAC_Lactobacillus_hokkaidonensis
A0A0D1A867_9LAC_Lactobacillus_wasatchensis
A0A0R2I171_9LAC_Lactobacillus_secaliphilus
G8PBF9_PEDCP_Pediococcus_claussenii
A0A0R1GHY8_9LAC_Lactobacillus_crispatus
A0A109DEL2_9LAC_Lactobacillus_crispatus
A0A0R1UBS2_9LAC_Lactobacillus_kalixensis
A0A1L6X1H1_9LAC_Lactobacillus_jensenii
V7I1I5_9LACO_Lactobacillus_equi
A0A0R1TIX0_9LAC_Lactobacillus_equi
GlpF_EKQ06312.1_Lacticaseibacillus_casei_A2362
A0A0R1U484_9LAC_Lactobacillus_pantheris
Bacteria_Terrabacteriagroup_Firmicutes_Bacilli_Lactobacillales_Lactobacillaceae_Lactiplantibacillus_Lactobacillus_plantarum_GLPF2_LACPL_Glycerol_uptake_facilitator_protein_2
A0A0R1R414_9LAC_Lactobacillus_paraplantarum
A0A0U3BML7_LACD_Lactobacillus_delbrueckii
A0A0R1MU48_9LAC_Lactobacillus_hordei
J0L5V8_9LACO_Lactobacillus_mali
A0A0R1ZDM1_9LAC_Lactobacillus_aviarius
E3BT23_9LACO_Lactobacillus_iners
I7IZ19_9LACO_Lactobacillus_pasteurii
A0A0R2L866_9LAC_Lactobacillus_siliginis
A0A0R2L8S2_9LAC_Lactobacillus_siliginis
GlpF_EGU64862.1_Streptococcus_australis_ATCC_700641
K8MSB1_9STRE_Streptococcus_sp.
F3SJD5_STRSA_Streptococcus_sanguinis
F2C766_STRSA_Streptococcus_sanguinis
A0A1L8X0X8_9ENT_Enterococcus_raffinosus
R5BJD8_9FIRM_Veillonella_sp.
A0A134BZS2_9FIR_Veillonella_dispar
H1GCT0_LISIN_Listeria_innocua
Q92CP0_LISIN_Listeria_innocua
Bacteria_Terrabacteriagroup_Firmicutes_Bacilli_Bacillales_Listeriaceae_Listeria_Listeria_monocytogenes_Q8Y7V2_LISMO_GlpF_protein
A0A097B4H5_LISI_Listeria_ivanovii
E3ZPG3_LISSE_Listeria_seeligeri
A0A093B0D5_9PSE_Amycolatopsis_lurida
A0A1H2FJS4_9PSE_Amycolatopsis_keratiniphila
R4SVU5_AMYOR_Amycolatopsis_orientalis
A0A075USH8_9PSE_Amycolatopsis_japonica
M2Z3Q7_9PSEU_Amycolatopsis_decaplanina
A0A193C1P9_AMYO_Amycolatopsis_orientalis
A0A076MT42_AMYM_Amycolatopsis_methanolica
GlpF_EXU67399.1_Streptomyces_sp.
D2BFK5_STRRD_Streptosporangium_roseum
D2B1E8_STRRD_Streptosporangium_roseum
A0A0Q7K1I8_9ACT_Angustibacter_sp.
Bacteria_Terrabacteriagroup_Actinobacteria_Actinomycetia_Corynebacteriales_Mycobacteriaceae_Mycolicibacterium_Mycolicibacterium_smegmatis_A0R728_MYCS2_Transport_integral_membrane_protein
A0A0M2K3U1_9MYC_Mycobacterium_obuense
A0A099CDD6_9MYC_Mycobacterium_rufum
A0A0J6VMI2_9MYC_Mycobacterium_chlorophenolicum
A0A1A1YGB7_9MYC_Mycobacterium_sp.
A0A1Q8JMQ5_9PSE_Pseudonocardia_sp.
A0A0P0SF10_9PSE_Pseudonocardia_sp.
Bacteria_Terrabacteriagroup_Actinobacteria_Actinomycetia_Streptomycetales_Streptomycetaceae_Streptomyces_Streptomyces_albidoflavusgroup_Streptomyces_coelicolor_Q9AK60_STRCO_Putative_transport_integral_membrane_protein
W0RPR2_9BACT_Gemmatimonadetes_bacterium
GlpF_WP_014023489.1
Bacteria_Spirochaetes_Spirochaetia_Spirochaetales_Borreliaceae_Borreliella_Borrelia_burgdorferi_O51256_BORBU_Glycerol_uptake_facilitator
Q84I24_BORHE_Borrelia_hermsii
Bacteria_Terrabacteriagroup_DeinococcusThermus_Deinococci_Deinococcales_Deinococcaceae_Deinococcus_Deinococcus_radiodurans_Q9RT37_DEIRA_Glycerol_uptake_facilitator_protein
Bacteria_Proteobacteria_delta_epsilon_subdivisions_Deltaproteobacteria_Desulfovibrionales_Desulfovibrionaceae_Desulfovibrio_Desulfovibrio_vulgaris_Q726H5_DESVH_Glycerol_uptake_facilitator_protein
Bacteria_Acidobacteria_Acidobacteriia_Acidobacteriales_Acidobacteriaceae_Candidatus_Koribacter_Koribacter_versatilis_Q1IMB3_KORVE_MIP_family_channel_protein
A0A0D5NVR1_XANC_Xanthomonas_citri
A0A1T1N563_9XAN__Pseudomonas__cissicola
H1XGW0_9XANT_Xanthomonas_axonopodis
A0A1T1RAZ2_XANC_Xanthomonas_citri
A0A1S1XG93_9XAN_Xanthomonas_alfalfae
Q3BYQ9_XANC5_Xanthomonas_campestris
A0A061YYD6_9XAN_Xanthomonas_arboricola
A0A0K1M7L0_XANC_Xanthomonas_campestris
F0E3L6_9XANT_Xanthomonas_fragariae
F0C324_9XANT_Xanthomonas_gardneri
A0A1L5QUQ7_9XAN_Xanthomonas_vesicatoria
A0A0Q5IZV3_9XAN_Xanthomonas_sp.
A0A0B2CJP4_XANC_Xanthomonas_campestris
Bacteria_Proteobacteria_Gammaproteobacteria_Xanthomonadales_Xanthomonadaceae_Xanthomonas_Xanthomonas_campestris_Q8PDH9_XANCP_Glycerol_uptake_facilitator_protein
A0A1L2ATF2_XYLF_Xylella_fastidiosa
Q87BZ3_XYLFT_Xylella_fastidiosa
A0A0F3K4I0_9GAM_Luteibacter_yeojuensis
A0A1T5DM02_9GAM_Luteibacter_sp.
L7GJ50_XANCT_Xanthomonas_translucens
A0A0K2ZXP0_XANC_Xanthomonas_translucens
A0A199P219_XANC_Xanthomonas_translucens
A0A1M4JCQ3_XANC_Xanthomonas_translucens
D2UB47_XANAP_Xanthomonas_albilineans
A0A0A8E0C9_9XAN_Xanthomonas_sacchari
A0A0D0JJP6_STEM_Stenotrophomonas_maltophilia
A0A1L7AIH0_9PRO_Roseomonas_gilardii
A0A1Q2YTG5_9PRO_Roseomonas_sp.
A0A0Q4L7P6_9SPH_Sphingomonas_sp.
A0A0Q4JS51_9SPH_Sphingomonas_sp.
A0A1A0DEP2_ACEP_Acetobacter_pasteurianus
A0A0K0TCJ6_ACEP_Acetobacter_pasteurianus
A0A160BKT3_9PRO_Acetobacter_sp.
A0A063X2Z7_ACEA_Acetobacter_aceti
A0A0D6NUI1_9PRO_Acetobacter_orleanensis
A0A0C9LRV8_9PRO_Acetobacter_tropicalis
A0A0D6Q2H3_KOME_Komagataeibacter_europaeus
A0A0K8MZW3_9PRO_Gluconobacter_frateurii
A0A0S6THH2_9PRO_Gluconobacter_frateurii
A0A177G2P8_GLUJ_Gluconobacter_japonicus
A0A0D6PRF2_9PRO_Gluconobacter_frateurii
A0A149QSY0_GLUO_Gluconobacter_oxydans
A0A1D8URT9_9PRO_Kozakia_baliensis
A0A023D7I2_ACIM_Acidomonas_methanolica
A0A1R1YVF1_9SPH_Sphingomonas_sp.

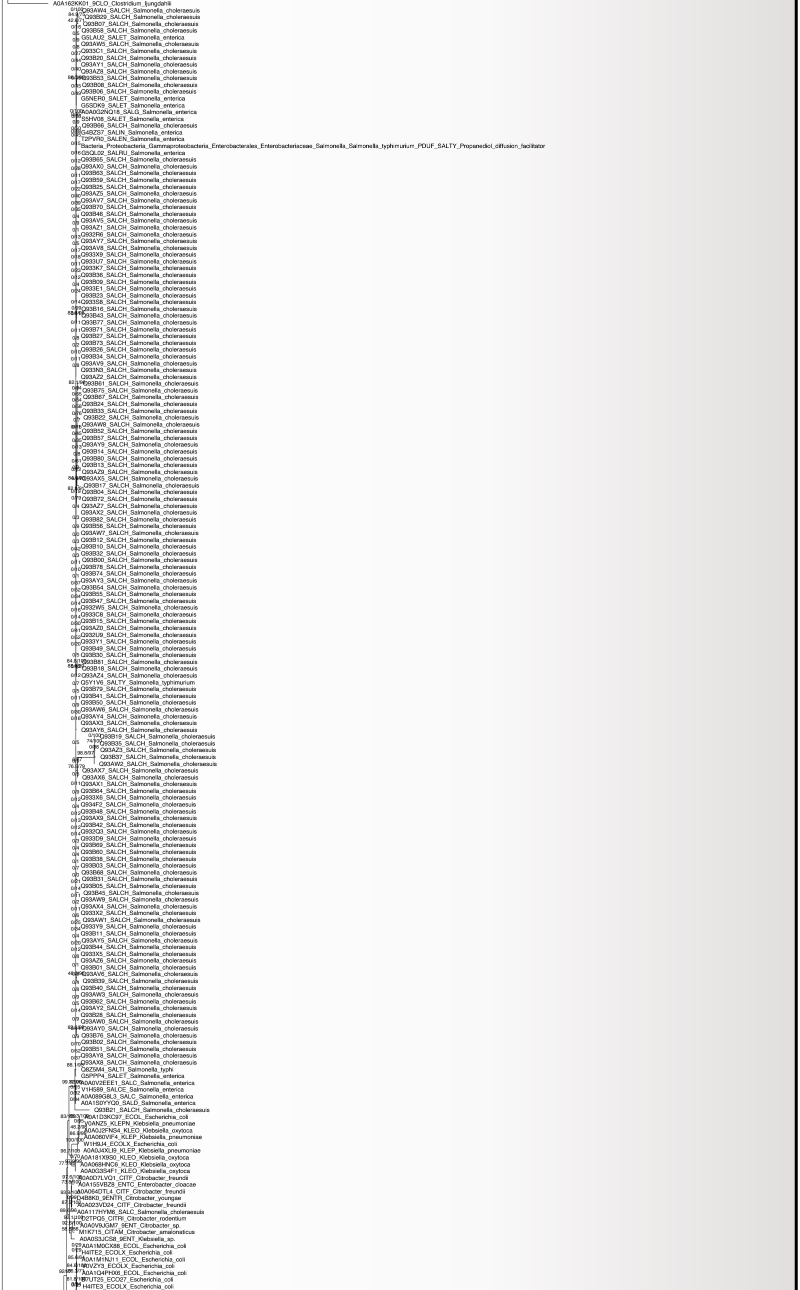
A0A162KK01_9CLO_Clostridium_ljungdahlii
Q93AW4_SALCH_Salmonella_choleraesuis
Q93B29_SALCH_Salmonella_choleraesuis
Q93B07_SALCH_Salmonella_choleraesuis
Q93B58_SALCH_Salmonella_choleraesuis
G5LAU2_SALET_Salmonella_enterica
Q93AW5_SALCH_Salmonella_choleraesuis
Q933C1_SALCH_Salmonella_choleraesuis
Q93B20_SALCH_Salmonella_choleraesuis
Q93AY1_SALCH_Salmonella_choleraesuis
Q93AZ8_SALCH_Salmonella_choleraesuis
Q93B53_SALCH_Salmonella_choleraesuis
Q93B08_SALCH_Salmonella_choleraesuis
Q93B06_SALCH_Salmonella_choleraesuis
G5NER0_SALET_Salmonella_enterica
G5SDK9_SALET_Salmonella_enterica
A0A0G2NQ18_SALG_Salmonella_enterica
S5HV08_SALET_Salmonella_enterica
Q93B66_SALCH_Salmonella_choleraesuis
G4BZS7_SALIN_Salmonella_enterica
T2PVR0_SALEN_Salmonella_enterica
Bacteria_Proteobacteria_Gammaproteobacteria_Enterobacterales_Enterobacteriaceae_Salmonella_Salmonella_typhimurium_PDUF_SALTY_Propanediol_diffusion_facilitator
G5QL02_SALRU_Salmonella_enterica
Q93B65_SALCH_Salmonella_choleraesuis
Q93AX0_SALCH_Salmonella_choleraesuis
Q93B63_SALCH_Salmonella_choleraesuis
Q93B59_SALCH_Salmonella_choleraesuis
Q93B25_SALCH_Salmonella_choleraesuis
Q93AZ5_SALCH_Salmonella_choleraesuis
Q93AV7_SALCH_Salmonella_choleraesuis
Q93B70_SALCH_Salmonella_choleraesuis
Q93B46_SALCH_Salmonella_choleraesuis
Q93AV5_SALCH_Salmonella_choleraesuis
Q93AZ1_SALCH_Salmonella_choleraesuis
Q932R6_SALCH_Salmonella_choleraesuis
Q93AY7_SALCH_Salmonella_choleraesuis
Q93AV8_SALCH_Salmonella_choleraesuis
Q933X9_SALCH_Salmonella_choleraesuis
Q933U7_SALCH_Salmonella_choleraesuis
Q933K7_SALCH_Salmonella_choleraesuis
Q93B36_SALCH_Salmonella_choleraesuis
Q93B09_SALCH_Salmonella_choleraesuis
Q933E1_SALCH_Salmonella_choleraesuis
Q93B23_SALCH_Salmonella_choleraesuis
Q933S8_SALCH_Salmonella_choleraesuis
Q93B16_SALCH_Salmonella_choleraesuis
Q93B43_SALCH_Salmonella_choleraesuis
Q93B77_SALCH_Salmonella_choleraesuis
Q93B71_SALCH_Salmonella_choleraesuis
Q93B27_SALCH_Salmonella_choleraesuis
Q93B73_SALCH_Salmonella_choleraesuis
Q93B26_SALCH_Salmonella_choleraesuis
Q93B34_SALCH_Salmonella_choleraesuis
Q93AV9_SALCH_Salmonella_choleraesuis
Q933N3_SALCH_Salmonella_choleraesuis
Q93AZ2_SALCH_Salmonella_choleraesuis
Q93B61_SALCH_Salmonella_choleraesuis
Q93B75_SALCH_Salmonella_choleraesuis
Q93B67_SALCH_Salmonella_choleraesuis
Q93B24_SALCH_Salmonella_choleraesuis
Q93B33_SALCH_Salmonella_choleraesuis
Q93B22_SALCH_Salmonella_choleraesuis
Q93AW8_SALCH_Salmonella_choleraesuis
Q93B52_SALCH_Salmonella_choleraesuis
Q93B57_SALCH_Salmonella_choleraesuis
Q93AY9_SALCH_Salmonella_choleraesuis
Q93B14_SALCH_Salmonella_choleraesuis
Q93B80_SALCH_Salmonella_choleraesuis
Q93B13_SALCH_Salmonella_choleraesuis
Q93AZ9_SALCH_Salmonella_choleraesuis
Q93AX5_SALCH_Salmonella_choleraesuis
Q93B17_SALCH_Salmonella_choleraesuis
Q93B04_SALCH_Salmonella_choleraesuis
Q93B72_SALCH_Salmonella_choleraesuis
Q93AZ7_SALCH_Salmonella_choleraesuis
Q93AX2_SALCH_Salmonella_choleraesuis
Q93B82_SALCH_Salmonella_choleraesuis
Q93B56_SALCH_Salmonella_choleraesuis
Q93AW7_SALCH_Salmonella_choleraesuis
Q93B12_SALCH_Salmonella_choleraesuis
Q93B10_SALCH_Salmonella_choleraesuis
Q93B32_SALCH_Salmonella_choleraesuis
Q93B00_SALCH_Salmonella_choleraesuis
Q93B78_SALCH_Salmonella_choleraesuis
Q93B74_SALCH_Salmonella_choleraesuis
Q93AY3_SALCH_Salmonella_choleraesuis
Q93B54_SALCH_Salmonella_choleraesuis
Q93B55_SALCH_Salmonella_choleraesuis
Q93B47_SALCH_Salmonella_choleraesuis
Q932W5_SALCH_Salmonella_choleraesuis
Q933C8_SALCH_Salmonella_choleraesuis
Q93B15_SALCH_Salmonella_choleraesuis
Q93AZ0_SALCH_Salmonella_choleraesuis
Q932U9_SALCH_Salmonella_choleraesuis
Q933Y1_SALCH_Salmonella_choleraesuis
Q93B49_SALCH_Salmonella_choleraesuis
Q93B30_SALCH_Salmonella_choleraesuis
Q93B81_SALCH_Salmonella_choleraesuis
Q93B18_SALCH_Salmonella_choleraesuis
Q93AZ4_SALCH_Salmonella_choleraesuis
Q5Y1V6_SALTY_Salmonella_typhimurium
Q93B79_SALCH_Salmonella_choleraesuis
Q93B41_SALCH_Salmonella_choleraesuis
Q93B50_SALCH_Salmonella_choleraesuis
Q93AW6_SALCH_Salmonella_choleraesuis
Q93AY4_SALCH_Salmonella_choleraesuis
Q93AX3_SALCH_Salmonella_choleraesuis
Q93AY6_SALCH_Salmonella_choleraesuis
Q93B19_SALCH_Salmonella_choleraesuis
Q93B35_SALCH_Salmonella_choleraesuis
Q93AZ3_SALCH_Salmonella_choleraesuis
Q93B37_SALCH_Salmonella_choleraesuis
Q93AW2_SALCH_Salmonella_choleraesuis
Q93AX7_SALCH_Salmonella_choleraesuis
Q93AX6_SALCH_Salmonella_choleraesuis
Q93AX1_SALCH_Salmonella_choleraesuis
Q93B64_SALCH_Salmonella_choleraesuis
Q933X6_SALCH_Salmonella_choleraesuis
Q934F2_SALCH_Salmonella_choleraesuis
Q93B48_SALCH_Salmonella_choleraesuis
Q93AX9_SALCH_Salmonella_choleraesuis
Q93B42_SALCH_Salmonella_choleraesuis
Q932Q3_SALCH_Salmonella_choleraesuis
Q933D9_SALCH_Salmonella_choleraesuis
Q93B69_SALCH_Salmonella_choleraesuis
Q93B60_SALCH_Salmonella_choleraesuis
Q93B38_SALCH_Salmonella_choleraesuis
Q93B03_SALCH_Salmonella_choleraesuis
Q93B68_SALCH_Salmonella_choleraesuis
Q93B31_SALCH_Salmonella_choleraesuis
Q93B05_SALCH_Salmonella_choleraesuis
Q93B45_SALCH_Salmonella_choleraesuis
Q93AW9_SALCH_Salmonella_choleraesuis
Q93AX4_SALCH_Salmonella_choleraesuis
Q933X2_SALCH_Salmonella_choleraesuis
Q93AW1_SALCH_Salmonella_choleraesuis
Q933Y9_SALCH_Salmonella_choleraesuis
Q93B11_SALCH_Salmonella_choleraesuis
Q93AY5_SALCH_Salmonella_choleraesuis
Q93B44_SALCH_Salmonella_choleraesuis
Q933X5_SALCH_Salmonella_choleraesuis
Q93AZ6_SALCH_Salmonella_choleraesuis
Q93B01_SALCH_Salmonella_choleraesuis
Q93AV6_SALCH_Salmonella_choleraesuis
Q93B39_SALCH_Salmonella_choleraesuis
Q93B40_SALCH_Salmonella_choleraesuis
Q93AW3_SALCH_Salmonella_choleraesuis
Q93B62_SALCH_Salmonella_choleraesuis
Q93AY2_SALCH_Salmonella_choleraesuis
Q93B28_SALCH_Salmonella_choleraesuis
Q93AW0_SALCH_Salmonella_choleraesuis
Q93AY0_SALCH_Salmonella_choleraesuis
Q93B76_SALCH_Salmonella_choleraesuis
Q93B02_SALCH_Salmonella_choleraesuis
Q93B51_SALCH_Salmonella_choleraesuis
Q93AY8_SALCH_Salmonella_choleraesuis
Q93AX8_SALCH_Salmonella_choleraesuis
Q8Z5M4_SALTI_Salmonella_typhi
G5PPP4_SALET_Salmonella_enterica
A0A0V2EEE1_SALC_Salmonella_enterica
V1H589_SALCE_Salmonella_enterica
A0A089G8L3_SALC_Salmonella_enterica
A0A1S0YYQ0_SALD_Salmonella_enterica
Q93B21_SALCH_Salmonella_choleraesuis
A0A1D3KC97_ECOL_Escherichia_coli
V0ANZ5_KLEPN_Klebsiella_pneumoniae
A0A0J2FNS4_KLEO_Klebsiella_oxytoca
A0A060VIF4_KLEP_Klebsiella_pneumoniae
W1H9J4_ECOLX_Escherichia_coli
A0A0J4XLI9_KLEP_Klebsiella_pneumoniae
A0A181X9S0_KLEO_Klebsiella_oxytoca
A0A068HNC6_KLEO_Klebsiella_oxytoca
A0A0G3S4F1_KLEO_Klebsiella_oxytoca
A0A0D7LVQ1_CITF_Citrobacter_freundii
A0A155VBZ8_ENTC_Enterobacter_cloacae
A0A064DTL4_CITF_Citrobacter_freundii
D4B8K0_9ENTR_Citrobacter_youngae
A0A023VD24_CITF_Citrobacter_freundii
A0A117HYM6_SALC_Salmonella_choleraesuis
D2TPQ5_CITRI_Citrobacter_rodentium
A0A0V9JGM7_9ENT_Citrobacter_sp.
M1K715_CITAM_Citrobacter_amalonaticus
A0A0S3JCS8_9ENT_Klebsiella_sp.
A0A1M0CX88_ECOL_Escherichia_coli
H4ITE2_ECOLX_Escherichia_coli
A0A1M1NJ11_ECOL_Escherichia_coli
V0VZY3_ECOLX_Escherichia_coli
A0A1Q4PHX6_ECOL_Escherichia_coli
B7UT25_ECO27_Escherichia_coli
H4ITE3_ECOLX_Escherichia_coli

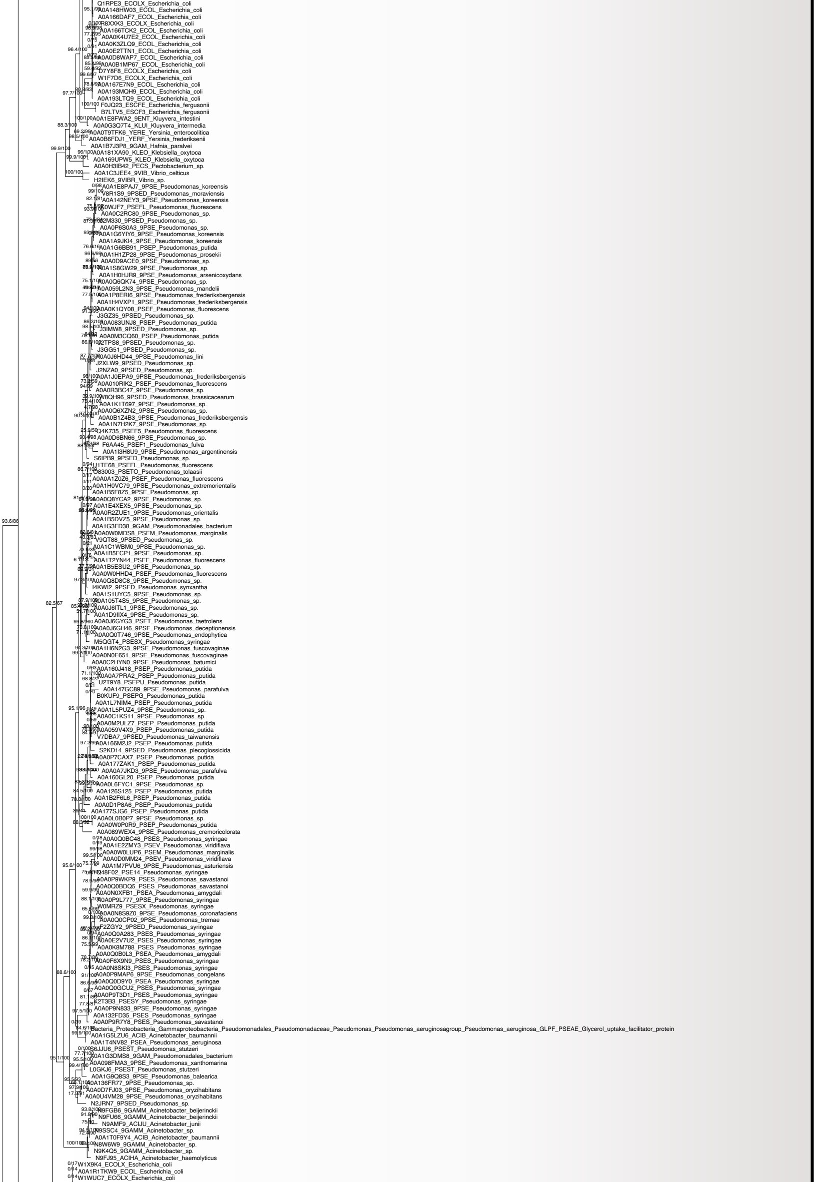
Q1RPE3_ECOLX_Escherichia_coli
A0A148HW03_ECOL_Escherichia_coli
A0A166DAF7_ECOL_Escherichia_coli
R8XXK3_ECOLX_Escherichia_coli
A0A166TCK2_ECOL_Escherichia_coli
A0A0K4U7E2_ECOL_Escherichia_coli
A0A0K3ZLQ9_ECOL_Escherichia_coli
A0A0E2TTN1_ECOL_Escherichia_coli
A0A0D8WAP7_ECOL_Escherichia_coli
A0A0B1MP67_ECOL_Escherichia_coli
D7Y8F8_ECOLX_Escherichia_coli
W1F7D6_ECOLX_Escherichia_coli
A0A167E7N9_ECOL_Escherichia_coli
A0A193MQH9_ECOL_Escherichia_coli
A0A193LTQ9_ECOL_Escherichia_coli
F0JQ23_ESCFE_Escherichia_fergusonii
B7LTV5_ESCF3_Escherichia_fergusonii
A0A1E8FWA2_9ENT_Kluyvera_intestini
A0A0G3Q7T4_KLUI_Kluyvera_intermedia
A0A0T9TFK6_YERE_Yersinia_enterocolitica
A0A0B6FDJ1_YERF_Yersinia_frederiksenii
A0A1B7J3P8_9GAM_Hafnia_paralvei
A0A181XA90_KLEO_Klebsiella_oxytoca
A0A169UPW5_KLEO_Klebsiella_oxytoca
A0A0H3IB42_PECS_Pectobacterium_sp.
A0A1C3JEE4_9VIB_Vibrio_celticus
H2IEK6_9VIBR_Vibrio_sp.
A0A1E8PAJ7_9PSE_Pseudomonas_koreensis
V8R1S9_9PSED_Pseudomonas_moraviensis
A0A142NEY3_9PSE_Pseudomonas_koreensis
K0WJF7_PSEFL_Pseudomonas_fluorescens
A0A0C2RC80_9PSE_Pseudomonas_sp.
J2M330_9PSED_Pseudomonas_sp.
A0A0P6S0A3_9PSE_Pseudomonas_sp.
A0A1G6YIY6_9PSE_Pseudomonas_koreensis
A0A1A9JKI4_9PSE_Pseudomonas_koreensis
A0A1G6BB91_PSEP_Pseudomonas_putida
A0A1H1ZP28_9PSE_Pseudomonas_prosekii
A0A0D9ACE0_9PSE_Pseudomonas_sp.
A0A1S8GW29_9PSE_Pseudomonas_sp.
A0A1H0HJR9_9PSE_Pseudomonas_arsenicoxydans
A0A0Q6QK74_9PSE_Pseudomonas_sp.
A0A059L2N3_9PSE_Pseudomonas_mandelii
A0A1P8ERI6_9PSE_Pseudomonas_frederiksbergensis
A0A1H4VXP1_9PSE_Pseudomonas_frederiksbergensis
A0A0K1QY08_PSEF_Pseudomonas_fluorescens
J3GZ35_9PSED_Pseudomonas_sp.
A0A083UNJ8_PSEP_Pseudomonas_putida
J3IMW8_9PSED_Pseudomonas_sp.
A0A0M3CQ60_PSEP_Pseudomonas_putida
J2TPS8_9PSED_Pseudomonas_sp.
J3GG51_9PSED_Pseudomonas_sp.
A0A0J6HD44_9PSE_Pseudomonas_lini
J2XLW9_9PSED_Pseudomonas_sp.
J2NZA0_9PSED_Pseudomonas_sp.
A0A1J0EPA9_9PSE_Pseudomonas_frederiksbergensis
A0A010RIK2_PSEF_Pseudomonas_fluorescens
A0A0R3BC47_9PSE_Pseudomonas_sp.
W8QH96_9PSED_Pseudomonas_brassicacearum
A0A1K1T697_9PSE_Pseudomonas_sp.
A0A0Q6XZN2_9PSE_Pseudomonas_sp.
A0A0B1Z4B3_9PSE_Pseudomonas_frederiksbergensis
A0A1N7H2K7_9PSE_Pseudomonas_sp.
Q4K735_PSEF5_Pseudomonas_fluorescens
A0A0D6BN66_9PSE_Pseudomonas_sp.
F6AA45_PSEF1_Pseudomonas_fulva
A0A1I3H8U9_9PSE_Pseudomonas_argentinensis
S6IPB9_9PSED_Pseudomonas_sp.
U1TE68_PSEFL_Pseudomonas_fluorescens
O83003_PSETO_Pseudomonas_tolaasii
A0A0A1Z0Z6_PSEF_Pseudomonas_fluorescens
A0A1H0VC79_9PSE_Pseudomonas_extremorientalis
A0A1B5F8Z5_9PSE_Pseudomonas_sp.
A0A0Q8YCA2_9PSE_Pseudomonas_sp.
A0A1E4XEX5_9PSE_Pseudomonas_sp.
A0A0R2ZUE1_9PSE_Pseudomonas_orientalis
A0A1B5DVZ5_9PSE_Pseudomonas_sp.
A0A1G3FD38_9GAM_Pseudomonadales_bacterium
A0A0W0MDS8_PSEM_Pseudomonas_marginalis
V9QT88_9PSED_Pseudomonas_sp.
A0A1C1WBM0_9PSE_Pseudomonas_sp.
A0A1B5FCP1_9PSE_Pseudomonas_sp.
A0A1T2YN44_PSEF_Pseudomonas_fluorescens
A0A1B5ESU2_9PSE_Pseudomonas_sp.
A0A0W0HHD4_PSEF_Pseudomonas_fluorescens
A0A0Q8D8C8_9PSE_Pseudomonas_sp.
I4KWI2_9PSED_Pseudomonas_synxantha
A0A1S1UYC5_9PSE_Pseudomonas_sp.
A0A105T4S5_9PSE_Pseudomonas_sp.
A0A0J6ITL1_9PSE_Pseudomonas_sp.
A0A1D9IIX4_9PSE_Pseudomonas_sp.
A0A0J6GYG3_PSET_Pseudomonas_taetrolens
A0A0J6GH46_9PSE_Pseudomonas_deceptionensis
A0A0Q0T746_9PSE_Pseudomonas_endophytica
M5QGT4_PSESX_Pseudomonas_syringae
A0A1H6N2G3_9PSE_Pseudomonas_fuscovaginae
A0A0N0E651_9PSE_Pseudomonas_fuscovaginae
A0A0C2HYN0_9PSE_Pseudomonas_batumici
A0A160J418_PSEP_Pseudomonas_putida
A0A0A7PRA2_PSEP_Pseudomonas_putida
U2T9Y8_PSEPU_Pseudomonas_putida
A0A147GC89_9PSE_Pseudomonas_parafulva
B0KUF9_PSEPG_Pseudomonas_putida
A0A1L7NIM4_PSEP_Pseudomonas_putida
A0A1L5PUZ4_9PSE_Pseudomonas_sp.
A0A0C1KS11_9PSE_Pseudomonas_sp.
A0A0M2ULZ7_PSEP_Pseudomonas_putida
A0A059V4X9_PSEP_Pseudomonas_putida
V7DBA7_9PSED_Pseudomonas_taiwanensis
A0A166M2J2_PSEP_Pseudomonas_putida
S2KD14_9PSED_Pseudomonas_plecoglossicida
A0A0P7CAX7_PSEP_Pseudomonas_putida
A0A177ZAK1_PSEP_Pseudomonas_putida
A0A0A7JKD3_9PSE_Pseudomonas_parafulva
A0A160GL20_PSEP_Pseudomonas_putida
A0A0L6FYC1_9PSE_Pseudomonas_sp.
A0A126S125_PSEP_Pseudomonas_putida
A0A1B2F6L6_PSEP_Pseudomonas_putida
A0A0D1P8A6_PSEP_Pseudomonas_putida
A0A177SJG6_PSEP_Pseudomonas_putida
A0A0L0B0P7_9PSE_Pseudomonas_sp.
A0A0W0P0R9_PSEP_Pseudomonas_putida
A0A089WEX4_9PSE_Pseudomonas_cremoricolorata
A0A0Q0BC48_PSES_Pseudomonas_syringae
A0A1E2ZMY3_PSEV_Pseudomonas_viridiflava
A0A0W0LUP6_PSEM_Pseudomonas_marginalis
A0A0D0MM24_PSEV_Pseudomonas_viridiflava
A0A1M7PVU6_9PSE_Pseudomonas_asturiensis
Q48F02_PSE14_Pseudomonas_syringae
A0A0P9WKP9_PSES_Pseudomonas_savastanoi
A0A0Q0BDQ5_PSES_Pseudomonas_savastanoi
A0A0N0XFB1_PSEA_Pseudomonas_amygdali
A0A0P9L777_9PSE_Pseudomonas_syringae
W0MRZ9_PSESX_Pseudomonas_syringae
A0A0N8S9Z0_9PSE_Pseudomonas_coronafaciens
A0A0Q0CP02_9PSE_Pseudomonas_tremae
F3ZGY2_9PSED_Pseudomonas_syringae
A0A0Q0A283_PSES_Pseudomonas_syringae
A0A0E2V7U2_PSES_Pseudomonas_syringae
A0A0K8M788_PSES_Pseudomonas_syringae
A0A0Q0B0L3_PSEA_Pseudomonas_amygdali
A0A0F6X9N9_PSES_Pseudomonas_syringae
A0A0N8SKI3_PSES_Pseudomonas_syringae
A0A0P9MAP6_9PSE_Pseudomonas_congelans
A0A0Q0D9Y0_PSEA_Pseudomonas_syringae
A0A0Q0GCU2_PSES_Pseudomonas_syringae
A0A0P9T3D1_PSES_Pseudomonas_syringae
K2T3B3_PSESY_Pseudomonas_syringae
A0A0P9N833_9PSE_Pseudomonas_syringae
A0A132FD35_PSES_Pseudomonas_syringae
A0A0P9R7Y8_PSES_Pseudomonas_savastanoi
Bacteria_Proteobacteria_Gammaproteobacteria_Pseudomonadales_Pseudomonadaceae_Pseudomonas_Pseudomonas_aeruginosagroup_Pseudomonas_aeruginosa_GLPF_PSEAE_Glycerol_uptake_facilitator_protein
A0A1G5LZU6_ACIB_Acinetobacter_baumannii
A0A1T4NV82_PSEA_Pseudomonas_aeruginosa
S6JJU6_PSEST_Pseudomonas_stutzeri
A0A1G3DMS8_9GAM_Pseudomonadales_bacterium
A0A098FMA3_9PSE_Pseudomonas_xanthomarina
L0GKJ6_PSEST_Pseudomonas_stutzeri
A0A1G9Q8S3_9PSE_Pseudomonas_balearica
A0A136FR77_9PSE_Pseudomonas_sp.
A0A0D7FJ03_9PSE_Pseudomonas_oryzihabitans
A0A0U4VM28_9PSE_Pseudomonas_oryzihabitans
N2JRN7_9PSED_Pseudomonas_sp.
N9FGB6_9GAMM_Acinetobacter_beijerinckii
N9FU66_9GAMM_Acinetobacter_beijerinckii
N9AMF9_ACIJU_Acinetobacter_junii
N9SSC4_9GAMM_Acinetobacter_sp.
A0A1T0F9Y4_ACIB_Acinetobacter_baumannii
N8W6W9_9GAMM_Acinetobacter_sp.
N9K4Q5_9GAMM_Acinetobacter_sp.
N9FJ95_ACIHA_Acinetobacter_haemolyticus
W1X9K4_ECOLX_Escherichia_coli
A0A1R1TKW9_ECOL_Escherichia_coli
W1WUC7_ECOLX_Escherichia_coli

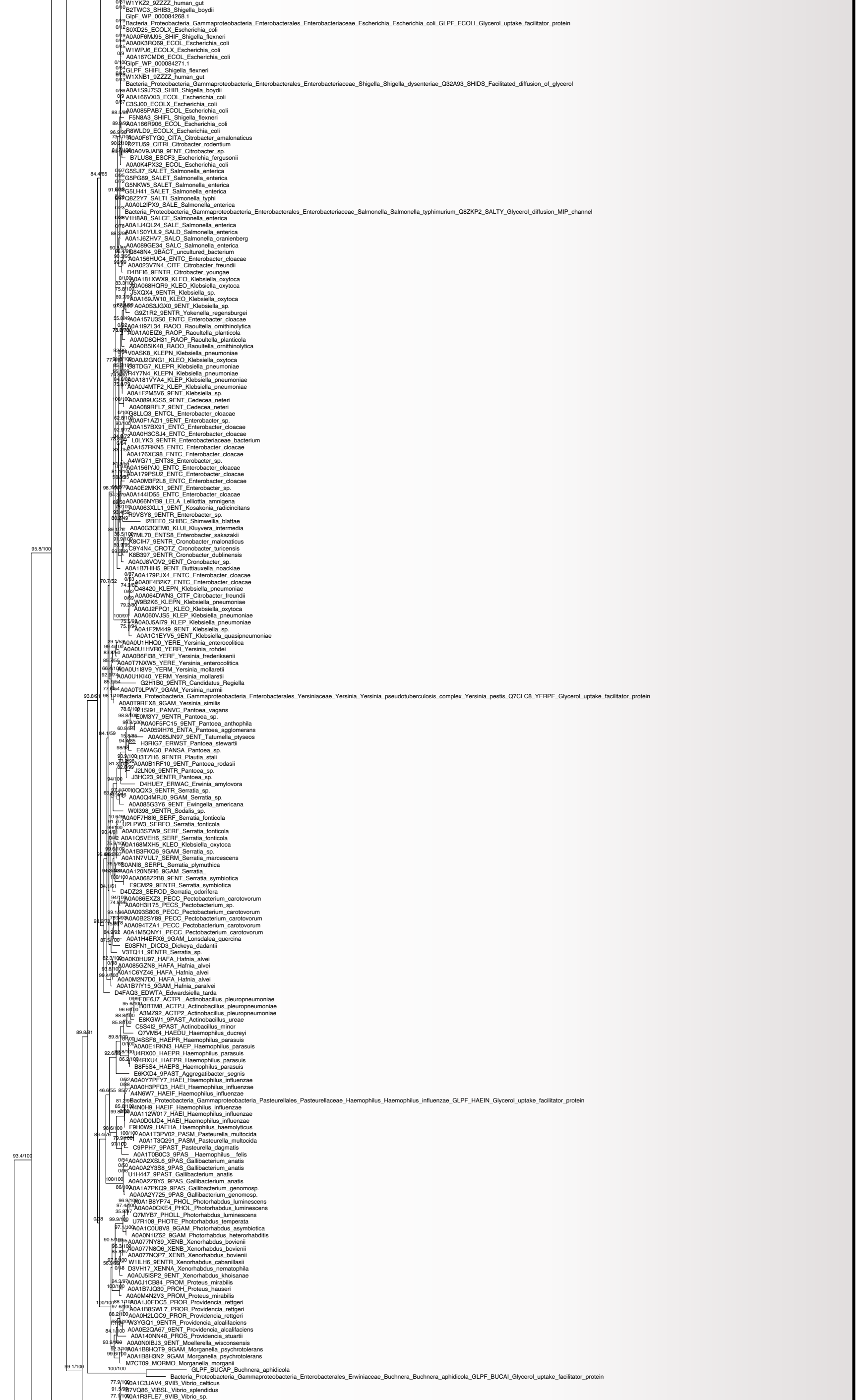

W1YKZ2_9ZZZZ_human_gut
B2TWC3_SHIB3_Shigella_boydii
GlpF_WP_000084268.1
Bacteria_Proteobacteria_Gammaproteobacteria_Enterobacterales_Enterobacteriaceae_Escherichia_Escherichia_coli_GLPF_ECOLI_Glycerol_uptake_facilitator_protein
S0XD25_ECOLX_Escherichia_coli
A0A0F6MJ95_SHIF_Shigella_flexneri
A0A0K3RQ69_ECOL_Escherichia_coli
W1WPJ6_ECOLX_Escherichia_coli
A0A167CMD6_ECOL_Escherichia_coli
GlpF_WP_000084271.1
GLPF_SHIFL_Shigella_flexneri
W1XNB1_9ZZZZ_human_gut
Bacteria_Proteobacteria_Gammaproteobacteria_Enterobacterales_Enterobacteriaceae_Shigella_Shigella_dysenteriae_Q32A93_SHIDS_Facilitated_diffusion_of_glycerol
A0A1S9J7S3_SHIB_Shigella_boydii
A0A166VXI3_ECOL_Escherichia_coli
C3SJ00_ECOLX_Escherichia_coli
A0A085PAB7_ECOL_Escherichia_coli
F5N8A3_SHIFL_Shigella_flexneri
A0A166R906_ECOL_Escherichia_coli
R8WLD9_ECOLX_Escherichia_coli
A0A0F6TYG0_CITA_Citrobacter_amalonaticus
D2TU59_CITRI_Citrobacter_rodentium
A0A0V9JAB9_9ENT_Citrobacter_sp.
B7LUS8_ESCF3_Escherichia_fergusonii
A0A0K4PX32_ECOL_Escherichia_coli
G5SJI7_SALET_Salmonella_enterica
G5PG89_SALET_Salmonella_enterica
G5NKW5_SALET_Salmonella_enterica
G5LH41_SALET_Salmonella_enterica
Q8Z2Y7_SALTI_Salmonella_typhi
A0A0L2IPX9_SALE_Salmonella_enterica
Bacteria_Proteobacteria_Gammaproteobacteria_Enterobacterales_Enterobacteriaceae_Salmonella_Salmonella_typhimurium_Q8ZKP2_SALTY_Glycerol_diffusion_MIP_channel
V1H8A8_SALCE_Salmonella_enterica
A0A1J4QL24_SALE_Salmonella_enterica
A0A1S0YUL9_SALD_Salmonella_enterica
A0A1J6ZHV7_SALO_Salmonella_oranienberg
A0A089GE34_SALC_Salmonella_enterica
Q848N4_9BACT_uncultured_bacterium
A0A156HUC4_ENTC_Enterobacter_cloacae
A0A023V7N4_CITF_Citrobacter_freundii
D4BEI6_9ENTR_Citrobacter_youngae
A0A181XWX9_KLEO_Klebsiella_oxytoca
A0A068HQR9_KLEO_Klebsiella_oxytoca
J5XQX4_9ENTR_Klebsiella_sp.
A0A169JW10_KLEO_Klebsiella_oxytoca
A0A0S3JGX0_9ENT_Klebsiella_sp.
G9Z1R2_9ENTR_Yokenella_regensburgei
A0A157U3S0_ENTC_Enterobacter_cloacae
A0A1I9ZL34_RAOO_Raoultella_ornithinolytica
A0A1A0EIZ6_RAOP_Raoultella_planticola
A0A0D8QH31_RAOP_Raoultella_planticola
A0A0B5IK48_RAOO_Raoultella_ornithinolytica
V0ASK8_KLEPN_Klebsiella_pneumoniae
A0A0J2GNG1_KLEO_Klebsiella_oxytoca
G8TDG7_KLEPR_Klebsiella_pneumoniae
R4Y7N4_KLEPN_Klebsiella_pneumoniae
A0A181VYA4_KLEP_Klebsiella_pneumoniae
A0A0J4MTF2_KLEP_Klebsiella_pneumoniae
A0A1F2M5V6_9ENT_Klebsiella_sp.
A0A089UGS5_9ENT_Cedecea_neteri
A0A089RFL7_9ENT_Cedecea_neteri
G8LLQ3_ENTCL_Enterobacter_cloacae
A0A0F1AZI1_9ENT_Enterobacter_sp.
A0A157BX91_ENTC_Enterobacter_cloacae
A0A0H3CSJ4_ENTC_Enterobacter_cloacae
L0LYK3_9ENTR_Enterobacteriaceae_bacterium
A0A157RKN5_ENTC_Enterobacter_cloacae
A0A176XC98_ENTC_Enterobacter_cloacae
A4WG71_ENT38_Enterobacter_sp.
A0A156IYJ0_ENTC_Enterobacter_cloacae
A0A179PSU2_ENTC_Enterobacter_cloacae
A0A0M3F2L8_ENTC_Enterobacter_cloacae
A0A0E2MKK1_9ENT_Enterobacter_sp.
A0A144ID55_ENTC_Enterobacter_cloacae
A0A066NYB9_LELA_Lelliottia_amnigena
A0A063XLL1_9ENT_Kosakonia_radicincitans
R9VSY8_9ENTR_Enterobacter_sp.
I2BEE0_SHIBC_Shimwellia_blattae
A0A0G3QEM0_KLUI_Kluyvera_intermedia
A7ML70_ENTS8_Enterobacter_sakazakii
K8CIH7_9ENTR_Cronobacter_malonaticus
C9Y4N4_CROTZ_Cronobacter_turicensis
K8B397_9ENTR_Cronobacter_dublinensis
A0A0J8VQV2_9ENT_Cronobacter_sp.
A0A1B7HIH5_9ENT_Buttiauxella_noackiae
A0A179PJX4_ENTC_Enterobacter_cloacae
A0A0F4B2K7_ENTC_Enterobacter_cloacae
Q48420_KLEPN_Klebsiella_pneumoniae
A0A064DWN3_CITF_Citrobacter_freundii
W9B2K6_KLEPN_Klebsiella_pneumoniae
A0A0J2FPQ1_KLEO_Klebsiella_oxytoca
A0A060VJS5_KLEP_Klebsiella_pneumoniae
A0A0J5AI79_KLEP_Klebsiella_pneumoniae
A0A1F2M449_9ENT_Klebsiella_sp.
A0A1C1EYV5_9ENT_Klebsiella_quasipneumoniae
A0A0U1HHQ0_YERE_Yersinia_enterocolitica
A0A0U1HVR0_YERR_Yersinia_rohdei
A0A0B6FI38_YERF_Yersinia_frederiksenii
A0A0T7NXW5_YERE_Yersinia_enterocolitica
A0A0U1I8V9_YERM_Yersinia_mollaretii
A0A0U1KI40_YERM_Yersinia_mollaretii
G2H1B0_9ENTR_Candidatus_Regiella
A0A0T9LPW7_9GAM_Yersinia_nurmii
Bacteria_Proteobacteria_Gammaproteobacteria_Enterobacterales_Yersiniaceae_Yersinia_Yersinia_pseudotuberculosis_complex_Yersinia_pestis_Q7CLC8_YERPE_Glycerol_uptake_facilitator_protein
A0A0T9REX8_9GAM_Yersinia_similis
E1SI91_PANVC_Pantoea_vagans
E0M3Y7_9ENTR_Pantoea_sp.
A0A0F5FC15_9ENT_Pantoea_anthophila
A0A059IH76_ENTA_Pantoea_agglomerans
A0A085JN97_9ENT_Tatumella_ptyseos
H3RIG7_ERWST_Pantoea_stewartii
E6WAG0_PANSA_Pantoea_sp.
U3TZH6_9ENTR_Plautia_stali
A0A0B1RF10_9ENT_Pantoea_rodasii
J2LN06_9ENTR_Pantoea_sp.
J3HC23_9ENTR_Pantoea_sp.
D4HUE7_ERWAC_Erwinia_amylovora
I0QQX3_9ENTR_Serratia_sp.
A0A0Q4MRJ0_9GAM_Serratia_sp.
A0A085G3Y6_9ENT_Ewingella_americana
W0I398_9ENTR_Sodalis_sp.
A0A0F7H8I6_SERF_Serratia_fonticola
U2LPW3_SERFO_Serratia_fonticola
A0A0U3S7W9_SERF_Serratia_fonticola
A0A1Q5VEH6_SERF_Serratia_fonticola
A0A168MXH5_KLEO_Klebsiella_oxytoca
A0A1B3FKQ6_9GAM_Serratia_sp.
A0A1N7VUL7_SERM_Serratia_marcescens
S0ANI8_SERPL_Serratia_plymuthica
A0A120N5R6_9GAM_Serratia_
A0A068Z2B8_9ENT_Serratia_symbiotica
E9CM29_9ENTR_Serratia_symbiotica
D4DZ23_SEROD_Serratia_odorifera
A0A086EXZ3_PECC_Pectobacterium_carotovorum
A0A0H3I175_PECS_Pectobacterium_sp.
A0A093S806_PECC_Pectobacterium_carotovorum
A0A0B2SY89_PECC_Pectobacterium_carotovorum
A0A094TZA1_PECC_Pectobacterium_carotovorum
A0A1M5QNY1_PECC_Pectobacterium_carotovorum
A0A1H4ERX6_9GAM_Lonsdalea_quercina
E0SFN1_DICD3_Dickeya_dadantii
V3TQ11_9ENTR_Serratia_sp.
A0A0K0HU97_HAFA_Hafnia_alvei
A0A085GZN8_HAFA_Hafnia_alvei
A0A1C6YZ46_HAFA_Hafnia_alvei
A0A0M2N7D0_HAFA_Hafnia_alvei
A0A1B7IY15_9GAM_Hafnia_paralvei
D4FAQ3_EDWTA_Edwardsiella_tarda
E0E6J7_ACTPL_Actinobacillus_pleuropneumoniae
D0BTM8_ACTPJ_Actinobacillus_pleuropneumoniae
A3MZ92_ACTP2_Actinobacillus_pleuropneumoniae
E8KGW1_9PAST_Actinobacillus_ureae
C5S4I2_9PAST_Actinobacillus_minor
Q7VM54_HAEDU_Haemophilus_ducreyi
U4SSF8_HAEPR_Haemophilus_parasuis
A0A0E1RKN3_HAEP_Haemophilus_parasuis
U4RX00_HAEPR_Haemophilus_parasuis
U4RXU4_HAEPR_Haemophilus_parasuis
B8F5S4_HAEPS_Haemophilus_parasuis
E6KXD4_9PAST_Aggregatibacter_segnis
A0A0Y7PFY7_HAEI_Haemophilus_influenzae
A0A0H3PFQ3_HAEI_Haemophilus_influenzae
A4N6W7_HAEIF_Haemophilus_influenzae
Bacteria_Proteobacteria_Gammaproteobacteria_Pasteurellales_Pasteurellaceae_Haemophilus_Haemophilus_influenzae_GLPF_HAEIN_Glycerol_uptake_facilitator_protein
A4N0H9_HAEIF_Haemophilus_influenzae
A0A112W017_HAEI_Haemophilus_influenzae
A0A0D0IJD4_HAEI_Haemophilus_influenzae
F9H0W9_HAEHA_Haemophilus_haemolyticus
A0A1T3PV02_PASM_Pasteurella_multocida
A0A1T3Q291_PASM_Pasteurella_multocida
C9PPH7_9PAST_Pasteurella_dagmatis
A0A1T0B0C3_9PAS__Haemophilus__felis
A0A0A2XSL6_9PAS_Gallibacterium_anatis
A0A0A2Y3S8_9PAS_Gallibacterium_anatis
U1H447_9PAST_Gallibacterium_anatis
A0A0A2Z8Y5_9PAS_Gallibacterium_anatis
A0A1A7PKQ9_9PAS_Gallibacterium_genomosp.
A0A0A2Y725_9PAS_Gallibacterium_genomosp.
A0A1B8YP74_PHOL_Photorhabdus_luminescens
A0A0A0CKE4_PHOL_Photorhabdus_luminescens
Q7MYB7_PHOLL_Photorhabdus_luminescens
U7R108_PHOTE_Photorhabdus_temperata
A0A1C0U8V8_9GAM_Photorhabdus_asymbiotica
A0A0N1IZ52_9GAM_Photorhabdus_heterorhabditis
A0A077NY89_XENB_Xenorhabdus_bovienii
A0A077N8Q6_XENB_Xenorhabdus_bovienii
A0A077NQP7_XENB_Xenorhabdus_bovienii
W1ILH6_9ENTR_Xenorhabdus_cabanillasii
D3VH17_XENNA_Xenorhabdus_nematophila
A0A0J5ISP2_9ENT_Xenorhabdus_khoisanae
A0A0J1CB84_PROM_Proteus_mirabilis
A0A1B7JQ30_PROH_Proteus_hauseri
A0A0M4N2V3_PROM_Proteus_mirabilis
A0A1J0EDC5_PROR_Providencia_rettgeri
A0A1B8SWL7_PROR_Providencia_rettgeri
A0A0H2LQC9_PROR_Providencia_rettgeri
W3YGQ1_9ENTR_Providencia_alcalifaciens
A0A0E2QA67_9ENT_Providencia_alcalifaciens
A0A140NN48_PROS_Providencia_stuartii
A0A0N0IBJ3_9ENT_Moellerella_wisconsensis
A0A1B8HQT9_9GAM_Morganella_psychrotolerans
A0A1B8H3N2_9GAM_Morganella_psychrotolerans
M7CT09_MORMO_Morganella_morganii
GLPF_BUCAP_Buchnera_aphidicola
Bacteria_Proteobacteria_Gammaproteobacteria_Enterobacterales_Erwiniaceae_Buchnera_Buchnera_aphidicola_GLPF_BUCAI_Glycerol_uptake_facilitator_protein
A0A1C3JAV4_9VIB_Vibrio_celticus
B7VQ86_VIBSL_Vibrio_splendidus
A0A1R3FLE7_9VIB_Vibrio_sp.

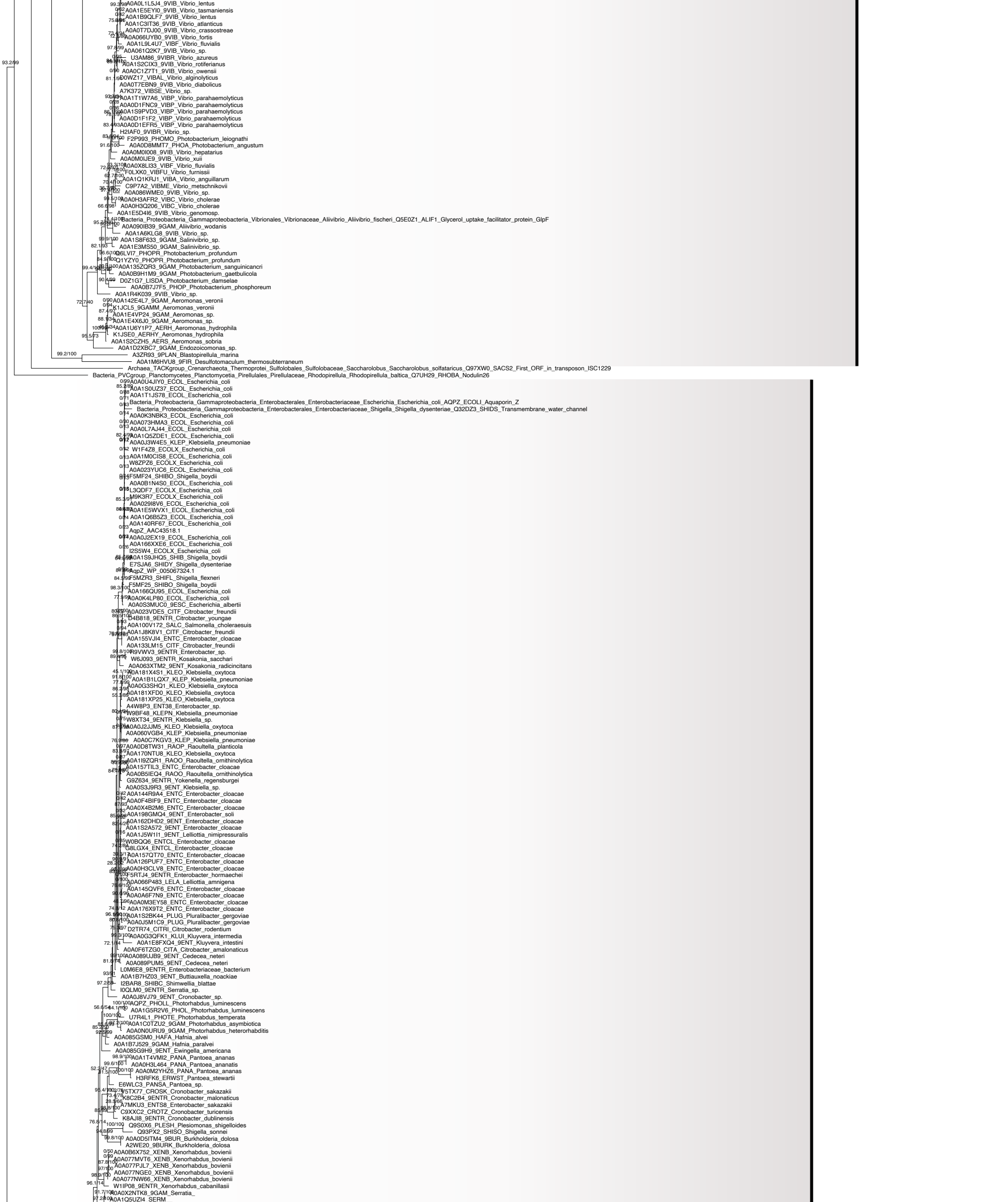
A0A0L1L5J4_9VIB_Vibrio_lentus
A0A1E5EYI0_9VIB_Vibrio_tasmaniensis
A0A1B9QLF7_9VIB_Vibrio_lentus
A0A1C3IT36_9VIB_Vibrio_atlanticus
A0A0T7DJ00_9VIB_Vibrio_crassostreae
A0A066UYB0_9VIB_Vibrio_fortis
A0A1L9L4U7_VIBF_Vibrio_fluvialis
A0A061Q2K7_9VIB_Vibrio_sp.
U3AM86_9VIBR_Vibrio_azureus
A0A1S2CIX3_9VIB_Vibrio_rotiferianus
A0A0C1Z7T1_9VIB_Vibrio_owensii
D0WZ17_VIBAL_Vibrio_alginolyticus
A0A0T7EBN9_9VIB_Vibrio_diabolicus
A7K372_VIBSE_Vibrio_sp.
A0A1T1W7A6_VIBP_Vibrio_parahaemolyticus
A0A0D1FNC9_VIBP_Vibrio_parahaemolyticus
A0A1S9PVD3_VIBP_Vibrio_parahaemolyticus
A0A0D1F1F2_VIBP_Vibrio_parahaemolyticus
A0A0D1EFR5_VIBP_Vibrio_parahaemolyticus
H2IAF0_9VIBR_Vibrio_sp.
F2P993_PHOMO_Photobacterium_leiognathi
A0A0D8MMT7_PHOA_Photobacterium_angustum
A0A0M0I008_9VIB_Vibrio_hepatarius
A0A0M0IJE9_9VIB_Vibrio_xuii
A0A0X8LI33_VIBF_Vibrio_fluvialis
F0LXK0_VIBFU_Vibrio_furnissii
A0A1Q1KRJ1_VIBA_Vibrio_anguillarum
C9P7A2_VIBME_Vibrio_metschnikovii
A0A086WME0_9VIB_Vibrio_sp.
A0A0H3AFR2_VIBC_Vibrio_cholerae
A0A0H3Q206_VIBC_Vibrio_cholerae
A0A1E5D4I6_9VIB_Vibrio_genomosp.
Bacteria_Proteobacteria_Gammaproteobacteria_Vibrionales_Vibrionaceae_Aliivibrio_Aliivibrio_fischeri_Q5E0Z1_ALIF1_Glycerol_uptake_facilitator_protein_GlpF
A0A090IB39_9GAM_Aliivibrio_wodanis
A0A1A6KLG8_9VIB_Vibrio_sp.
A0A1S8F633_9GAM_Salinivibrio_sp.
A0A1E3MS50_9GAM_Salinivibrio_sp.
Q6LVI7_PHOPR_Photobacterium_profundum
Q1YZY0_PHOPR_Photobacterium_profundum
A0A135ZQR3_9GAM_Photobacterium_sanguinicancri
A0A0B9H1M9_9GAM_Photobacterium_gaetbulicola
D0Z1G7_LISDA_Photobacterium_damselae
A0A0B7J7F5_PHOP_Photobacterium_phosphoreum
A0A1R4K039_9VIB_Vibrio_sp.
A0A142E4L7_9GAM_Aeromonas_veronii
K1JCL5_9GAMM_Aeromonas_veronii
A0A1E4VP24_9GAM_Aeromonas_sp.
A0A1E4X6J0_9GAM_Aeromonas_sp.
A0A1U6Y1P7_AERH_Aeromonas_hydrophila
K1JSE0_AERHY_Aeromonas_hydrophila
A0A1S2CZH5_AERS_Aeromonas_sobria
A0A1D2XBC7_9GAM_Endozoicomonas_sp.
A3ZR93_9PLAN_Blastopirellula_marina
A0A1M6HVU8_9FIR_Desulfotomaculum_thermosubterraneum
Archaea_TACKgroup_Crenarchaeota_Thermoprotei_Sulfolobales_Sulfolobaceae_Saccharolobus_Saccharolobus_solfataricus_Q97XW0_SACS2_First_ORF_in_transposon_ISC1229
Bacteria_PVCgroup_Planctomycetes_Planctomycetia_Pirellulales_Pirellulaceae_Rhodopirellula_Rhodopirellula_baltica_Q7UH29_RHOBA_Nodulin26
A0A0U4JIY0_ECOL_Escherichia_coli
A0A1S0UZ37_ECOL_Escherichia_coli
A0A1T1JS78_ECOL_Escherichia_coli
Bacteria_Proteobacteria_Gammaproteobacteria_Enterobacterales_Enterobacteriaceae_Escherichia_Escherichia_coli_AQPZ_ECOLI_Aquaporin_Z
Bacteria_Proteobacteria_Gammaproteobacteria_Enterobacterales_Enterobacteriaceae_Shigella_Shigella_dysenteriae_Q32DZ3_SHIDS_Transmembrane_water_channel
A0A0K3NBK3_ECOL_Escherichia_coli
A0A073HMA3_ECOL_Escherichia_coli
A0A0L7AJ44_ECOL_Escherichia_coli
A0A1Q5ZDE1_ECOL_Escherichia_coli
A0A0J3W4E5_KLEP_Klebsiella_pneumoniae
W1F4Z8_ECOLX_Escherichia_coli
A0A1M0CIS8_ECOL_Escherichia_coli
W8ZPZ6_ECOLX_Escherichia_coli
A0A023YUC6_ECOL_Escherichia_coli
F5MF24_SHIBO_Shigella_boydii
A0A0B1N4S0_ECOL_Escherichia_coli
L3QDF7_ECOLX_Escherichia_coli
M9K3R7_ECOLX_Escherichia_coli
A0A029I8V6_ECOL_Escherichia_coli
A0A1E5WVX1_ECOL_Escherichia_coli
A0A1Q6B5Z3_ECOL_Escherichia_coli
A0A140RF67_ECOL_Escherichia_coli
AqpZ_AAC43518.1
A0A0J2EX19_ECOL_Escherichia_coli
A0A166XXE6_ECOL_Escherichia_coli
I2S5W4_ECOLX_Escherichia_coli
A0A1S9JHQ5_SHIB_Shigella_boydii
E7SJA6_SHIDY_Shigella_dysenteriae
AqpZ_WP_005067324.1
F5MZR3_SHIFL_Shigella_flexneri
F5MF25_SHIBO_Shigella_boydii
A0A166QU95_ECOL_Escherichia_coli
A0A0K4LP80_ECOL_Escherichia_coli
A0A0S3MUC0_9ESC_Escherichia_albertii
A0A023VDE5_CITF_Citrobacter_freundii
D4B818_9ENTR_Citrobacter_youngae
A0A100V172_SALC_Salmonella_choleraesuis
A0A1J8K8V1_CITF_Citrobacter_freundii
A0A155VJI4_ENTC_Enterobacter_cloacae
A0A133LM15_CITF_Citrobacter_freundii
R9VWV3_9ENTR_Enterobacter_sp.
W6J093_9ENTR_Kosakonia_sacchari
A0A063XTM2_9ENT_Kosakonia_radicincitans
A0A181X4S1_KLEO_Klebsiella_oxytoca
A0A1B1LQX7_KLEP_Klebsiella_pneumoniae
A0A0G3SHQ1_KLEO_Klebsiella_oxytoca
A0A181XFD0_KLEO_Klebsiella_oxytoca
A0A181XP25_KLEO_Klebsiella_oxytoca
A4W8P3_ENT38_Enterobacter_sp.
W9BF48_KLEPN_Klebsiella_pneumoniae
W8XT34_9ENTR_Klebsiella_sp.
A0A0J2JJM5_KLEO_Klebsiella_oxytoca
A0A060VGB4_KLEP_Klebsiella_pneumoniae
A0A0C7KGV3_KLEP_Klebsiella_pneumoniae
A0A0D8TW31_RAOP_Raoultella_planticola
A0A170NTU8_KLEO_Klebsiella_oxytoca
A0A1I9ZQR1_RAOO_Raoultella_ornithinolytica
A0A157TIL3_ENTC_Enterobacter_cloacae
A0A0B5IEQ4_RAOO_Raoultella_ornithinolytica
G9Z634_9ENTR_Yokenella_regensburgei
A0A0S3J9R3_9ENT_Klebsiella_sp.
A0A144R9A4_ENTC_Enterobacter_cloacae
A0A0F4BIF9_ENTC_Enterobacter_cloacae
A0A0X4B2M6_ENTC_Enterobacter_cloacae
A0A198GMQ4_9ENT_Enterobacter_soli
A0A162DHD2_9ENT_Enterobacter_cloacae
A0A1S2A572_9ENT_Enterobacter_cloacae
A0A1J5W1I1_9ENT_Lelliottia_nimipressuralis
W0BQQ6_ENTCL_Enterobacter_cloacae
G8LGX4_ENTCL_Enterobacter_cloacae
A0A157QT70_ENTC_Enterobacter_cloacae
A0A126PUF7_ENTC_Enterobacter_cloacae
A0A0H3CLV8_ENTC_Enterobacter_cloacae
F5RTJ4_9ENTR_Enterobacter_hormaechei
A0A066P483_LELA_Lelliottia_amnigena
A0A145QVF6_ENTC_Enterobacter_cloacae
A0A0A6F7N9_ENTC_Enterobacter_cloacae
A0A0M3EY58_ENTC_Enterobacter_cloacae
A0A176X9T2_ENTC_Enterobacter_cloacae
A0A1S2BK44_PLUG_Pluralibacter_gergoviae
A0A0J5M1C9_PLUG_Pluralibacter_gergoviae
D2TR74_CITRI_Citrobacter_rodentium
A0A0G3QFK1_KLUI_Kluyvera_intermedia
A0A1E8FXQ4_9ENT_Kluyvera_intestini
A0A0F6TZG0_CITA_Citrobacter_amalonaticus
A0A089UJB9_9ENT_Cedecea_neteri
A0A089PUM5_9ENT_Cedecea_neteri
L0M6E8_9ENTR_Enterobacteriaceae_bacterium
A0A1B7HZ03_9ENT_Buttiauxella_noackiae
I2BAR8_SHIBC_Shimwellia_blattae
I0QLM0_9ENTR_Serratia_sp.
A0A0J8VJ79_9ENT_Cronobacter_sp.
AQPZ_PHOLL_Photorhabdus_luminescens
A0A1G5R2V6_PHOL_Photorhabdus_luminescens
U7R4L1_PHOTE_Photorhabdus_temperata
A0A1C0TZU2_9GAM_Photorhabdus_asymbiotica
A0A0N0URU9_9GAM_Photorhabdus_heterorhabditis
A0A085GSM0_HAFA_Hafnia_alvei
A0A1B7J529_9GAM_Hafnia_paralvei
A0A085G9H9_9ENT_Ewingella_americana
A0A1T4VMI2_PANA_Pantoea_ananas
A0A0H3L464_PANA_Pantoea_ananatis
A0A0M2YHZ6_PANA_Pantoea_ananas
H3RFK6_ERWST_Pantoea_stewartii
E6WLC3_PANSA_Pantoea_sp.
V5TX77_CROSK_Cronobacter_sakazakii
K8C2B4_9ENTR_Cronobacter_malonaticus
A7MKU3_ENTS8_Enterobacter_sakazakii
C9XXC2_CROTZ_Cronobacter_turicensis
K8AJI8_9ENTR_Cronobacter_dublinensis
Q9S0X6_PLESH_Plesiomonas_shigelloides
Q93PX2_SHISO_Shigella_sonnei
A0A0D5ITM4_9BUR_Burkholderia_dolosa
A2WE20_9BURK_Burkholderia_dolosa
A0A0B6X752_XENB_Xenorhabdus_bovienii
A0A077MVT6_XENB_Xenorhabdus_bovienii
A0A077PJL7_XENB_Xenorhabdus_bovienii
A0A077NGE0_XENB_Xenorhabdus_bovienii
A0A077NW66_XENB_Xenorhabdus_bovienii
W1IP08_9ENTR_Xenorhabdus_cabanillasii
A0A0X2NTK8_9GAM_Serratia_
A0A1Q5UZI4_SERM

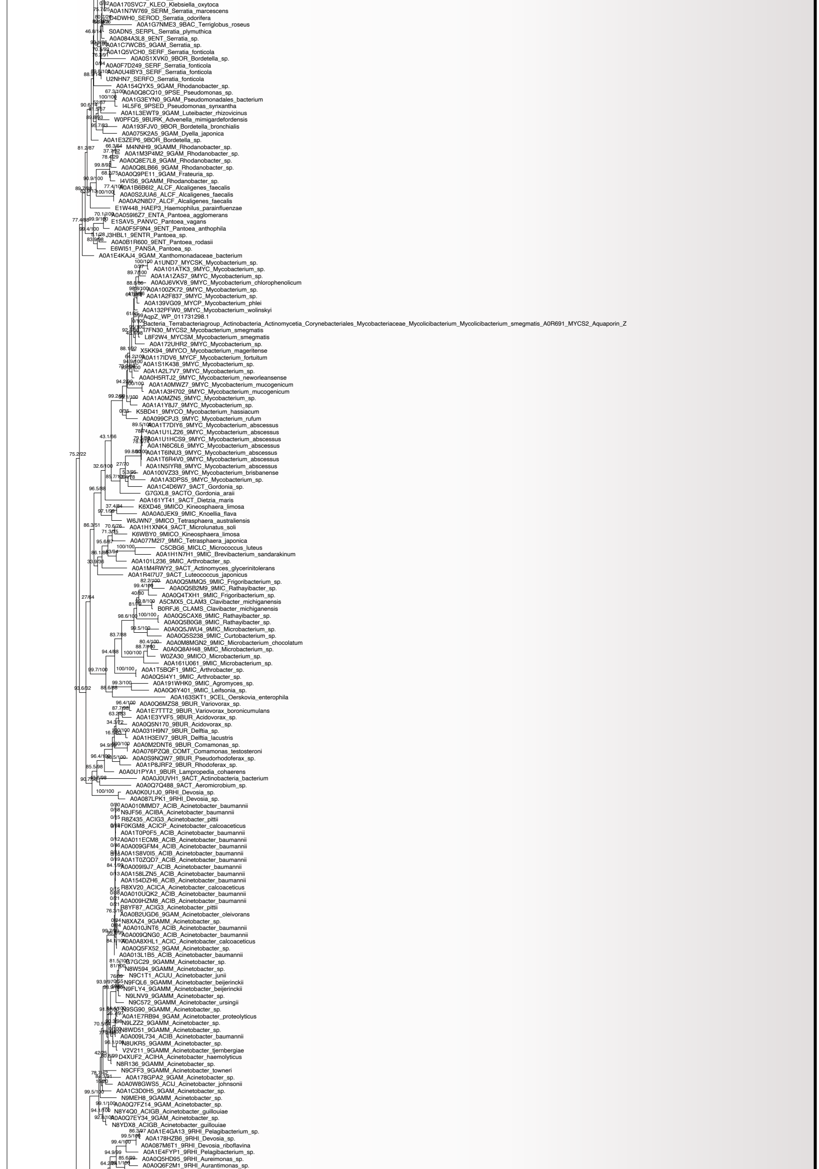
A0A170SVC7_KLEO_Klebsiella_oxytoca
A0A1N7W769_SERM_Serratia_marcescens
D4DWH0_SEROD_Serratia_odorifera
A0A1G7NME3_9BAC_Terriglobus_roseus
S0ADN5_SERPL_Serratia_plymuthica
A0A084A3L8_9ENT_Serratia_sp.
A0A1C7WCB5_9GAM_Serratia_sp.
A0A1Q5VCH0_SERF_Serratia_fonticola
A0A0S1XVK0_9BOR_Bordetella_sp.
A0A0F7D249_SERF_Serratia_fonticola
A0A0U4IBY3_SERF_Serratia_fonticola
U2NHN7_SERFO_Serratia_fonticola
A0A154QYX5_9GAM_Rhodanobacter_sp.
A0A0Q8CQ10_9PSE_Pseudomonas_sp.
A0A1G3EYN0_9GAM_Pseudomonadales_bacterium
I4L5F6_9PSED_Pseudomonas_synxantha
A0A1L3EWT9_9GAM_Luteibacter_rhizovicinus
W0PFQ5_9BURK_Advenella_mimigardefordensis
A0A193FJV0_9BOR_Bordetella_bronchialis
A0A075K2A5_9GAM_Dyella_japonica
A0A1E3ZEP6_9BOR_Bordetella_sp.
M4NNH9_9GAMM_Rhodanobacter_sp.
A0A1M3P4M2_9GAM_Rhodanobacter_sp.
A0A0Q8E7L8_9GAM_Rhodanobacter_sp.
A0A0Q8LB66_9GAM_Rhodanobacter_sp.
A0A0Q9PE11_9GAM_Frateuria_sp.
I4VIS6_9GAMM_Rhodanobacter_sp.
A0A1B6B6I2_ALCF_Alcaligenes_faecalis
A0A0S2JUA6_ALCF_Alcaligenes_faecalis
A0A0A2N8D7_ALCF_Alcaligenes_faecalis
E1W448_HAEP3_Haemophilus_parainfluenzae
A0A059I6Z7_ENTA_Pantoea_agglomerans
E1SAV5_PANVC_Pantoea_vagans
A0A0F5F9N4_9ENT_Pantoea_anthophila
J3HBL1_9ENTR_Pantoea_sp.
A0A0B1R600_9ENT_Pantoea_rodasii
E6WI51_PANSA_Pantoea_sp.
A0A1E4KAJ4_9GAM_Xanthomonadaceae_bacterium
A1UND7_MYCSK_Mycobacterium_sp.
A0A101ATK3_9MYC_Mycobacterium_sp.
A0A1A1ZAS7_9MYC_Mycobacterium_sp.
A0A0J6VKV8_9MYC_Mycobacterium_chlorophenolicum
A0A100ZK72_9MYC_Mycobacterium_sp.
A0A1A2F837_9MYC_Mycobacterium_sp.
A0A139VG09_MYCP_Mycobacterium_phlei
A0A132PFW0_9MYC_Mycobacterium_wolinskyi
AqpZ_WP_011731298.1
Bacteria_Terrabacteriagroup_Actinobacteria_Actinomycetia_Corynebacteriales_Mycobacteriaceae_Mycolicibacterium_Mycolicibacterium_smegmatis_A0R691_MYCS2_Aquaporin_Z
I7FN30_MYCS2_Mycobacterium_smegmatis
L8F2W4_MYCSM_Mycobacterium_smegmatis
A0A172UHR2_9MYC_Mycobacterium_sp.
X5KK94_9MYCO_Mycobacterium_mageritense
A0A117IDV6_MYCF_Mycobacterium_fortuitum
A0A1S1K438_9MYC_Mycobacterium_sp.
A0A1A2L7V7_9MYC_Mycobacterium_sp.
A0A0H5RTJ2_9MYC_Mycobacterium_neworleansense
A0A1A0MWZ7_9MYC_Mycobacterium_mucogenicum
A0A1A3H702_9MYC_Mycobacterium_mucogenicum
A0A1A0MZN5_9MYC_Mycobacterium_sp.
A0A1A1Y8J7_9MYC_Mycobacterium_sp.
K5BD41_9MYCO_Mycobacterium_hassiacum
A0A099CPJ3_9MYC_Mycobacterium_rufum
A0A1T7DIY6_9MYC_Mycobacterium_abscessus
A0A1U1LZ26_9MYC_Mycobacterium_abscessus
A0A1U1HCS9_9MYC_Mycobacterium_abscessus
A0A1N6C6L6_9MYC_Mycobacterium_abscessus
A0A1T6INU3_9MYC_Mycobacterium_abscessus
A0A1T6R4V0_9MYC_Mycobacterium_abscessus
A0A1N5IYR8_9MYC_Mycobacterium_abscessus
A0A100VZ33_9MYC_Mycobacterium_brisbanense
A0A1A3DPS5_9MYC_Mycobacterium_sp.
A0A1C4D6W7_9ACT_Gordonia_sp.
G7GXL8_9ACTO_Gordonia_araii
A0A161YT41_9ACT_Dietzia_maris
K6XD46_9MICO_Kineosphaera_limosa
A0A0A0JEK9_9MIC_Knoellia_flava
W6JWN7_9MICO_Tetrasphaera_australiensis
A0A1H1XNK4_9ACT_Microlunatus_soli
K6WBY0_9MICO_Kineosphaera_limosa
A0A077M2I7_9MIC_Tetrasphaera_japonica
C5CBG6_MICLC_Micrococcus_luteus
A0A1H1N7H1_9MIC_Brevibacterium_sandarakinum
A0A101L236_9MIC_Arthrobacter_sp.
A0A1M4RWY2_9ACT_Actinomyces_glycerinitolerans
A0A1R4I7U7_9ACT_Luteococcus_japonicus
A0A0Q5MMQ5_9MIC_Frigoribacterium_sp.
A0A0Q5B2M9_9MIC_Rathayibacter_sp.
A0A0Q4TXH1_9MIC_Frigoribacterium_sp.
A5CMX5_CLAM3_Clavibacter_michiganensis
B0RFJ6_CLAMS_Clavibacter_michiganensis
A0A0Q5CAX6_9MIC_Rathayibacter_sp.
A0A0Q5B0G8_9MIC_Rathayibacter_sp.
A0A0Q5JWU4_9MIC_Microbacterium_sp.
A0A0Q5S238_9MIC_Curtobacterium_sp.
A0A0M8MGN2_9MIC_Microbacterium_chocolatum
A0A0Q8AH48_9MIC_Microbacterium_sp.
W0ZA30_9MICO_Microbacterium_sp.
A0A161U061_9MIC_Microbacterium_sp.
A0A1T5BQF1_9MIC_Arthrobacter_sp.
A0A0Q5I4Y1_9MIC_Arthrobacter_sp.
A0A191WHK0_9MIC_Agromyces_sp.
A0A0Q6Y401_9MIC_Leifsonia_sp.
A0A163SKT1_9CEL_Oerskovia_enterophila
A0A0Q6MZS8_9BUR_Variovorax_sp.
A0A1E7TTT2_9BUR_Variovorax_boronicumulans
A0A1E3YVF5_9BUR_Acidovorax_sp.
A0A0Q5N170_9BUR_Acidovorax_sp.
A0A031H9N7_9BUR_Delftia_sp.
A0A1H3EIV7_9BUR_Delftia_lacustris
A0A0M2DNT6_9BUR_Comamonas_sp.
A0A076PZQ8_COMT_Comamonas_testosteroni
A0A0S9NQW7_9BUR_Pseudorhodoferax_sp.
A0A1P8JRF2_9BUR_Rhodoferax_sp.
A0A0U1PYA1_9BUR_Lampropedia_cohaerens
A0A0J0UVH1_9ACT_Actinobacteria_bacterium
A0A0Q7Q488_9ACT_Aeromicrobium_sp.
A0A0K0U1J0_9RHI_Devosia_sp.
A0A087LPK1_9RHI_Devosia_sp.
A0A010MMD7_ACIB_Acinetobacter_baumannii
N9JF56_ACIBA_Acinetobacter_baumannii
R8Z435_ACIG3_Acinetobacter_pittii
F0KGM8_ACICP_Acinetobacter_calcoaceticus
A0A1T0P0F5_ACIB_Acinetobacter_baumannii
A0A011ECM8_ACIB_Acinetobacter_baumannii
A0A009GFM4_ACIB_Acinetobacter_baumannii
A0A1S8V0I5_ACIB_Acinetobacter_baumannii
A0A1T0ZQD7_ACIB_Acinetobacter_baumannii
A0A009I9J7_ACIB_Acinetobacter_baumannii
A0A158LZN5_ACIB_Acinetobacter_baumannii
A0A154DZH6_ACIB_Acinetobacter_baumannii
R8XV20_ACICA_Acinetobacter_calcoaceticus
A0A010UQK2_ACIB_Acinetobacter_baumannii
A0A009HZM8_ACIB_Acinetobacter_baumannii
R8YF87_ACIG3_Acinetobacter_pittii
A0A0B2UGD6_9GAM_Acinetobacter_oleivorans
N8XAZ4_9GAMM_Acinetobacter_sp.
A0A010JNT6_ACIB_Acinetobacter_baumannii
A0A009QNG0_ACIB_Acinetobacter_baumannii
A0A0A8XHL1_ACIC_Acinetobacter_calcoaceticus
A0A0Q5FX52_9GAM_Acinetobacter_sp.
A0A013L1B5_ACIB_Acinetobacter_baumannii
S7GC29_9GAMM_Acinetobacter_sp.
N8W594_9GAMM_Acinetobacter_sp.
N9C1T1_ACIJU_Acinetobacter_junii
N9FQL6_9GAMM_Acinetobacter_beijerinckii
N9FLY4_9GAMM_Acinetobacter_beijerinckii
N9LNV9_9GAMM_Acinetobacter_sp.
N9C572_9GAMM_Acinetobacter_ursingii
N9SG90_9GAMM_Acinetobacter_sp.
A0A1E7RB94_9GAM_Acinetobacter_proteolyticus
N9LZZ2_9GAMM_Acinetobacter_sp.
N8WD51_9GAMM_Acinetobacter_sp.
A0A009L734_ACIB_Acinetobacter_baumannii
N8UKR5_9GAMM_Acinetobacter_sp.
V2V211_9GAMM_Acinetobacter_tjernbergiae
D4XUF2_ACIHA_Acinetobacter_haemolyticus
N8R136_9GAMM_Acinetobacter_sp.
N9CFF3_9GAMM_Acinetobacter_towneri
A0A178GPA2_9GAM_Acinetobacter_sp.
A0A0W8GWS5_ACIJ_Acinetobacter_johnsonii
A0A1C3D0H5_9GAM_Acinetobacter_sp.
N9MEH8_9GAMM_Acinetobacter_sp.
A0A0Q7FZ14_9GAM_Acinetobacter_sp.
N8Y4Q0_ACIGB_Acinetobacter_guillouiae
A0A0Q7EY34_9GAM_Acinetobacter_sp.
N8YDX8_ACIGB_Acinetobacter_guillouiae
A0A1E4GA13_9RHI_Pelagibacterium_sp.
A0A178HZB6_9RHI_Devosia_sp.
A0A087M6T1_9RHI_Devosia_riboflavina
A0A1E4FYP1_9RHI_Pelagibacterium_sp.
A0A0Q5HD95_9RHI_Aureimonas_sp.
A0A0Q6F2M1_9RHI_Aurantimonas_sp.

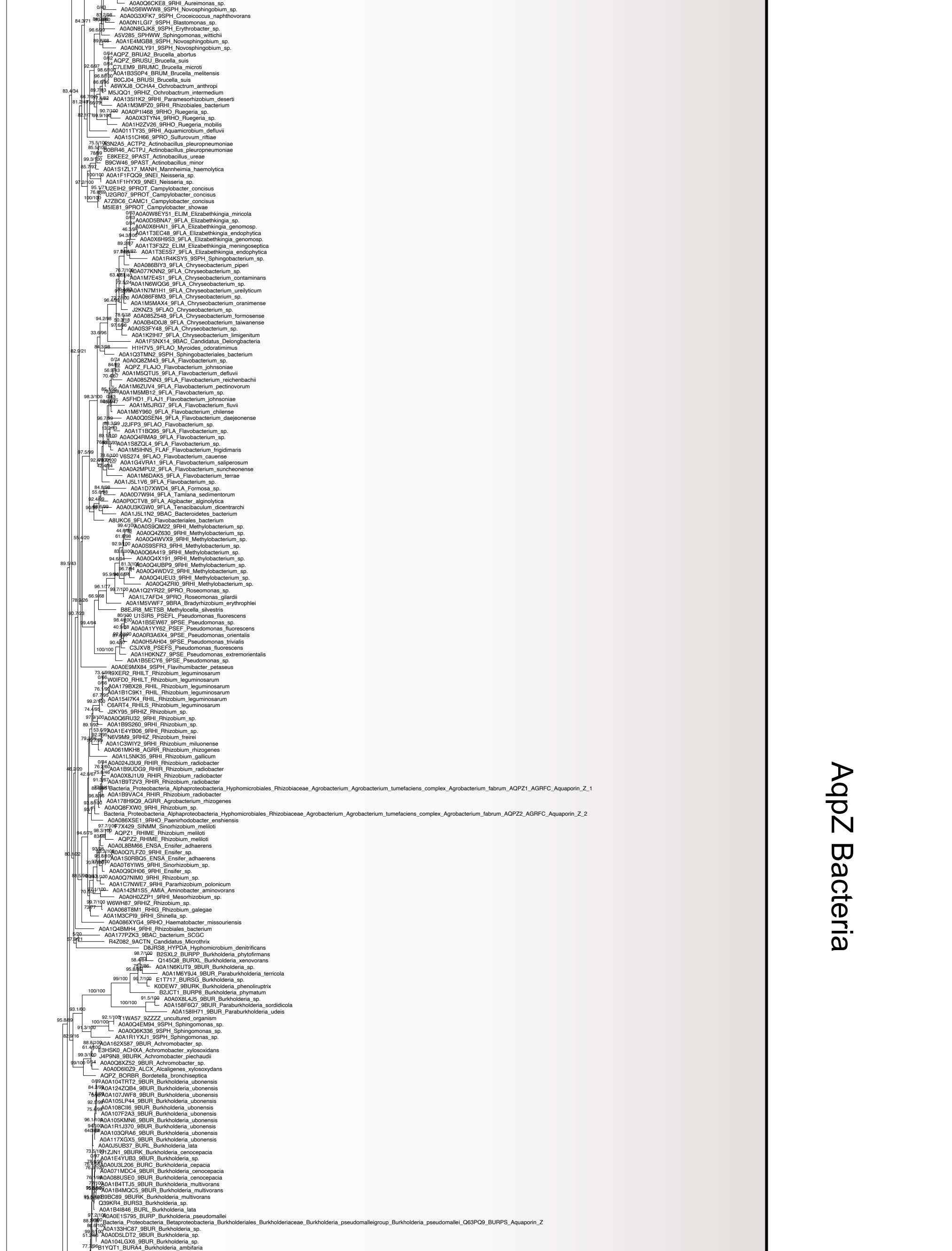
AqpZ Bacteria

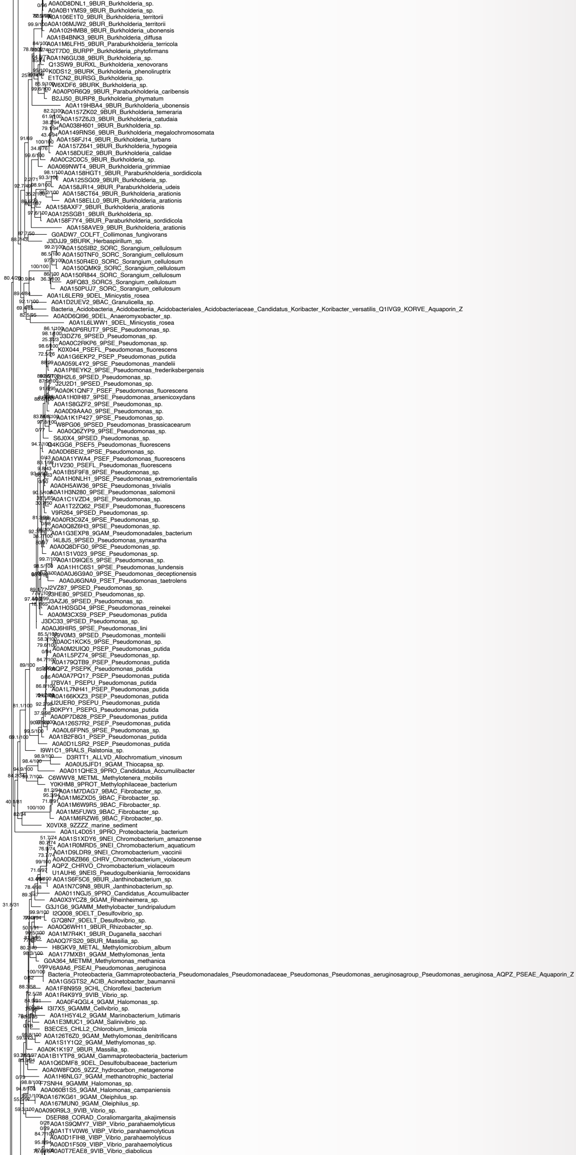
A0A0D8DNL1_9BUR_Burkholderia_sp.
A0A0B1YMS9_9BUR_Burkholderia_sp.
A0A106E1T0_9BUR_Burkholderia_territorii
A0A106MJW2_9BUR_Burkholderia_territorii
A0A102HMB8_9BUR_Burkholderia_ubonensis
A0A1B4BNK3_9BUR_Burkholderia_diffusa
A0A1M6LFH5_9BUR_Paraburkholderia_terricola
B2T7D0_BURPP_Burkholderia_phytofirmans
A0A1N6GU38_9BUR_Burkholderia_sp.
Q13SW9_BURXL_Burkholderia_xenovorans
K0DS12_9BURK_Burkholderia_phenoliruptrix
E1TCN2_BURSG_Burkholderia_sp.
W6XDF6_9BURK_Burkholderia_sp.
A0A0P0R6Q9_9BUR_Paraburkholderia_caribensis
B2JJ50_BURP8_Burkholderia_phymatum
A0A119HBA4_9BUR_Burkholderia_ubonensis
A0A157ZK02_9BUR_Burkholderia_temeraria
A0A157Z6J3_9BUR_Burkholderia_catudaia
A0A038H601_9BUR_Burkholderia_sp.
A0A149RNS6_9BUR_Burkholderia_megalochromosomata
A0A158FJ14_9BUR_Burkholderia_turbans
A0A157Z641_9BUR_Burkholderia_hypogeia
A0A158DUE2_9BUR_Burkholderia_calidae
A0A0C2C0C5_9BUR_Burkholderia_sp.
A0A069NWT4_9BUR_Burkholderia_grimmiae
A0A158HGT1_9BUR_Paraburkholderia_sordidicola
A0A125SG09_9BUR_Burkholderia_sp.
A0A158JR14_9BUR_Paraburkholderia_udeis
A0A158CT64_9BUR_Burkholderia_arationis
A0A158ELL0_9BUR_Burkholderia_arationis
A0A158AXF7_9BUR_Burkholderia_arationis
A0A125SGB1_9BUR_Burkholderia_sp.
A0A158F7Y4_9BUR_Paraburkholderia_sordidicola
A0A158AVE9_9BUR_Burkholderia_arationis
G0ADW7_COLFT_Collimonas_fungivorans
J3DJJ9_9BURK_Herbaspirillum_sp.
A0A150SIB2_SORC_Sorangium_cellulosum
A0A150TNF0_SORC_Sorangium_cellulosum
A0A150R4E0_SORC_Sorangium_cellulosum
A0A150QMK9_SORC_Sorangium_cellulosum
A0A150R844_SORC_Sorangium_cellulosum
A9FQ83_SORC5_Sorangium_cellulosum
A0A150PUJ7_SORC_Sorangium_cellulosum
A0A1L6LER9_9DEL_Minicystis_rosea
A0A1D2UEV2_9BAC_Granulicella_sp.
Bacteria_Acidobacteria_Acidobacteriia_Acidobacteriales_Acidobacteriaceae_Candidatus_Koribacter_Koribacter_versatilis_Q1IVG9_KORVE_Aquaporin_Z
A0A0D6QI96_9DEL_Anaeromyxobacter_sp.
A0A1L6LWW1_9DEL_Minicystis_rosea
A0A0P6RUT7_9PSE_Pseudomonas_sp.
J3DZ76_9PSED_Pseudomonas_sp.
A0A0C2RKP6_9PSE_Pseudomonas_sp.
K0X044_PSEFL_Pseudomonas_fluorescens
A0A1G6EKP2_PSEP_Pseudomonas_putida
A0A059L4Y2_9PSE_Pseudomonas_mandelii
A0A1P8EYK2_9PSE_Pseudomonas_frederiksbergensis
J3H2L6_9PSED_Pseudomonas_sp.
J2U2D1_9PSED_Pseudomonas_sp.
A0A0K1QNF7_PSEF_Pseudomonas_fluorescens
A0A1H0IH87_9PSE_Pseudomonas_arsenicoxydans
A0A1S8GZF2_9PSE_Pseudomonas_sp.
A0A0D9AAA0_9PSE_Pseudomonas_sp.
A0A1K1P427_9PSE_Pseudomonas_sp.
W8PG06_9PSED_Pseudomonas_brassicacearum
A0A0Q6ZYP9_9PSE_Pseudomonas_sp.
S6J0X4_9PSED_Pseudomonas_sp.
Q4KGG6_PSEF5_Pseudomonas_fluorescens
A0A0D6BEI2_9PSE_Pseudomonas_sp.
A0A0A1YWA4_PSEF_Pseudomonas_fluorescens
U1V230_PSEFL_Pseudomonas_fluorescens
A0A1B5F9F8_9PSE_Pseudomonas_sp.
A0A1H0NLH1_9PSE_Pseudomonas_extremorientalis
A0A0H5AW36_9PSE_Pseudomonas_trivialis
A0A1H3N280_9PSE_Pseudomonas_salomonii
A0A1C1VZD4_9PSE_Pseudomonas_sp.
A0A1T2ZQ62_PSEF_Pseudomonas_fluorescens
V9R264_9PSED_Pseudomonas_sp.
A0A0R3C9Z4_9PSE_Pseudomonas_sp.
A0A0Q8Z6H3_9PSE_Pseudomonas_sp.
A0A1G3EXP8_9GAM_Pseudomonadales_bacterium
I4L8J5_9PSED_Pseudomonas_synxantha
A0A0Q8DFG0_9PSE_Pseudomonas_sp.
A0A1S1V023_9PSE_Pseudomonas_sp.
A0A1D9IQE5_9PSE_Pseudomonas_sp.
A0A1H1C6S1_9PSE_Pseudomonas_lundensis
A0A0J6G9A0_9PSE_Pseudomonas_deceptionensis
A0A0J6GNA9_PSET_Pseudomonas_taetrolens
J2VZ87_9PSED_Pseudomonas_sp.
J3HE80_9PSED_Pseudomonas_sp.
I3AZJ6_9PSED_Pseudomonas_sp.
A0A1H0SGD4_9PSE_Pseudomonas_reinekei
A0A0M3CXS9_PSEP_Pseudomonas_putida
J3DC33_9PSED_Pseudomonas_sp.
A0A0J6HIR5_9PSE_Pseudomonas_lini
I9V0M3_9PSED_Pseudomonas_monteilii
A0A0C1KCK5_9PSE_Pseudomonas_sp.
A0A0M2UIQ0_PSEP_Pseudomonas_putida
A0A1L5PZ74_9PSE_Pseudomonas_sp.
A0A179QTB9_PSEP_Pseudomonas_putida
AQPZ_PSEPK_Pseudomonas_putida
A0A0A7PQ17_PSEP_Pseudomonas_putida
I7BVA1_PSEPU_Pseudomonas_putida
A0A1L7NH41_PSEP_Pseudomonas_putida
A0A166KXZ3_PSEP_Pseudomonas_putida
U2UER0_PSEPU_Pseudomonas_putida
B0KPY1_PSEPG_Pseudomonas_putida
A0A0P7D828_PSEP_Pseudomonas_putida
A0A126S7R2_PSEP_Pseudomonas_putida
A0A0L6FPN5_9PSE_Pseudomonas_sp.
A0A1B2F8G1_PSEP_Pseudomonas_putida
A0A0D1LSR2_PSEP_Pseudomonas_putida
I9W1C1_9RALS_Ralstonia_sp.
D3RTT1_ALLVD_Allochromatium_vinosum
A0A0U5JFD1_9GAM_Thiocapsa_sp.
A0A011QHE3_9PRO_Candidatus_Accumulibacter
C6WWV8_METML_Methylotenera_mobilis
Y0KHM8_9PROT_Methylophilaceae_bacterium
A0A1M7DAG7_9BAC_Fibrobacter_sp.
A0A1M6ZXD5_9BAC_Fibrobacter_sp.
A0A1M6W9R5_9BAC_Fibrobacter_sp.
A0A1M5FUW3_9BAC_Fibrobacter_sp.
A0A1M6RZW6_9BAC_Fibrobacter_sp.
X0VIX8_9ZZZZ_marine_sediment
A0A1L4D051_9PRO_Proteobacteria_bacterium
A0A1S1XDY6_9NEI_Chromobacterium_amazonense
A0A1R0MRD5_9NEI_Chromobacterium_aquaticum
A0A1D9LDR9_9NEI_Chromobacterium_vaccinii
A0A0D8ZB66_CHRV_Chromobacterium_violaceum
AQPZ_CHRVO_Chromobacterium_violaceum
U1AUH6_9NEIS_Pseudogulbenkiania_ferrooxidans
A0A1S6F5C6_9BUR_Janthinobacterium_sp.
A0A1N7C9N8_9BUR_Janthinobacterium_sp.
A0A011NGJ5_9PRO_Candidatus_Accumulibacter
A0A0X3YCZ8_9GAM_Rheinheimera_sp.
G3J1G6_9GAMM_Methylobacter_tundripaludum
I2Q008_9DELT_Desulfovibrio_sp.
G7Q8N7_9DELT_Desulfovibrio_sp.
A0A0Q6WH11_9BUR_Rhizobacter_sp.
A0A1M7R4K1_9BUR_Duganella_sacchari
A0A0Q7FS20_9BUR_Massilia_sp.
H8GKV9_METAL_Methylomicrobium_album
A0A177MXB1_9GAM_Methylomonas_lenta
G0A364_METMM_Methylomonas_methanica
V6A9A6_PSEAI_Pseudomonas_aeruginosa
Bacteria_Proteobacteria_Gammaproteobacteria_Pseudomonadales_Pseudomonadaceae_Pseudomonas_Pseudomonas_aeruginosagroup_Pseudomonas_aeruginosa_AQPZ_PSEAE_Aquaporin_Z
A0A1G5GTS2_ACIB_Acinetobacter_baumannii
A0A1F8N959_9CHL_Chloroflexi_bacterium
A0A1R4K9Y9_9VIB_Vibrio_sp.
A0A0F4QGL4_9GAM_Halomonas_sp.
I3I7X5_9GAMM_Cellvibrio_sp.
A0A1H5Y4L2_9GAM_Marinobacterium_lutimaris
A0A1E3MUC1_9GAM_Salinivibrio_sp.
B3ECE5_CHLL2_Chlorobium_limicola
A0A126T6Z0_9GAM_Methylomonas_denitrificans
A0A1S1Y1Q2_9GAM_Methylomonas_sp.
A0A0K1K197_9BUR_Massilia_sp.
A0A1B1YTP8_9GAM_Gammaproteobacteria_bacterium
A0A1Q6DMF8_9DEL_Desulfobulbaceae_bacterium
A0A0W8FQ05_9ZZZ_hydrocarbon_metagenome
A0A1H6NLG7_9GAM_methanotrophic_bacterial
F7SNH4_9GAMM_Halomonas_sp.
A0A060B1S5_9GAM_Halomonas_campaniensis
A0A167KG61_9GAM_Oleiphilus_sp.
A0A167MUN0_9GAM_Oleiphilus_sp.
A0A090R9L3_9VIB_Vibrio_sp.
D5ER88_CORAD_Coraliomargarita_akajimensis
A0A1S9QMY7_VIBP_Vibrio_parahaemolyticus
A0A1T1V0W6_VIBP_Vibrio_parahaemolyticus
A0A0D1FIH8_VIBP_Vibrio_parahaemolyticus
A0A0D1F509_VIBP_Vibrio_parahaemolyticus
A0A0T7EAE8_9VIB_Vibrio_diabolicus

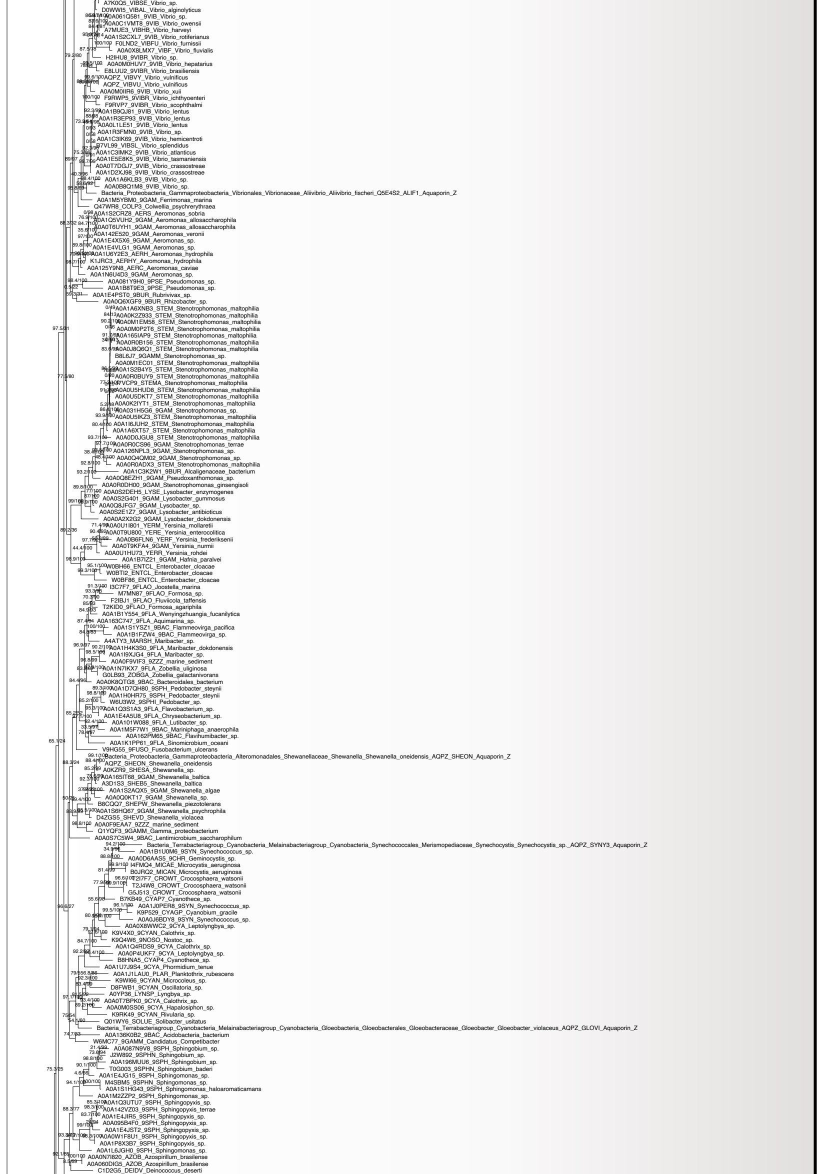
A7K0Q5_VIBSE_Vibrio_sp.
D0WWI5_VIBAL_Vibrio_alginolyticus
A0A061Q581_9VIB_Vibrio_sp.
A0A0C1VMT8_9VIB_Vibrio_owensii
A7MUE3_VIBHB_Vibrio_harveyi
A0A1S2CXL7_9VIB_Vibrio_rotiferianus
F0LND2_VIBFU_Vibrio_furnissii
A0A0X8LMX7_VIBF_Vibrio_fluvialis
H2IHU8_9VIBR_Vibrio_sp.
A0A0M0HUV7_9VIB_Vibrio_hepatarius
E8LUU2_9VIBR_Vibrio_brasiliensis
AQPZ_VIBVY_Vibrio_vulnificus
AQPZ_VIBVU_Vibrio_vulnificus
A0A0M0IIR6_9VIB_Vibrio_xuii
F9RWP5_9VIBR_Vibrio_ichthyoenteri
F9RVP7_9VIBR_Vibrio_scophthalmi
A0A1B9QJ81_9VIB_Vibrio_lentus
A0A1R3EP93_9VIB_Vibrio_lentus
A0A0L1LE51_9VIB_Vibrio_lentus
A0A1R3FMN0_9VIB_Vibrio_sp.
A0A1C3IK69_9VIB_Vibrio_hemicentroti
B7VL99_VIBSL_Vibrio_splendidus
A0A1C3IMK2_9VIB_Vibrio_atlanticus
A0A1E5E8K5_9VIB_Vibrio_tasmaniensis
A0A0T7DGJ7_9VIB_Vibrio_crassostreae
A0A1D2XJ98_9VIB_Vibrio_crassostreae
A0A1A6KLB3_9VIB_Vibrio_sp.
A0A0B8Q1M8_9VIB_Vibrio_sp.
Bacteria_Proteobacteria_Gammaproteobacteria_Vibrionales_Vibrionaceae_Aliivibrio_Aliivibrio_fischeri_Q5E4S2_ALIF1_Aquaporin_Z
A0A1M5YBM0_9GAM_Ferrimonas_marina
Q47WR8_COLP3_Colwellia_psychrerythraea
A0A1S2CRZ8_AERS_Aeromonas_sobria
A0A1Q5VUH2_9GAM_Aeromonas_allosaccharophila
A0A0T6UYH1_9GAM_Aeromonas_allosaccharophila
A0A142E520_9GAM_Aeromonas_veronii
A0A1E4X5X6_9GAM_Aeromonas_sp.
A0A1E4VLG1_9GAM_Aeromonas_sp.
A0A1U6Y2E3_AERH_Aeromonas_hydrophila
K1JRC3_AERHY_Aeromonas_hydrophila
A0A125Y9N8_AERC_Aeromonas_caviae
A0A1N6U4D3_9GAM_Aeromonas_sp.
A0A081Y9H0_9PSE_Pseudomonas_sp.
A0A1B8T9E3_9PSE_Pseudomonas_sp.
A0A1E4PST0_9BUR_Rubrivivax_sp.
A0A0Q6XGF9_9BUR_Rhizobacter_sp.
A0A1A6XNB3_STEM_Stenotrophomonas_maltophilia
A0A0K2Z933_STEM_Stenotrophomonas_maltophilia
A0A0M1EM58_STEM_Stenotrophomonas_maltophilia
A0A0M0P2T6_STEM_Stenotrophomonas_maltophilia
A0A165IAP9_STEM_Stenotrophomonas_maltophilia
A0A0R0B156_STEM_Stenotrophomonas_maltophilia
A0A0J8Q6Q1_STEM_Stenotrophomonas_maltophilia
B8L6J7_9GAMM_Stenotrophomonas_sp.
A0A0M1EC01_STEM_Stenotrophomonas_maltophilia
A0A1S2B4Y5_STEM_Stenotrophomonas_maltophilia
A0A0R0BUY9_STEM_Stenotrophomonas_maltophilia
B2FVCP9_STEMA_Stenotrophomonas_maltophilia
A0A0U5HUD8_STEM_Stenotrophomonas_maltophilia
A0A0U5DKT7_STEM_Stenotrophomonas_maltophilia
A0A0K2IYT1_STEM_Stenotrophomonas_maltophilia
A0A031H5G6_9GAM_Stenotrophomonas_sp.
A0A0U5IKZ3_STEM_Stenotrophomonas_maltophilia
A0A1I6JUH2_STEM_Stenotrophomonas_maltophilia
A0A1A6XT57_STEM_Stenotrophomonas_maltophilia
A0A0D0JGU8_STEM_Stenotrophomonas_maltophilia
A0A0R0CS96_9GAM_Stenotrophomonas_terrae
A0A126NPL3_9GAM_Stenotrophomonas_sp.
A0A0Q4QM02_9GAM_Stenotrophomonas_sp.
A0A0R0ADX3_STEM_Stenotrophomonas_maltophilia
A0A1C3K2W1_9BUR_Alcaligenaceae_bacterium
A0A0Q8EZH1_9GAM_Pseudoxanthomonas_sp.
A0A0R0DH00_9GAM_Stenotrophomonas_ginsengisoli
A0A0S2DEH5_LYSE_Lysobacter_enzymogenes
A0A0S2G401_9GAM_Lysobacter_gummosus
A0A0Q8JFG7_9GAM_Lysobacter_sp.
A0A0S2E1Z7_9GAM_Lysobacter_antibioticus
A0A0A2X2G2_9GAM_Lysobacter_dokdonensis
A0A0U1I801_YERM_Yersinia_mollaretii
A0A0T9U800_YERE_Yersinia_enterocolitica
A0A0B6FLN6_YERF_Yersinia_frederiksenii
A0A0T9KFA4_9GAM_Yersinia_nurmii
A0A0U1HU73_YERR_Yersinia_rohdei
A0A1B7IZ21_9GAM_Hafnia_paralvei
W0BH66_ENTCL_Enterobacter_cloacae
W0BTI2_ENTCL_Enterobacter_cloacae
W0BF86_ENTCL_Enterobacter_cloacae
I3C7F7_9FLAO_Joostella_marina
M7MN87_9FLAO_Formosa_sp.
F2IBJ1_9FLAO_Fluviicola_taffensis
T2KID0_9FLAO_Formosa_agariphila
A0A1B1Y554_9FLA_Wenyingzhuangia_fucanilytica
A0A163C747_9FLA_Aquimarina_sp.
A0A1S1YSZ1_9BAC_Flammeovirga_pacifica
A0A1B1FZW4_9BAC_Flammeovirga_sp.
A4ATY3_MARSH_Maribacter_sp.
A0A1H4K3S0_9FLA_Maribacter_dokdonensis
A0A1I9XJG4_9FLA_Maribacter_sp.
A0A0F9VIF3_9ZZZ_marine_sediment
A0A1N7IKX7_9FLA_Zobellia_uliginosa
G0LB93_ZOBGA_Zobellia_galactanivorans
A0A0K8QTG8_9BAC_Bacteroidales_bacterium
A0A1D7QH80_9SPH_Pedobacter_steynii
A0A1H0HR75_9SPH_Pedobacter_steynii
W6U3W2_9SPHI_Pedobacter_sp.
A0A1Q3S1A3_9FLA_Flavobacterium_sp.
A0A1E4A5U8_9FLA_Chryseobacterium_sp.
A0A101W088_9FLA_Lutibacter_sp.
A0A1M5F7W1_9BAC_Mariniphaga_anaerophila
A0A162PM65_9BAC_Flavihumibacter_sp.
A0A1K1PP61_9FLA_Sinomicrobium_oceani
V9HG55_9FUSO_Fusobacterium_ulcerans
Bacteria_Proteobacteria_Gammaproteobacteria_Alteromonadales_Shewanellaceae_Shewanella_Shewanella_oneidensis_AQPZ_SHEON_Aquaporin_Z
AQPZ_SHEON_Shewanella_oneidensis
A0KZR9_SHESA_Shewanella_sp.
A0A165IT68_9GAM_Shewanella_baltica
A3D1S3_SHEB5_Shewanella_baltica
A0A1S2AQX5_9GAM_Shewanella_algae
A0A0Q0KT17_9GAM_Shewanella_sp.
B8CQQ7_SHEPW_Shewanella_piezotolerans
A0A1S6HQ67_9GAM_Shewanella_psychrophila
D4ZGS5_SHEVD_Shewanella_violacea
A0A0F9EAA7_9ZZZ_marine_sediment
Q1YQF3_9GAMM_Gamma_proteobacterium
A0A0S7C5W4_9BAC_Lentimicrobium_saccharophilum
Bacteria_Terrabacteriagroup_Cyanobacteria_Melainabacteriagroup_Cyanobacteria_Synechococcales_Merismopediaceae_Synechocystis_Synechocystis_sp._AQPZ_SYNY3_Aquaporin_Z
A0A1B1U0M6_9SYN_Synechococcus_sp.
A0A0D6AAS5_9CHR_Geminocystis_sp.
I4FMQ4_MICAE_Microcystis_aeruginosa
B0JRQ2_MICAN_Microcystis_aeruginosa
T2I7F7_CROWT_Crocosphaera_watsonii
T2J4W8_CROWT_Crocosphaera_watsonii
G5J513_CROWT_Crocosphaera_watsonii
B7KB49_CYAP7_Cyanothece_sp.
A0A1J0PER8_9SYN_Synechococcus_sp.
K9P529_CYAGP_Cyanobium_gracile
A0A0J6BDY8_9SYN_Synechococcus_sp.
A0A0X8WWC2_9CYA_Leptolyngbya_sp.
K9V4X0_9CYAN_Calothrix_sp.
K9Q4W6_9NOSO_Nostoc_sp.
A0A1Q4RDS9_9CYA_Calothrix_sp.
A0A0P4UKF7_9CYA_Leptolyngbya_sp.
B8HNA5_CYAP4_Cyanothece_sp.
A0A1U7J9S4_9CYA_Phormidium_tenue
A0A1J1LAU0_PLAR_Planktothrix_rubescens
K9WI66_9CYAN_Microcoleus_sp.
D8FWB1_9CYAN_Oscillatoria_sp.
A0YP36_LYNSP_Lyngbya_sp.
A0A0T7BPK0_9CYA_Calothrix_sp.
A0A0M0SS06_9CYA_Hapalosiphon_sp.
K9RK49_9CYAN_Rivularia_sp.
Q01WY6_SOLUE_Solibacter_usitatus
Bacteria_Terrabacteriagroup_Cyanobacteria_Melainabacteriagroup_Cyanobacteria_Gloeobacteria_Gloeobacterales_Gloeobacteraceae_Gloeobacter_Gloeobacter_violaceus_AQPZ_GLOVI_Aquaporin_Z
A0A136K0B2_9BAC_Acidobacteria_bacterium
W6MC77_9GAMM_Candidatus_Competibacter
A0A087N9V8_9SPH_Sphingobium_sp.
J2W892_9SPHN_Sphingobium_sp.
A0A196MUU6_9SPH_Sphingobium_sp.
T0G003_9SPHN_Sphingobium_baderi
A0A1E4JG15_9SPH_Sphingomonas_sp.
M4SBM5_9SPHN_Sphingomonas_sp.
A0A1S1HG43_9SPH_Sphingomonas_haloaromaticamans
A0A1M2ZZP2_9SPH_Sphingomonas_sp.
A0A1Q3UTU7_9SPH_Sphingopyxis_sp.
A0A142VZ03_9SPH_Sphingopyxis_terrae
A0A1E4JIR5_9SPH_Sphingopyxis_sp.
A0A095B4F0_9SPH_Sphingopyxis_sp.
A0A1E4JST2_9SPH_Sphingopyxis_sp.
A0A0W1F8U1_9SPH_Sphingopyxis_sp.
A0A1P8X3B7_9SPH_Sphingopyxis_sp.
A0A1L6JGH0_9SPH_Sphingomonas_sp.
A0A0N7I820_AZOB_Azospirillum_brasilense
A0A060DIG5_AZOB_Azospirillum_brasilense
C1D2G5_DEIDV_Deinococcus_deserti

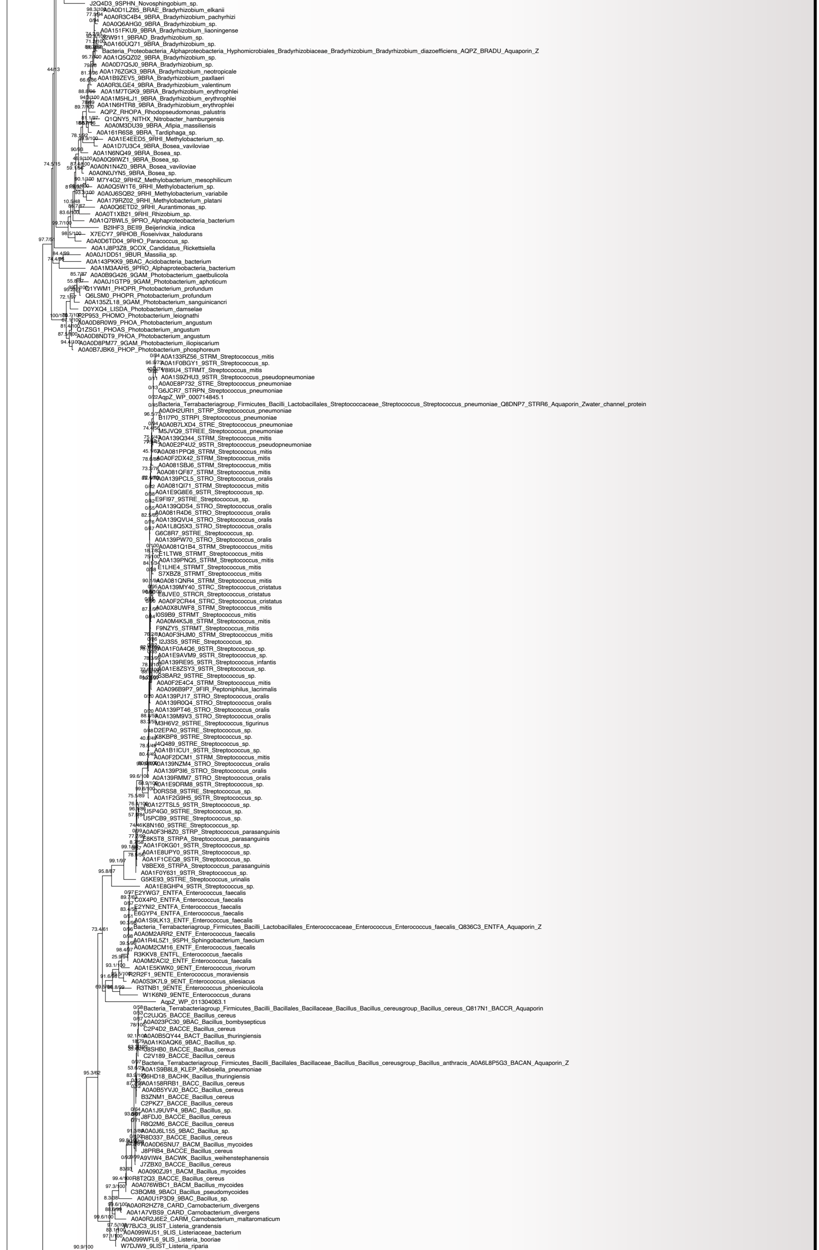
J2Q4D3_9SPHN_Novosphingobium_sp.
A0A0D1LZ85_BRAE_Bradyrhizobium_elkanii
A0A0R3C4B4_9BRA_Bradyrhizobium_pachyrhizi
A0A0Q6AHG0_9BRA_Bradyrhizobium_sp.
A0A151FKU9_9BRA_Bradyrhizobium_liaoningense
Q2W911_9BRAD_Bradyrhizobium_sp.
A0A160UQ71_9BRA_Bradyrhizobium_sp.
Bacteria_Proteobacteria_Alphaproteobacteria_Hyphomicrobiales_Bradyrhizobiaceae_Bradyrhizobium_Bradyrhizobium_diazoefficiens_AQPZ_BRADU_Aquaporin_Z
A0A1Q5QZ02_9BRA_Bradyrhizobium_sp.
A0A0D7Q5J0_9BRA_Bradyrhizobium_sp.
A0A176ZGK3_9BRA_Bradyrhizobium_neotropicale
A0A1B9ZEV5_9BRA_Bradyrhizobium_paxllaeri
A0A0R3LGE4_9BRA_Bradyrhizobium_valentinum
A0A1M7TGK9_9BRA_Bradyrhizobium_erythrophlei
A0A1M5HLJ1_9BRA_Bradyrhizobium_erythrophlei
A0A1N6HTR8_9BRA_Bradyrhizobium_erythrophlei
AQPZ_RHOPA_Rhodopseudomonas_palustris
Q1QNY5_NITHX_Nitrobacter_hamburgensis
A0A0M3DU39_9BRA_Afipia_massiliensis
A0A161R6S8_9BRA_Tardiphaga_sp.
A0A1E4EED5_9RHI_Methylobacterium_sp.
A0A1D7U3C4_9BRA_Bosea_vaviloviae
A0A1N6NQ49_9BRA_Bosea_sp.
A0A0Q9IWZ1_9BRA_Bosea_sp.
A0A0N1N4Z0_9BRA_Bosea_vaviloviae
A0A0N0JYN5_9BRA_Bosea_sp.
M7Y4G2_9RHIZ_Methylobacterium_mesophilicum
A0A0Q5W1T6_9RHI_Methylobacterium_sp.
A0A0J6SQB2_9RHI_Methylobacterium_variabile
A0A179RZ02_9RHI_Methylobacterium_platani
A0A0Q6ETD2_9RHI_Aurantimonas_sp.
A0A0T1XB21_9RHI_Rhizobium_sp.
A0A1Q7BWL5_9PRO_Alphaproteobacteria_bacterium
B2IHF3_BEII9_Beijerinckia_indica
X7ECY7_9RHOB_Roseivivax_halodurans
A0A0D6TD04_9RHO_Paracoccus_sp.
A0A1J8P3Z8_9COX_Candidatus_Rickettsiella
A0A0J1DD51_9BUR_Massilia_sp.
A0A143PKK9_9BAC_Acidobacteria_bacterium
A0A1M3AAH5_9PRO_Alphaproteobacteria_bacterium
A0A0B9G426_9GAM_Photobacterium_gaetbulicola
A0A0J1GTP9_9GAM_Photobacterium_aphoticum
Q1YWM1_PHOPR_Photobacterium_profundum
Q6LSM0_PHOPR_Photobacterium_profundum
A0A135ZL18_9GAM_Photobacterium_sanguinicancri
D0YXQ4_LISDA_Photobacterium_damselae
P2P953_PHOMO_Photobacterium_leiognathi
A0A0D8R0W9_PHOA_Photobacterium_angustum
Q1ZSG1_PHOAS_Photobacterium_angustum
A0A0D8NDT9_PHOA_Photobacterium_angustum
A0A0D8PM77_9GAM_Photobacterium_iliopiscarium
A0A0B7JBK6_PHOP_Photobacterium_phosphoreum
A0A133RZ56_STRM_Streptococcus_mitis
A0A1F0BGY1_9STR_Streptococcus_sp.
V8I6U4_STRMT_Streptococcus_mitis
A0A1S9ZHU3_9STR_Streptococcus_pseudopneumoniae
A0A0E8P732_STRE_Streptococcus_pneumoniae
G6JCR7_STRPN_Streptococcus_pneumoniae
AqpZ_WP_000714845.1
Bacteria_Terrabacteriagroup_Firmicutes_Bacilli_Lactobacillales_Streptococcaceae_Streptococcus_Streptococcus_pneumoniae_Q8DNP7_STRR6_Aquaporin_Zwater_channel_protein
A0A0H2URI1_STRP_Streptococcus_pneumoniae
B1I7P0_STRPI_Streptococcus_pneumoniae
A0A0B7LXD4_STRE_Streptococcus_pneumoniae
M5JVQ9_STREE_Streptococcus_pneumoniae
A0A139Q344_STRM_Streptococcus_mitis
A0A0E2P4U2_9STR_Streptococcus_pseudopneumoniae
A0A081PPQ8_STRM_Streptococcus_mitis
A0A0F2DX42_STRM_Streptococcus_mitis
A0A081SBJ6_STRM_Streptococcus_mitis
A0A081QF87_STRM_Streptococcus_mitis
A0A139PCL5_STRO_Streptococcus_oralis
A0A081QI71_STRM_Streptococcus_mitis
A0A1E9G8E6_9STR_Streptococcus_sp.
E9FI97_9STRE_Streptococcus_sp.
A0A139QDS4_STRO_Streptococcus_oralis
A0A081R4D6_STRO_Streptococcus_oralis
A0A139QVU4_STRO_Streptococcus_oralis
A0A1L8Q5X3_STRO_Streptococcus_oralis
G6C8R7_9STRE_Streptococcus_sp.
A0A139PW70_STRO_Streptococcus_oralis
A0A081Q1B4_STRM_Streptococcus_mitis
E1LTW8_STRMT_Streptococcus_mitis
A0A139PNQ5_STRM_Streptococcus_mitis
E1LHE4_STRMT_Streptococcus_mitis
S7XBZ8_STRMT_Streptococcus_mitis
A0A081QNR4_STRM_Streptococcus_mitis
A0A139MY40_STRC_Streptococcus_cristatus
E8JVE0_STRCR_Streptococcus_cristatus
A0A0F2CR44_STRC_Streptococcus_cristatus
A0A0X8UWF8_STRM_Streptococcus_mitis
I0S9B9_STRMT_Streptococcus_mitis
A0A0M4K5J8_STRM_Streptococcus_mitis
F9NZY5_STRMT_Streptococcus_mitis
A0A0F3HJM0_STRM_Streptococcus_mitis
I2J3S5_9STRE_Streptococcus_sp.
A0A1F0A4Q6_9STR_Streptococcus_sp.
A0A1E9AVM9_9STR_Streptococcus_sp.
A0A139RE95_9STR_Streptococcus_infantis
A0A1E8ZSY3_9STR_Streptococcus_sp.
S3BAR2_9STRE_Streptococcus_sp.
A0A0F2E4C4_STRM_Streptococcus_mitis
A0A096B9P7_9FIR_Peptoniphilus_lacrimalis
A0A139PJ17_STRO_Streptococcus_oralis
A0A139R0Q4_STRO_Streptococcus_oralis
A0A139PT46_STRO_Streptococcus_oralis
A0A139M9V3_STRO_Streptococcus_oralis
M3H6V2_9STRE_Streptococcus_tigurinus
D2EPA0_9STRE_Streptococcus_sp.
X8KBP8_9STRE_Streptococcus_sp.
J4Q489_9STRE_Streptococcus_sp.
A0A1B1ICU1_9STR_Streptococcus_sp.
A0A0F2DCM1_STRM_Streptococcus_mitis
A0A139NZM4_STRO_Streptococcus_oralis
A0A139P3I6_STRO_Streptococcus_oralis
A0A139RMM7_STRO_Streptococcus_oralis
A0A1E9DRM8_9STR_Streptococcus_sp.
D0RSS8_9STRE_Streptococcus_sp.
A0A1F2G9H5_9STR_Streptococcus_sp.
A0A127TSL5_9STR_Streptococcus_sp.
U5P4G0_9STRE_Streptococcus_sp.
U5PCB9_9STRE_Streptococcus_sp.
K8N160_9STRE_Streptococcus_sp.
A0A0F3H8Z0_STRP_Streptococcus_parasanguinis
E8K5T8_STRPA_Streptococcus_parasanguinis
A0A1F0KG01_9STR_Streptococcus_sp.
A0A1E8UPY0_9STR_Streptococcus_sp.
A0A1F1CEQ8_9STR_Streptococcus_sp.
V8BEX6_STRPA_Streptococcus_parasanguinis
A0A1F0Y631_9STR_Streptococcus_sp.
G5KE93_9STRE_Streptococcus_urinalis
A0A1E8GHP4_9STR_Streptococcus_sp.
E2YWG7_ENTFA_Enterococcus_faecalis
C0X4P0_ENTFA_Enterococcus_faecalis
E2YNI2_ENTFA_Enterococcus_faecalis
E6GYP4_ENTFA_Enterococcus_faecalis
A0A1S9LK13_ENTF_Enterococcus_faecalis
Bacteria_Terrabacteriagroup_Firmicutes_Bacilli_Lactobacillales_Enterococcaceae_Enterococcus_Enterococcus_faecalis_Q836C3_ENTFA_Aquaporin_Z
A0A0M2ARR2_ENTF_Enterococcus_faecalis
A0A1R4L5Z1_9SPH_Sphingobacterium_faecium
A0A0M2CM16_ENTF_Enterococcus_faecalis
R3KKV8_ENTFL_Enterococcus_faecalis
A0A0M2ACI2_ENTF_Enterococcus_faecalis
A0A1E5KWK0_9ENT_Enterococcus_rivorum
R2R2F1_9ENTE_Enterococcus_moraviensis
A0A0S3K7L9_9ENT_Enterococcus_silesiacus
R3TNB1_9ENTE_Enterococcus_phoeniculicola
W1K6N9_9ENTE_Enterococcus_durans
AqpZ_WP_011304063.1
Bacteria_Terrabacteriagroup_Firmicutes_Bacilli_Bacillales_Bacillaceae_Bacillus_Bacillus_cereusgroup_Bacillus_cereus_Q817N1_BACCR_Aquaporin
C2UJQ5_BACCE_Bacillus_cereus
A0A023PC30_9BAC_Bacillus_bombysepticus
C2P4D2_BACCE_Bacillus_cereus
A0A0B5QY44_BACT_Bacillus_thuringiensis
A0A1K0AQK6_9BAC_Bacillus_sp.
J8SHB0_BACCE_Bacillus_cereus
C2V189_BACCE_Bacillus_cereus
Bacteria_Terrabacteriagroup_Firmicutes_Bacilli_Bacillales_Bacillaceae_Bacillus_Bacillus_cereusgroup_Bacillus_anthracis_A0A6L8P5G3_BACAN_Aquaporin_Z
A0A1S9B8L8_KLEP_Klebsiella_pneumoniae
Q6HD18_BACHK_Bacillus_thuringiensis
A0A158RRB1_BACC_Bacillus_cereus
A0A0B5YVJ0_BACC_Bacillus_cereus
B3ZNM1_BACCE_Bacillus_cereus
C2PKZ7_BACCE_Bacillus_cereus
A0A1J9UVP4_9BAC_Bacillus_sp.
J8FDJ0_BACCE_Bacillus_cereus
R8Q2M6_BACCE_Bacillus_cereus
A0A0J6L155_9BAC_Bacillus_sp.
R8D337_BACCE_Bacillus_cereus
A0A0D6SNU7_BACM_Bacillus_mycoides
J8PRB4_BACCE_Bacillus_cereus
A9VIW4_BACWK_Bacillus_weihenstephanensis
J7ZBX0_BACCE_Bacillus_cereus
A0A090ZJ91_BACM_Bacillus_mycoides
R8T2Q3_BACCE_Bacillus_cereus
A0A076WBC1_BACM_Bacillus_mycoides
C3BQM8_9BACI_Bacillus_pseudomycoides
A0A0U1P3D9_9BAC_Bacillus_sp.
A0A0R2HZ78_CARD_Carnobacterium_divergens
A0A1A7VBS9_CARD_Carnobacterium_divergens
A0A0R2J6E2_CARM_Carnobacterium_maltaromaticum
W7BJC3_9LIST_Listeria_grandensis
A0A099WJ51_9LIS_Listeriaceae_bacterium
A0A099WFL6_9LIS_Listeria_booriae
W7DJW9_9LIST_Listeria_riparia

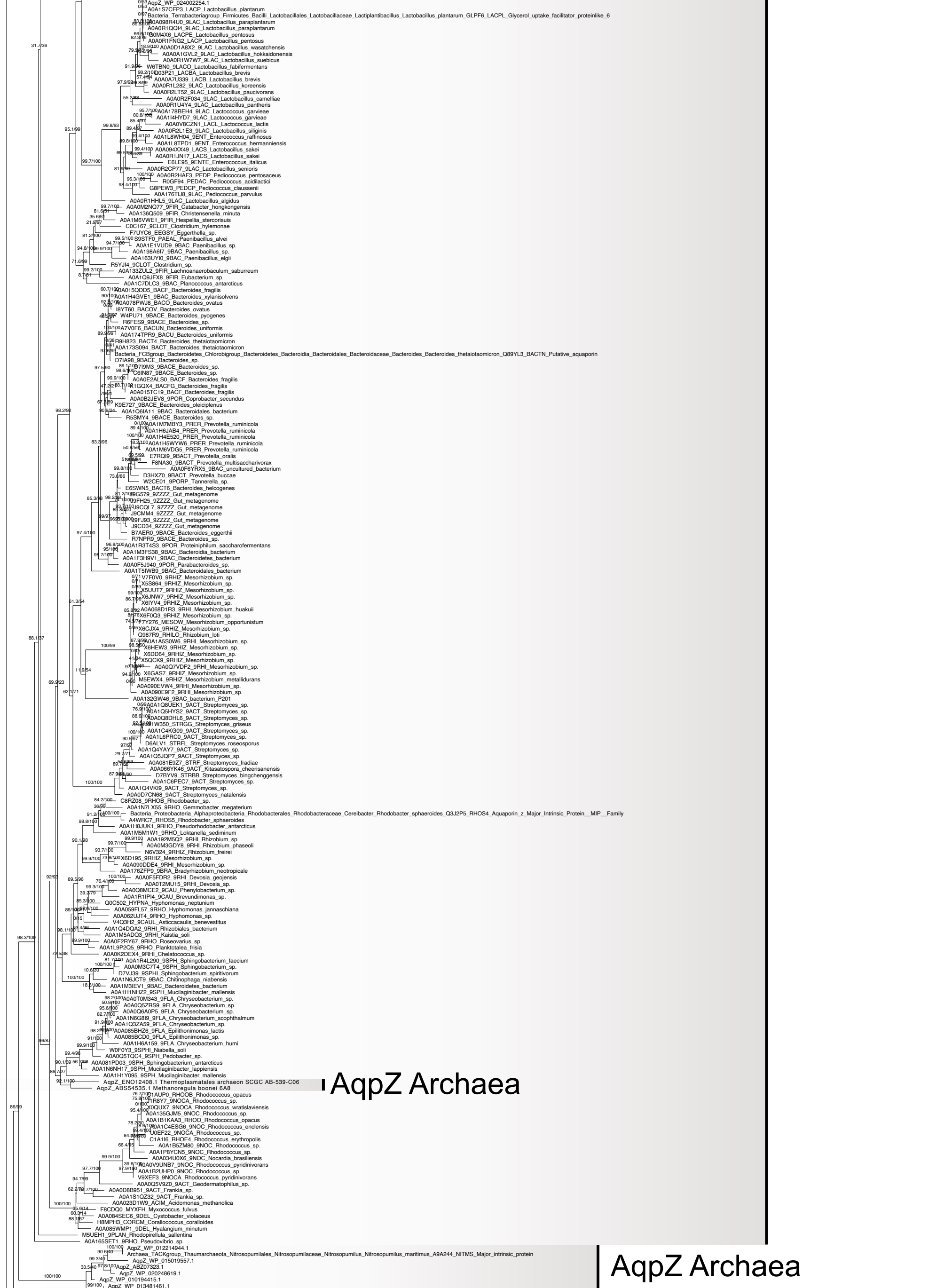
AqpZ Archaea
AqpZ Archaea

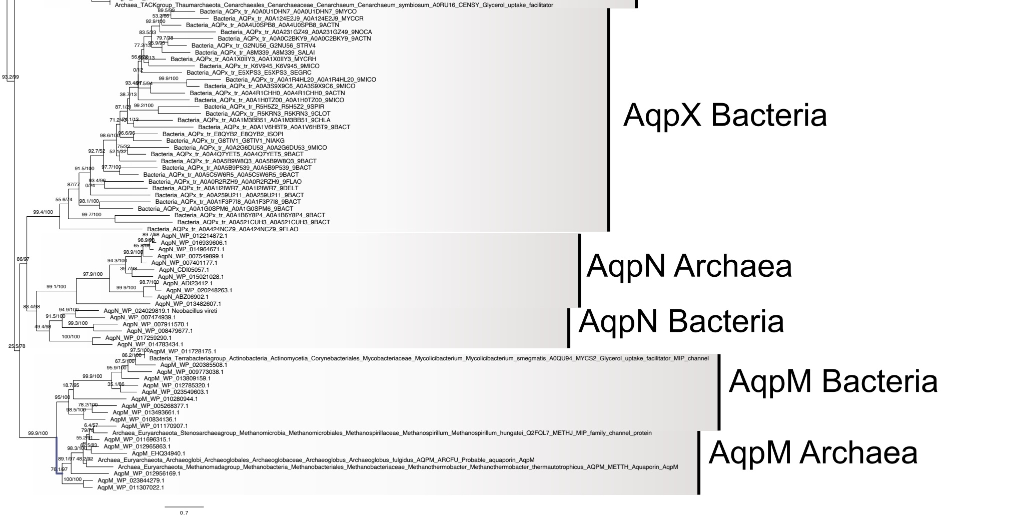
Archaea_TACKgroup_Thaumarchaeota_Cenarchaeales_Cenarchaeaceae_Cenarchaeum_Cenarchaeum_symbiosum_A0RU16_CENSY_Glycerol_uptake_facilitator
Bacteria_AQPx_tr_A0A0U1DHN7_A0A0U1DHN7_9MYCO
Bacteria_AQPx_tr_A0A124E2J9_A0A124E2J9_MYCCR
Bacteria_AQPx_tr_A0A4U0SPB8_A0A4U0SPB8_9ACTN
Bacteria_AQPx_tr_A0A231GZ49_A0A231GZ49_9NOCA
Bacteria_AQPx_tr_A0A0C2BKY9_A0A0C2BKY9_9ACTN
Bacteria_AQPx_tr_G2NU56_G2NU56_STRV4
Bacteria_AQPx_tr_A8M339_A8M339_SALAI
Bacteria_AQPx_tr_A0A1X0IIY3_A0A1X0IIY3_MYCRH
Bacteria_AQPx_tr_K6V945_K6V945_9MICO
Bacteria_AQPx_tr_E5XPS3_E5XPS3_SEGRC
Bacteria_AQPx_tr_A0A1R4HL20_A0A1R4HL20_9MICO
Bacteria_AQPx_tr_A0A3S9X9C6_A0A3S9X9C6_9MICO
Bacteria_AQPx_tr_A0A4R1CHH0_A0A4R1CHH0_9ACTN
Bacteria_AQPx_tr_A0A1H0TZ00_A0A1H0TZ00_9MICO
Bacteria_AQPx_tr_R5H5Z2_R5H5Z2_9SPIR
Bacteria_AQPx_tr_R5KRN3_R5KRN3_9CLOT
Bacteria_AQPx_tr_A0A1M3BB51_A0A1M3BB51_9CHLA
Bacteria_AQPx_tr_A0A1V6HBT9_A0A1V6HBT9_9BACT
Bacteria_AQPx_tr_E8QYB2_E8QYB2_ISOPI
Bacteria_AQPx_tr_G8TIV1_G8TIV1_NIAKG
Bacteria_AQPx_tr_A0A2G6DU53_A0A2G6DU53_9MICO
Bacteria_AQPx_tr_A0A4Q7YET5_A0A4Q7YET5_9BACT
Bacteria_AQPx_tr_A0A5B9W8Q3_A0A5B9W8Q3_9BACT
Bacteria_AQPx_tr_A0A5B9P539_A0A5B9P539_9BACT
Bacteria_AQPx_tr_A0A5C5W6R5_A0A5C5W6R5_9BACT
Bacteria_AQPx_tr_A0A0R2RZH9_A0A0R2RZH9_9FLAO
Bacteria_AQPx_tr_A0A1I2IWR7_A0A1I2IWR7_9DELT
Bacteria_AQPx_tr_A0A259U211_A0A259U211_9BACT
Bacteria_AQPx_tr_A0A1F3P7I8_A0A1F3P7I8_9BACT
Bacteria_AQPx_tr_A0A1G0SPM6_A0A1G0SPM6_9BACT
Bacteria_AQPx_tr_A0A1B6Y8P4_A0A1B6Y8P4_9BACT
Bacteria_AQPx_tr_A0A521CUH3_A0A521CUH3_9BACT
Bacteria_AQPx_tr_A0A424NCZ9_A0A424NCZ9_9FLAO
AqpX Bacteria
AqpN_WP_012214872.1
AqpN_WP_016939606.1
AqpN_WP_014964671.1
AqpN_WP_007549899.1
AqpN_WP_007401177.1
AqpN_CDI05057.1
AqpN_WP_015021028.1
AqpN_ADI23412.1
AqpN_WP_020248263.1
AqpN_ABZ06902.1
AqpN_WP_013482607.1
AqpN Archaea
AqpN_WP_024029819.1 Neobacillus vireti
AqpN_WP_007474939.1
AqpN_WP_007911570.1
AqpN_WP_008479677.1
AqpN_WP_017259290.1
AqpN_WP_014783434.1
AqpN Bacteria
AqpM_WP_011728175.1
AqpM_WP_011728190.1
Bacteria_Terrabacteriagroup_Actinobacteria_Actinomycetia_Corynebacteriales_Mycobacteriaceae_Mycolicibacterium_Mycolicibacterium_smegmatis_A0QU94_MYCS2_Glycerol_uptake_facilitator_MIP_channel
AqpM_WP_020385508.1
AqpM_WP_009773038.1
AqpM_WP_013809159.1
AqpM_WP_012785320.1
AqpM_WP_023549603.1
AqpM_WP_010280944.1
AqpM_WP_005268377.1
AqpM_WP_013493661.1
AqpM_WP_010834136.1
AqpM Bacteria
AqpM_WP_011170907.1
Archaea_Euryarchaeota_Stenosarchaeagroup_Methanomicrobia_Methanomicrobiales_Methanospirillaceae_Methanospirillum_Methanospirillum_hungatei_Q2FQL7_METHJ_MIP_family_channel_protein
AqpM_WP_011696315.1
AqpM_WP_012965863.1
AqpM_EHQ34940.1
Archaea_Euryarchaeota_Archaeoglobi_Archaeoglobales_Archaeoglobaceae_Archaeoglobus_Archaeoglobus_fulgidus_AQPM_ARCFU_Probable_aquaporin_AqpM
Archaea_Euryarchaeota_Methanomadagroup_Methanobacteria_Methanobacteriales_Methanobacteriaceae_Methanothermobacter_Methanothermobacter_thermautotrophicus_AQPM_METTH_Aquaporin_AqpM
AqpM_WP_012956169.1
AqpM_WP_023844279.1
AqpM_WP_011307022.1
AqpM Archaea
0.7

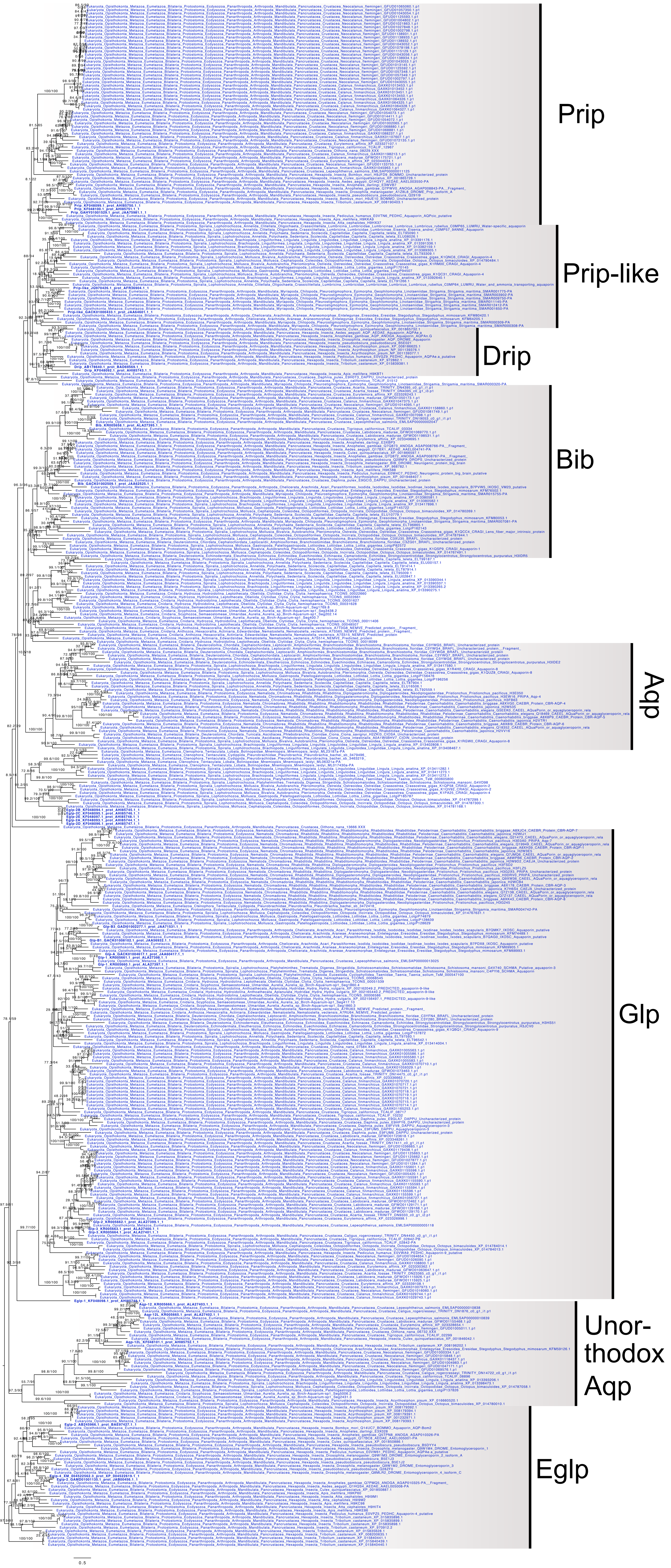
Prip
Prip-like
Drip
Bib
Aqp
Glp
Unor-
thodox
Aqp
Eglp
0.5

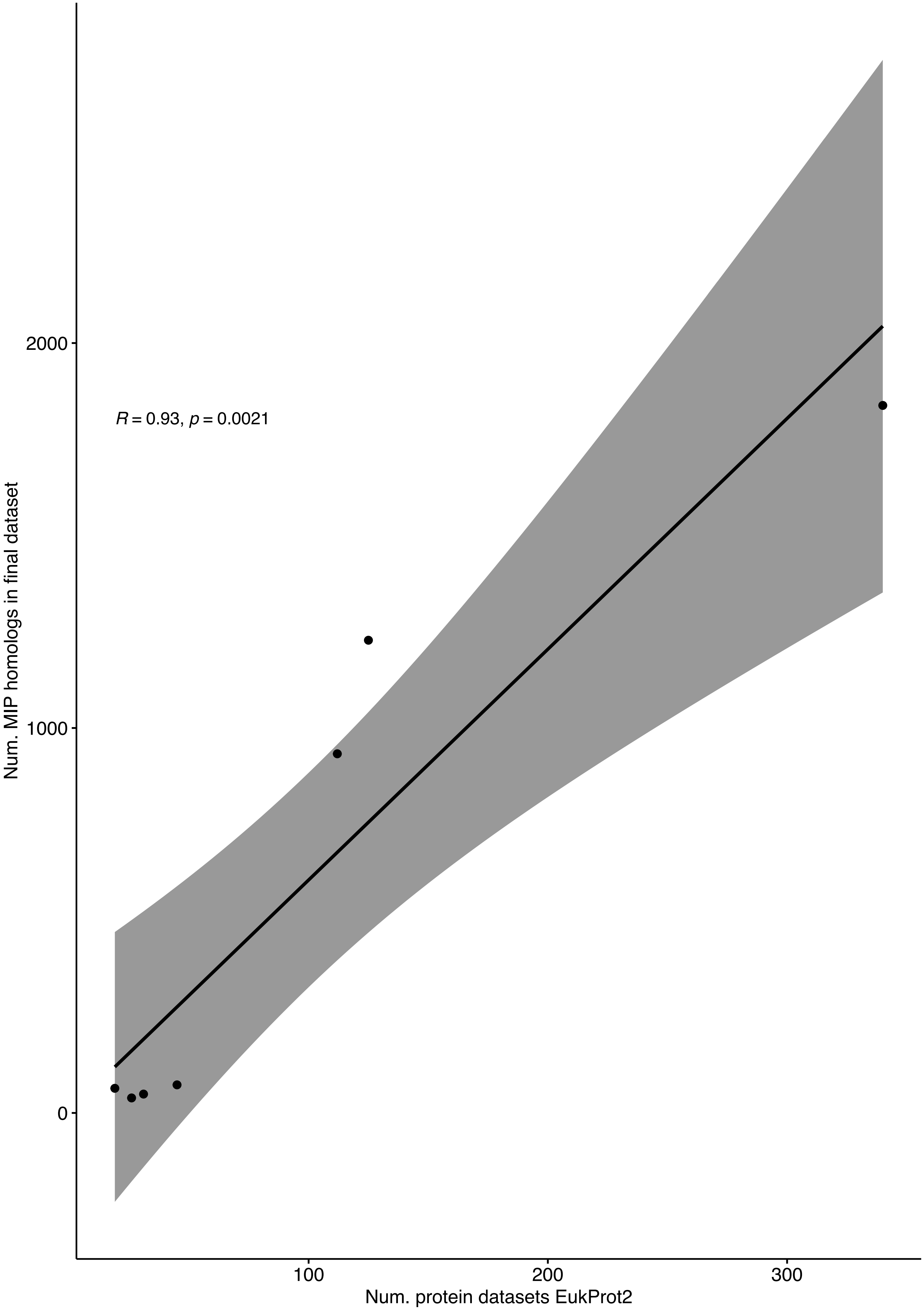
R = 0.93, p = 0.0021
2000
1000
0
100
200
300
Num. MIP homologs in final dataset
Num. protein datasets EukProt2

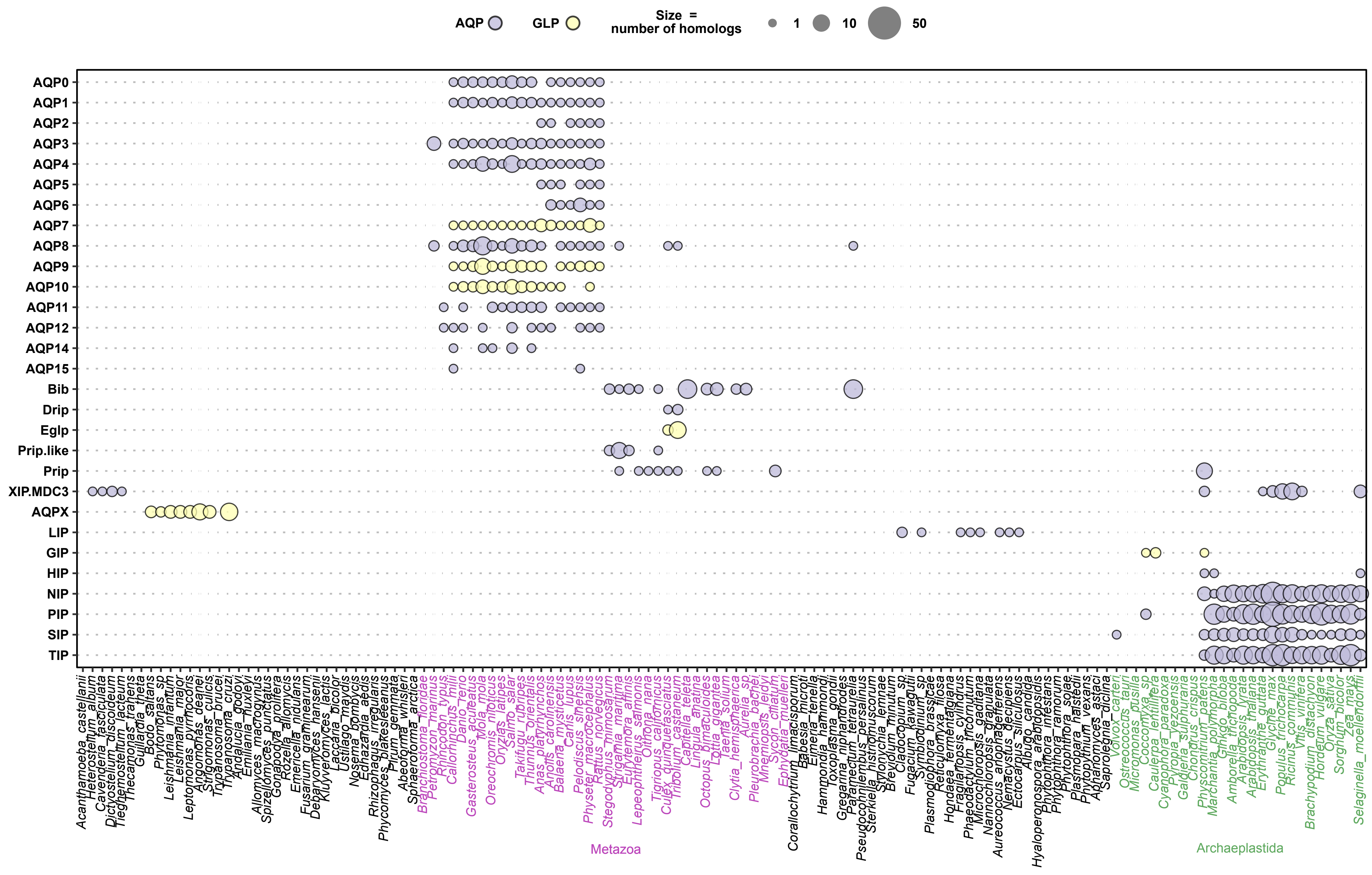
AQP
GLP
Size = number of homologs
1
10
50
AQP0
AQP1
AQP2
AQP3
AQP4
AQP5
AQP6
AQP7
AQP8
AQP9
AQP10
AQP11
AQP12
AQP14
AQP15
Bib
Drip
Eglp
Prip.like
Prip
XIP.MDC3
AQPX
LIP
GIP
HIP
NIP
PIP
SIP
TIP
Metazoa
Archaeplastida
